# Supplementary material for: A genetic toolkit and gene switches to limit Mycoplasma growth for biosafety applications
Source: Nat Commun. 2022 Apr 7;13:1910. doi: 10.1038/s41467-022-29574-0 (PMC8991246; doi:10.1038/s41467-022-29574-0)

Supplementary Material

A genetic toolkit and gene switches to limit *Mycoplasma* growth for biosafety applications

Alicia Broto[1], Erika Gaspari[2,3], Samuel Miravet-Verde[4], Vitor A.P. Martins dos Santos[2,5] and Mark Isalan[1]*

Supplementary figures

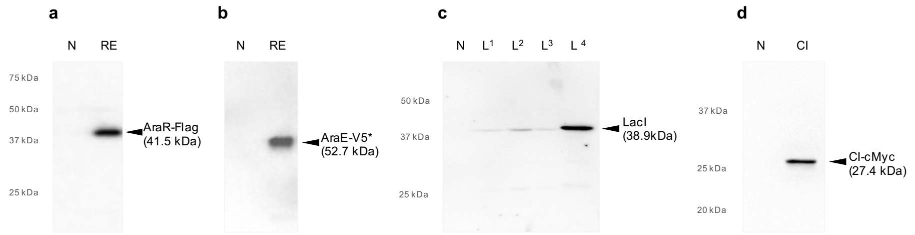

Suppl. Fig. 1. Western blot results showing successful expression of the proteins AraR (**a**), AraE (**b**), LacI (**c**) and CI (**d**) in *M. pneumoniae*. Expression of the proteins was analysed using 10 µg of total protein extracts prepared from strains generated by transformation with an MTn*cat* with the following cassettes: p438-*araR*-flag-tag and p438-*araE*-V5-tag (RE); p438-*lacI* cassette (L1); pS-*lacI* (L2); pS-RBS-*lacI* (L3); pS-LP-*lacI* (L4); and p438-*cI* cassette. Wilt-type strain M129 (N) included as a negative control. Strains generated with this MTn*cat* were selected with chloramphenicol. Western blot results are representative of two independent experiments in each case.

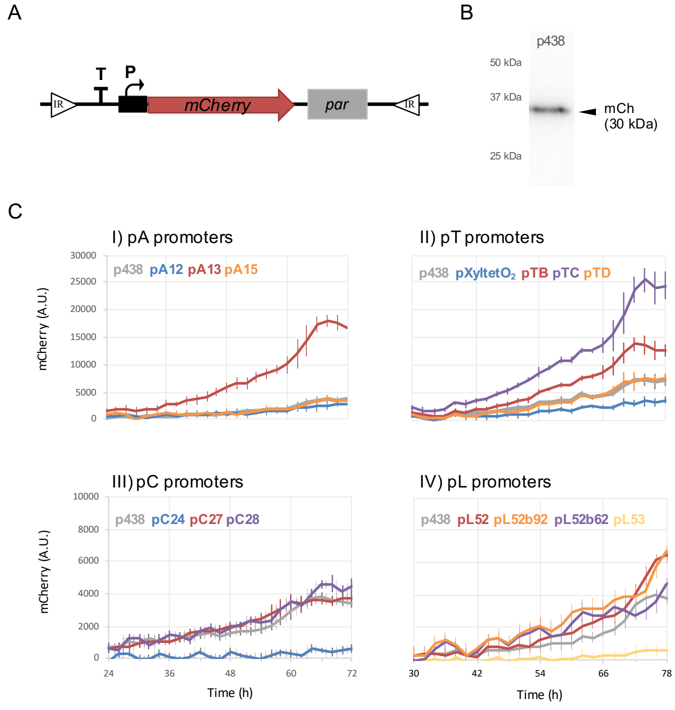

Suppl. Fig. 2. Activity of new engineered synthetic promoters for *M. pneumoniae*. **a** Schematic representation of the MTn*par* platform used to test the promoter candidates. It contains a transcriptional terminator to insulate the promoter activity and an mCherry reporter gene. Strains generated with this

MTn*par* were selected with puromycin. **b** Western blot result showing the expression of the mCherry reporter in the late exponential phase of growth for the positive control strain with the p438 constitutive promoter. **c** Kinetics of mCherry expression using different engineered synthetic promoters with AraR-operators (I); TetR-operators (II); CI-operators (III); and LacI-operators (IV). Graphs showing the corresponding mCherry kinetics (fluorescence in arbitrary units). All cases include the positive control with promoter p438 (grey lines). Mean values from three bio-replicates. Error bars indicate standard deviation. Source data are provided as a Source Data file.

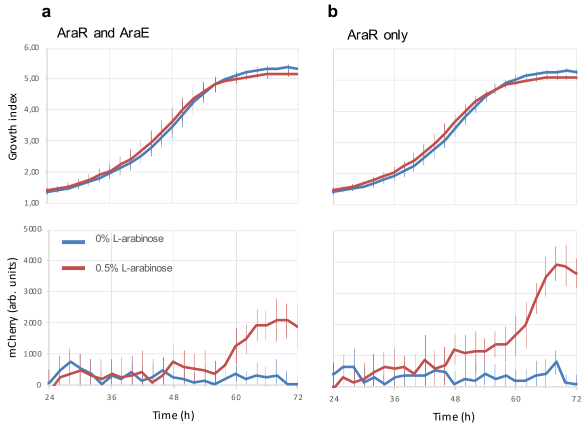

Suppl. Fig. 3. Performance of the full AraR system in *M. pneumoniae* in a strain with (**a**) or without (**b**) the AraE permease. In both cases, we show L-arabinose induction of the inducible promoter pA13. The upper graphs show synchronised growth (growth index as the ratio Abs430nm/Abs560nm) between uninduced (blue) or 0.5% L-arabinose induced (red) cultures, and the lower graphs show the corresponding mCherry kinetics (fluorescence in arbitrary units). Mean values from three bio-replicates. Error bars indicate standard deviation. Source data is provided in a Source Data file.

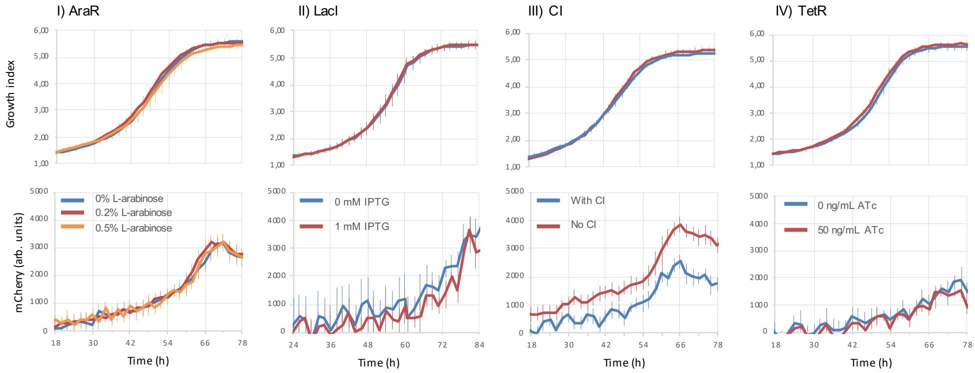

Suppl. Fig. 4. Kinetics of mCherry expression driven by the promoter p438 in strains with constitutive expression of AraR (I), LacI (II), CI (III) or TetR (IV) repressors. The upper graphs show synchronised growth between samples (growth index corresponding to the ratio Abs430nm/Abs560nm) in the absence of inducer (blue) or with the corresponding inducer for each repressor (red and orange). The lower graphs show the corresponding mCherry kinetics (fluorescence in arbitrary units). The expression of the different repressors did not affect the mCherry expression, as expected when it is driven by a constitutive promoter, like the p438 used in these analysis (See suppl. Fig. 2A for the schematic of the mCherry expression cassette). Only a difference appears in comparing mCherry expression between the strains with or without

the CI protein (III). In this particular case, the first strain without CI has a single MTn*par* with the mCherry cassette. At the same time, the second strain also has a second MTn*cat* with the *cI* expression cassette and, most likely, the lower expression of mCherry is due to the increased metabolic load in the cell (the transformation of the first strain with an empty MTn*cat* would have been a better control). Mean values from three bio-replicates. Error bars indicate standard deviation. Source data are provided as a Source Data file.

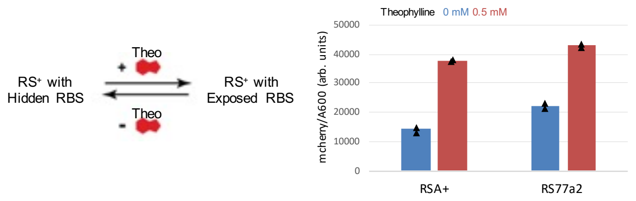

Suppl. Fig. 5. Analysis of synthetic theophylline-riboswitches in *M. pneumoniae*. a Mode of action of a theophylline-activation riboswitch (RS^+). The theophylline (Theo) binding produces a change in the riboswitch structure that exposes a hidden RBS. The mCherry expression depending on the theophylline concentration measured by Fluorimetry in the late exponential phase for the two best riboswitch candidates RSA^+ and RS77a2. Graph shows bars with mean values and black triangles indicate values from each of the two bio-replicates analysed. Source data are provided as a Source Data file.

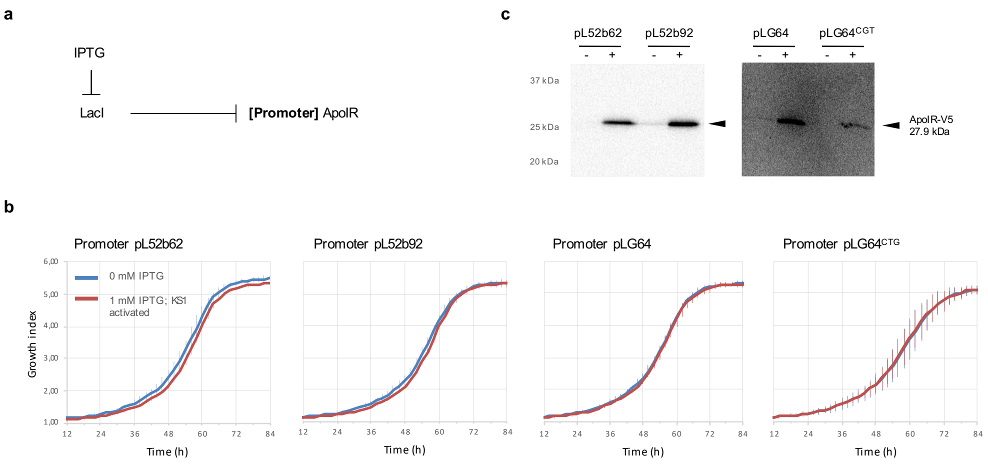

Suppl. Fig. 6. Study of a kill-switches based on the restriction enzyme ApoRI engineered with different IPTG-inducible promoters. **a** Schematic representation of the IPTG-inducible kill-switch with ApoRI. Blunt arrows indicate repression. **b** Growth kinetics of four polyclonal strains with this kill-switch engineered with the IPTG-inducible promoter pL52b62, pL52b92, pLG64 or the same pLG64 combined with the *apoRI* gene with the weak starting codon CTG ($pLG64^{CGT}$). The graphs show growth kinetics (growth index corresponding to the ratio Abs430nm/Abs560nm) when the kill-switch is uninduced (0 mM IPTG, blue lines) or IPTG-induced (1 mM IPTG, kill-switch activated, red lines). Mean values from three bio-replicates. Error bars indicate standard deviation. **c** Western blot results showing ApoRI (with C-terminal V5-tag) expression in the four polyclonal strains with the kill-switch with different inducible promoters. Expression was analysed using 10 μg of total protein extract from pre-induced (-) and 1 mM IPTG-induced (+) samples. Source data are provided as a Source Data file.

Similar kill-switches with other restriction enzymes revealed equivalent results. We tested the enzymes SspI and AseI, which produce blunt and cohesive cuts in the DNA, respectively. Here we show results with ApoRI that is a restriction enzyme that also produce a blunt cut in the DNA but its optimal temperature is

30ºC and at 37ºC the activity is reduced to 50%, at least *in vitro*, and we expected this enzyme to be less toxic among the three tested.

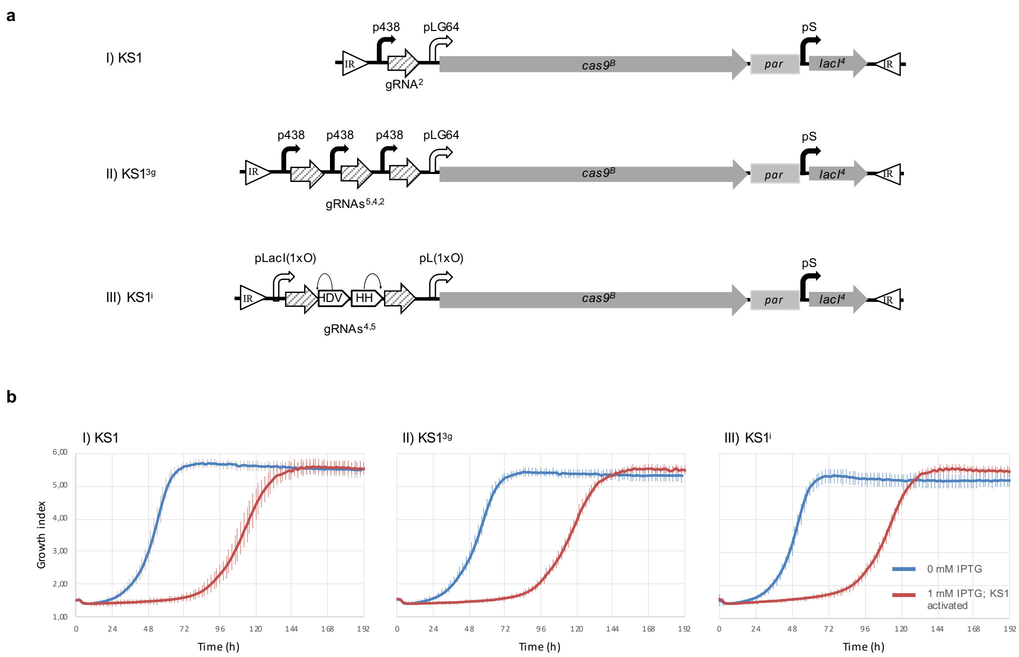

Suppl. Fig. 7. Variations of the kill-switch KS1. We analysed two variations of the kill-switch KS1. On the one hand, we added multiple constitutive gRNA (with different targets in the genome) in the kill-switch (II, KS1^{3g}). On the other hand, we re-engineered the kill-switch so that the *cas9* gene is inducible and a pair of gRNAs (III, KS1^{i}). For that, we included two different gRNAs under a new synthetic promoter with a single LacI-operator box and two self-cleaving ribozymes (HDV and HH[1]) inserted between the two gRNAs, so that the transcript would be broken into three pieces, releasing the two gRNAs. The IPTG-inducible promoter for *cas9* expression is also modified to contain a single LacI-operator 'upside-down'. What is most important is the 401 bp distance between the LacI-operator boxes included in the two promoters. The LacI is thus able to bind the two operator boxes (given the DNA-looping and bending that will bring the two boxes together) and allows the repression of the two promoters simultaneously. **a** Schematic representation of relevant DNA segments of the MTn*par* used to generate polyclonal strains with the three variations of the kill-switch studied: KS1 (I), KS1^{3g} (II) and KS1^{i} (III). White triangles for the inverted (IR); Bend-arrows for constitutive (black) and IPTG-inducible (white) promoters; Arrows show genes (grey), gRNAs (stripped; gRNA2 has eight targets in the genome of *M. pneumoniae*, gRNA4 has a single target in the *sigA* gene, and gRNA5 has a single target in the *ligA* gene), and ribozymes (white); the small curvy arrows indicate the cleavage-site for each ribozyme; *par*, selection cassette. **b** Growth kinetics of the three polyclonal strains with the three versions of the kill-switch. Graphs show growth kinetics (growth index as the ratio Abs430nm/Abs560nm) when the kill-switch is uninduced (0 mM IPTG, blue lines) or IPTG-induced (kill-switch activated with 1 mM IPTG, red lines). Mean values from three bio-replicates. Error bars indicate standard deviation. Source data are provided as a Source Data file.

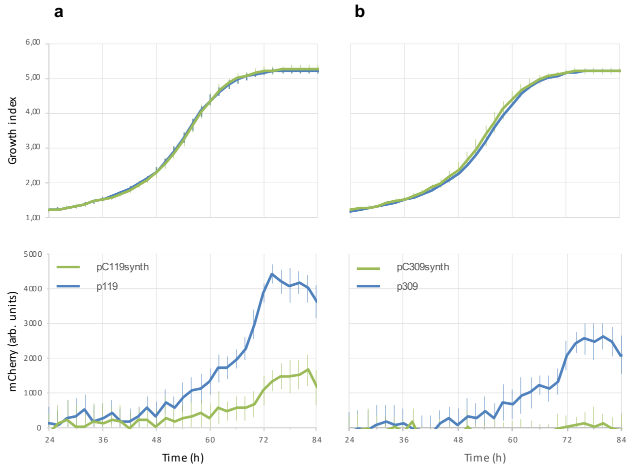

Suppl. Fig. 8. Engineering *M. pneumoniae* promoters to accommodate CI-operator sites. Comparison of mCherry expression using a wild-type *mycoplasma* promoter or the inducible version engineered with CI-operators. **a** Promoter p119 (blue) and the inducible version pC119synth (green). **b** Promoter p309 (blue) and the inducible version pC309synth (green). The upper graphs show synchronised growth (growth index as the ratio Abs430nm/Abs560nm) between uninduced (blue) or 0.5% L-arabinose induced (red) cultures, and the lower graphs show the corresponding mCherry kinetics (fluorescence in arbitrary units). Mean values from three bio-replicates. Error bars indicate standard deviation. Source data are provided as a Source Data file.

In all cases analysed, the inclusion of two CI-operator boxes in close proximity to the TATA box of the promoter reduces the its strength quite significantly, most likely due to the rich GC content of the operators. Modifying the strength of a promoter for an essential element can impact the growth rate of the strain, although the leakiness once repressed might still allow some viability of the cell and, most likely, produce a strong selection pressure for escape mutants. Nonetheless, this would be an interesting application for the the CI included in the kill-switch TKS2.

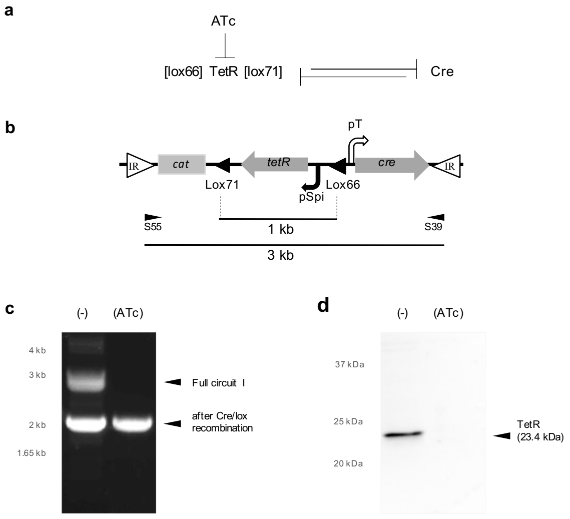

Suppl. Fig. 9. Design and characterisation of a Cre positive feedback loop (circuit I). **a** Schematic representation of the circuit I. Blunt arrows indicate repression. **b** Schematic representation of relevant DNA segments of the MTn*cat* with circuit I used to generate a polyclonal strain (chloramphenicol used for selection). White triangles for the inverted repeats (IR); Bend-arrows for a constitutive (black) and ATc-inducible (white) promoters; Grey arrows show genes; Black triangles indicate the position and orientation

of the lox sites (lox66 and lox71); *cat*, selection cassette. **c** DNA gel showing the results from the PCR analysis of Cre/lox recombination in circuit I. Genomic DNA from uninduced (-) and ATc-induced (ATc) samples tested with the primers pair S39 and S55. PCR product size expected for the full circuit I, before Cre/lox recombination, and the distance between lox sites are indicated in (b) between the small head arrows S39 and S55, showing the position of the primers pair used. **d** Western blot results showing TetR expression in a polyclonal strain with the circuit I. Protein expression was analysed using 25 µg of total protein extracts from pre-induced (-) and 50 ng/mL ATc-induced (ATc) samples. DNA gel and western blot results are representative of two independent experiments in each case.

This circuit shows a clear leakage of Cre before induction, although TetR is not completely lost until full induction with ATc. Nevertheless, Cre expression had to be optimised to reduce to the minimum its basal expression, as shown in the following example with circuit II.

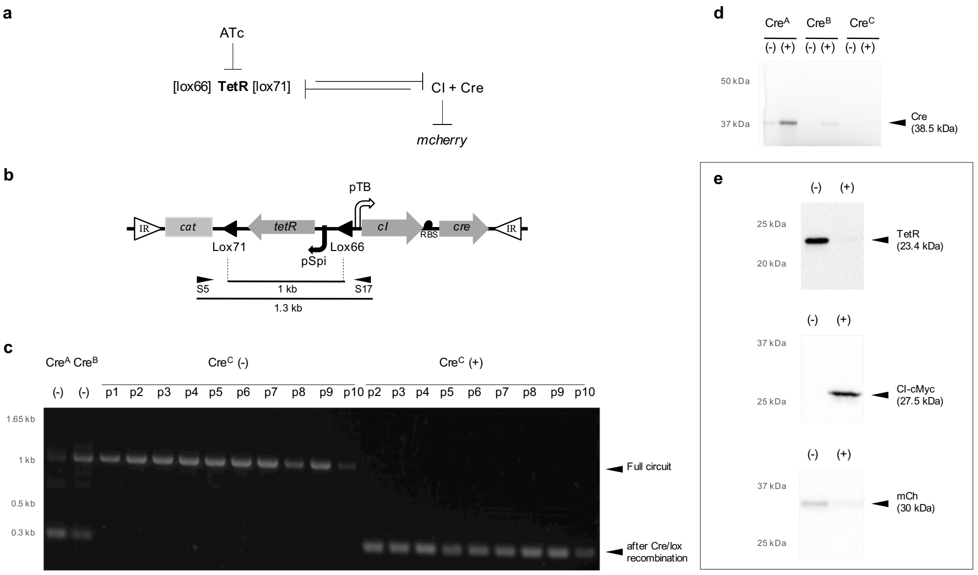

Suppl. Fig. 10. Design and optimisation of an irreversible ATc-inducible positive feedback loop that triggers expression of CI and Cre (circuit II). **a** Schematic representation of circuit II. Blunt arrows indicate repression. **b** Schematic representation of relevant DNA segments of the MTn*cat* with circuit II used to generate a polyclonal strain (chloramphenicol used for selection). White triangles for the inverted repeats (IR); Bend-arrows for constitutive (black) and ATc-inducible (white) promoters. Arrows for genes (grey); Black semicircle shows the RBSs; Black triangles indicate the position and orientation of the lox sites (lox66 and lox71); *cat*, selection cassette. **c** DNA gel showing the results from the PCR analysis of Cre/lox recombination in circuit II. In this case, we show results comparing three strains carrying circuit II but with a the *cre* gene (*cre*^{A}), with *cre* gene with the initiation codon mutated to CTG (*cre*^{B}), and with the *cre* gene with the initiation codon mutated to CTG and adding nine suboptimal codons among the twenty initial codons (*cre*^{C}). Genomic DNA samples from uninduced (-) and ATc-induced (ATc) induced samples tested with the primers pair S39 and S55 (small head arrows shown in B). PCR product size expected for the full circuit II, before Cre/lox recombination, and the distance between lox sites are also indicated in (b). Passage of the strain indicated when relevant (p1 to p10). **d** Western blot results showing Cre expression in the three versions of circuit II studied with *cre*^{A}, *cre*^{B} and *cre*^{C}. Cre expression was analysed using 25 µg of total protein extracts from pre-induced (-) and 50 ng/mL ATc-induced (+) samples. **e** Western blot results showing TetR, CI and Cas9 expression in a strain with circuit II optimised with *cre*^{C}. Expression of the proteins was analysed using 10 µg of total protein extracts from pre-induced (-) and 50 ng/mL ATc-induced (+) samples. DNA gel and western blot results are representative of two independent experiments in each case.

We optimised the circuit II by reducing drastically the basal expression of Cre. For that, we not only mutated the initiation codon of the *cre* gene to the less efficient CTG codon but also added up to nine suboptimal codons among the initial twenty codons (considering data for *M. pneumoniae* [gbbct] in the Codon usage https://www.kazusa.or.jp/codon/cgi-bin/showcodon.cgi?species=2104&aa=4&style=N"). In that case, circuit II with *creC* gene is very stable among sequential passaging of 48h (up to 10 passages tested).

With this circuit II, we also evaluated the activity of CI repressor as a potential mechanism to silence essential genes in *Mycoplasma*. For that, we included an *mcherry* gene expressed via pC27 promoter. Western blot results show good expression of mCherry in uninduced samples and a considerably reduction of the expression after ATc-induction of CI in circuit II. Nevertheless, the limited expression observed for mCherry is an indication that the silencing could be leaky and its impact should be studied for the specific case.

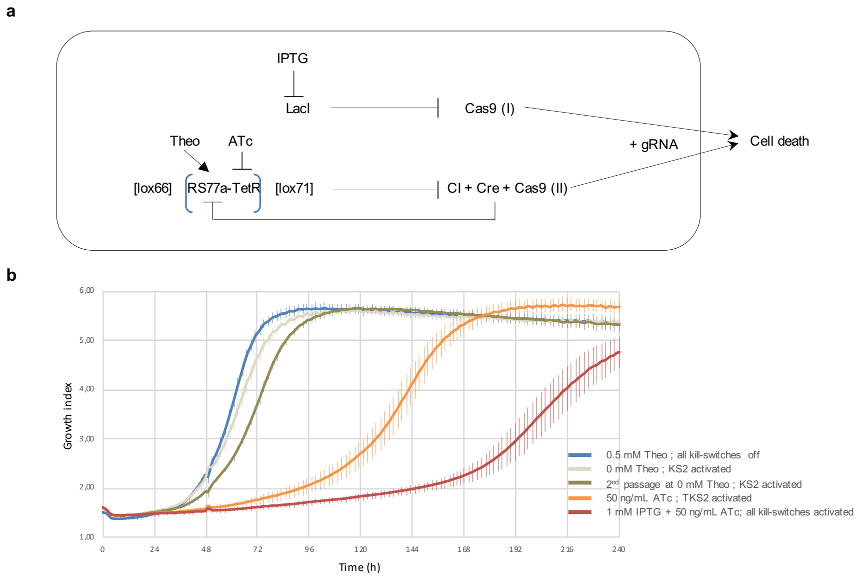

Suppl. Fig. 11. Analysis of the growth kinetics of a polyclonal strain with the combined biosafety system KS1 and TKS2. To generate a strain with the combined biosafety system KS1 and TKS2, we retransformed with a minitrasposon with the circuit TKS2 the clonal strain C5, which already has the kill-switch KS1 and a functional gRNA. Notice that the resulting strain is only polyclonal for the second MTn inserted. **a** Schematic representation of a cell with the two kill-switches KS1 (I) and TKS2 (II). The expression of Cas9 from either kill-switch with the constitutive gRNA will produce fatal toxicity that kills the cell. **b** Growth kinetics (growth index as the ratio Abs430nm/Abs560nm) of a polyclonal strain with KS1 and TKS2 at different combination of inducers. The graph compares growth of the strain in the permissive growth conditions in theophylline-medium (0.5 mM Theophylline, both circuits inactive, blue line), and the first and second passages in theophylline-free medium that produce slow activation of only TKS2 (0 mM Theophylline, light and dark brown lines, respectively). We also compare growth when only TKS2 is activated in ATc-medium (50 ng/mL ATc, orange line) and when both circuits are activated simultaneously in ATc and IPTG medium (1 mM IPTG and 50 ng/mL ATc, red line). Mean values from three bio-replicates. Error bars indicate standard deviation. Source data are provided as a Source Data file.

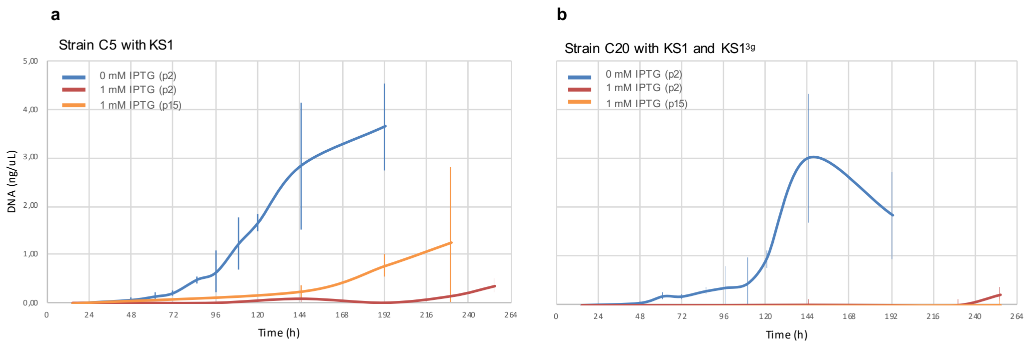

Suppl. Fig. 12. DNA growth kinetics of strains C5 (**a**) and C20 (**b**). We compare the growth of strains C5 and C20 (with one and two copies of the kill-switch KS1, respectively) when uninduced at the early passage p2 (0 mM IPTG, blue lines) or IPTG-induced that triggers the kill-switch at the passage 2 (1 mM IPTG; p2, red lines) or passage 15 (1 mM IPTG; p15, orange line). Mean values from three bio-replicates. Error bars indicate standard deviation. Source data are provided as a Source Data file.

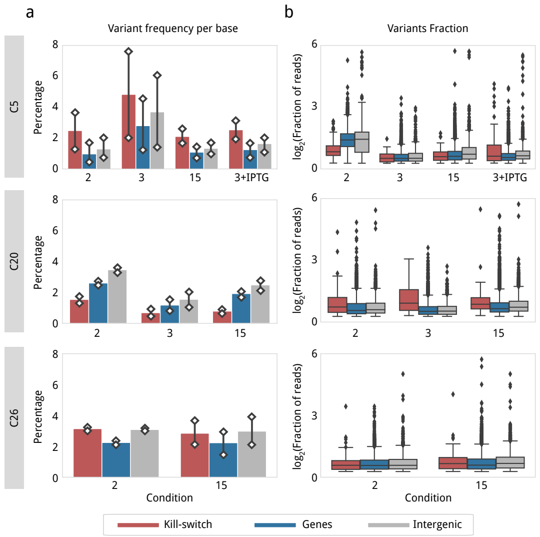

Suppl. Fig. 13. Graphical exploration of variants reported in C5 samples (top raw), C20 samples (middle raw) and C26 (bottom raw). Variants corresponding to the kill-switch(es) (red), *Mycoplasma pneumoniae* genes (blue) or intergenic regions (grey) for each condition tested are shown in the x-axis of each representation. Notice 3+IPTG corresponds to the sample induced with IPTG in passage 3 for the C5 samples. **a** bar plots showing total variants normalised by the total reads within the annotation (Percentage, Y-axis). The bar top is centered in the mean value for n=2 independent samples while the black line represents the standard deviation. Independent values are represented by diamonds associated with each condition and library. **b** box plots showing the distribution of the fraction of reads having an alternative sequence variation (as log_2, Y-axis). Each box is defined by the first quartile (Q1; lower bound) to the third (Q3; upper bound) with the median in between (*i.e.* second quartile, or Q2). Given an interquartile range (IQR; *i.e.* range between Q3-Q1), whiskers are defined by extending them to the data points that are less than Q3 + 1.5(IQR) and greater than Q1 - 1.5(IQR) for upper and lower whiskers, respectively. Data points out of these limits are individually represented by black diamonds. Source data have been deposited in the ArrayExpress database at EMBL-EBI, under accession number E-MTAB-10981. The complete process to generate this figure can be replicated by running the code available at GitHub, as indicated in the Code availability section.

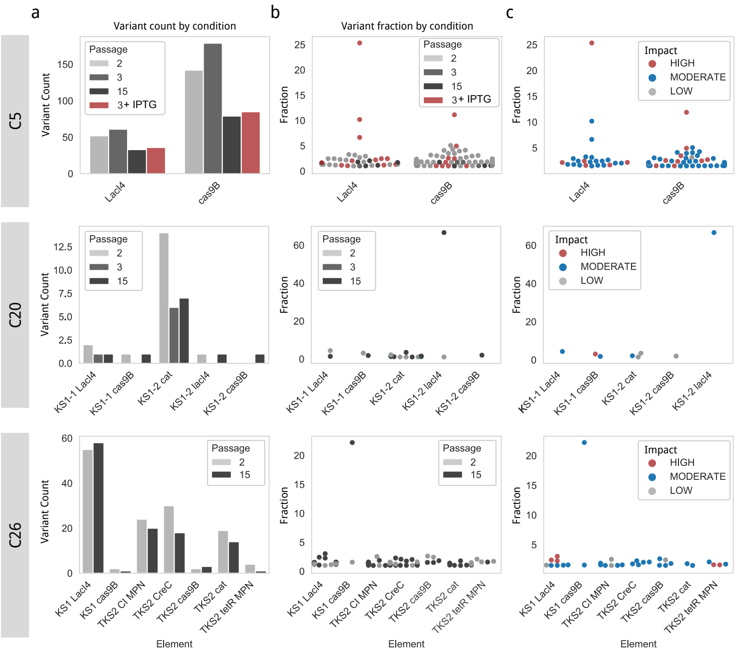

Suppl. Fig. 14. Graphical exploration of variants reported within the kill-switch(es) in C5 samples (**top**), C20 samples (**middle**) and C26 (**bottom**). Graphs show the relevant genes from the kill-switch(es) present in the strains (X-axis). Left column, bar plots representing the total variants counted (Y-axis) in the different genes from the kill-switch(es) for each condition tested (passages 2, 3 and 15 in grey and 3+IPTG in red). Middle column, Swarm plot of the fraction of reads presenting an alternative sequence or variant (as $\log_2$, Y-axis), coloured by the passage they are found in. Right column, Same swarm plot as previous but coloured by the impact of the variant in the circuit. Source data have been deposited in the ArrayExpress database at EMBL-EBI, under accession number E-MTAB-10981.

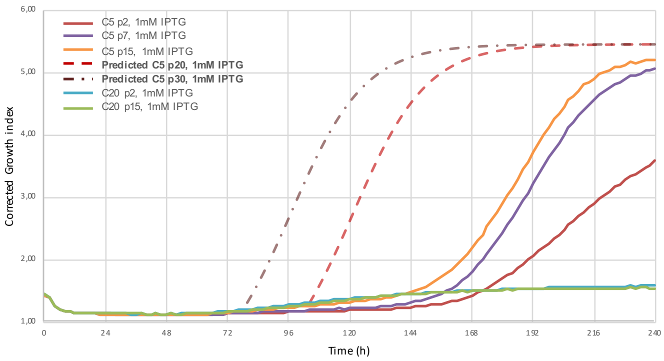

Suppl. Fig. 15. Simulation of the growth of *M. pneumoniae* clonal strain C5 and C20. Prediction for strain C5 in the presence of 1 mM IPTG at passages 20 and 30 (dashed red and brown lines, respectively), compared to the experimental data of the strain at passages 2 (red), 7 (yellow) and 15 (blue). In the same graph, C20 strain passages 2 (turquoise) and 15 (green), overlapping between each other. Growth data is shown in terms of background-corrected growth index (CorrectedAbs430/CorrectedAbs560; Cabs430/Cabs560). Source data are provided as a Source Data file.

Supplementary Tables

Suppl. Table 1. DNA sequences of all elements used in this work. The excel file includes in separate sheets the following information: **a** Results of the *in silico* prediction of promoters for *M. pneumoniae*; **b** DNA sequences of all the parts (including constitutive and synthetic inducible promoters, operator boxes, terminators, RBSs, leader peptides, ORFs, Tag peptides, gRNAs, ribozymes, riboswitches, lox sites and vectors); **c** DNA sequences of the tools design for *M. pneumoniae*; **d** DNA sequences of kill-switches and circuits engineered for *M. pneumoniae*; **e** List of relevant primers; **f** List of MTn vectors and **g** List of strains used in this work.

Suppl. Table 2. Results for the variant calling analysis performed one samples of the strains C5, C20 and C26 at different passages. The excel file includes the results for each sample in separate sheets. Columns correspond to sample identifier (SAMPLE); passage (2,3 or 15; PASS); genome loci where the variant is found (base pair position; POS); estimate of the probability that there is a polymorphism at the loci described by the record (QUAL); total number of reads obtained for the loci (TOT); total number of reads matching the reference (REFN); number of reads presenting a variant (ALTN); frequency of the variant considering the total reads for the loci (FRAC); reference sequence in the genome (REF); variant sequence (ALT); potential effect of the variant as given by snpeff[2](EFF); degree of impact of the variant (IMPACT), which is classified as "LOW" (synonymous mutations), "MODERATE" (missense), "HIGH" (non-synonymous mutations, start or stop loss), or MODIFIER (variant found in an intergenic region); gene affected (AFF, in case the mutation is found intergenic, it is reported the closest downstream gene); mutation or variant (MUT, in nucleotides when found intergenic); type of annotation assigned to the loci (ANN_TYPE), grouped as intergenic, gene, or cassette (covering the principal elements in the cassette).

Observation qualities. Freebayes[3] estimates observation quality using several simple heuristics based on manipulations of the phred-scaled base qualities:

- For single-base observations, *mismatches* and *reference observations*: the un-adjusted base quality provided in the BAM alignment record.
- For *insertions*: the mean quality of the bases inside of the putatively inserted sequence.
- For *deletions*: the mean quality of the bases flanking the putatively deleted sequence.
- For *haplotypes*: the mean quality of allele observations within the haplotype.

By default, both base and mapping quality are into the reported site quality (QUAL in the VCF) and genotype quality (GQ, when supplying --genotype-qualities). This integration is driven by the "Effective Base Depth" metric first developed in snpTools, which scales observation quality by mapping quality:

P(Obs|Genotype) ~ P(MappedCorrectly(Obs))P(SequencedCorrectly(Obs)).

Set --standard-gls to use the model described in the freebayes preprint.

Suppl. Table 3. List of highly represented mutations by sequencing after inducing the kill-switch KS1 in the strain C5 with IPTG at passage 3 (sample p3IPTG). Columns are shared with Suppl. Table 1. In this case we include a column TOP, which displays 1 for those positions found significant, 0 otherwise.

Suppl. Table 4. Growth impact of single gene disruptions in *M. pneumoniae*. Columns show: **a** gene disrupted; **b** Prediction of growth when disruption; and **c** % of growth change compared to WT (Strain M129).

Supplementary information

Suppl. Info. 1. Background correction of the long growth kinetics

Volume reduction over time, due to evaporation, observed in long growth kinetics affects mainly Abs^{560} detection, while the effect on Abs^{430} is minimal. This is observed in the negative control of growth included in the experiments, where we monitored growth index from a sample with SP-4 medium in absence of the bacterium (Suppl. File 2). Linear regression was performed on SP-4 data through the R command *lm* for both Abs^{430} and Abs5^{60}, to capture the different entity of the effect of the volume reduction on the detection. Assuming both Abs^{430} and Abs^{560}, in absence of the microorganism, should not change over time, the slope of the line obtained by linear regression was subtracted from the point-by-point mean of the original data, provided in triplicates. Slopes of the lines correspond to values of 1^{-08} for Abs^{430} and -2^{-07} for Abs^{560}. The rates of absorbances CAbs^{430}/CAbs^{560} for SP-4 were recomputed with the background-corrected absorbances and standard deviations were recalculated.

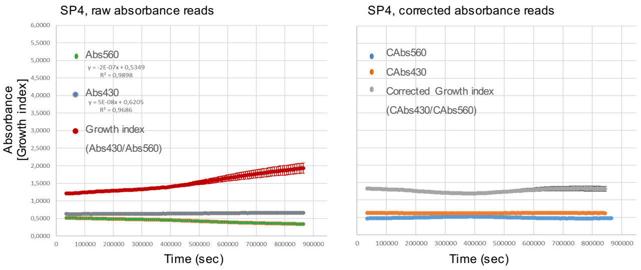

Fig. A. Background correction of SP4 absorbance reads at 430 nm and 560 nm. Left graph, evolution of the absorbance reads of empty SP4, with the corresponding linear regressions for Abs430 and Abs560. The graph also shows the resulting growth index (Abs430/Abs560). Right graph, corrected absorbance reads CAbs430 and Cabs560, and the resulting corrected growth index (CAbs430/CAbs560). Source data are provided as a Source Data file.

The difference between Abs^{430}/Abs^{560} and CAbs^{430}/CAbs^{560} was used to correct the data for all the strain types, for each condition and time point. See below all the curves with the background correction, compared to the original data (curves shown in Figure 4).

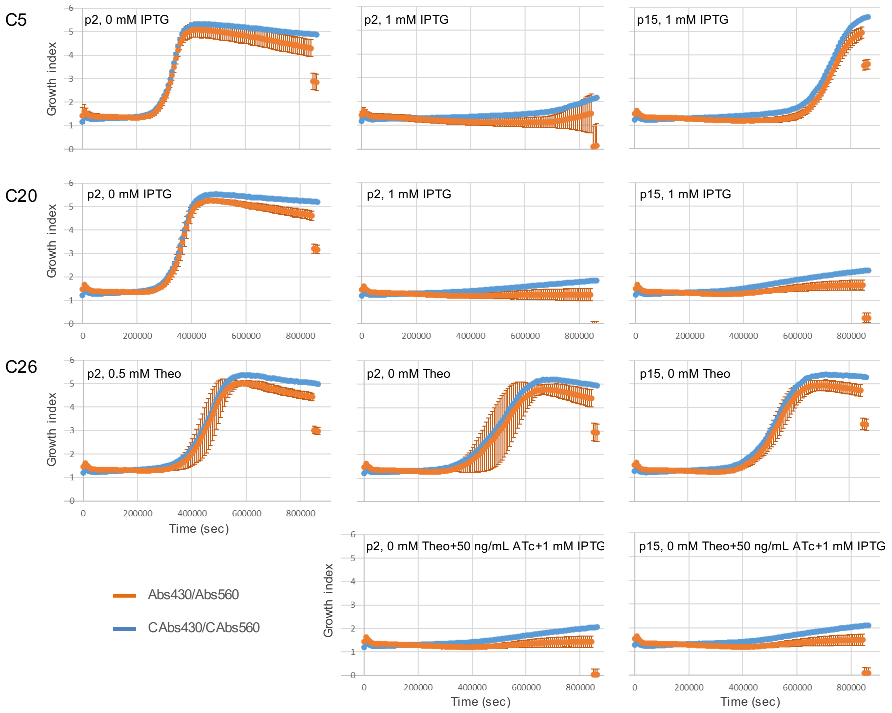

Fig. B. Background correction of the long growth kinetics. Growth kinetics corresponds to growth index as the ratio Abs430nm/Abs560nm (orange lines) or the corrected growth index as the ratio of corrected absorbance reads CAbs430nm/CAbs560nm (blues lines) of the clonal strains C5, C20 and C26 analysed at an early (p2) and late passage (p15) at different induction conditions. Source data are provided as a Source Data file.

Suppl. Info. 2. Variant-calling analysis. See the ksanalysis notebook with analysis details below.

References

1. Gao, Y. & Zhao, Y. Self-processing of ribozyme-flanked RNAs into guide RNAs in vitro and in vivo for CRISPR-mediated genome editing. *J. Integr. Plant Biol.* **56**, 343–349 (2014).
2. Cingolani, P. *et al.* A program for annotating and predicting the effects of single nucleotide polymorphisms, SnpEff. *Fly (Austin).* **6**, 80–92 (2012). [ref. 102 in main manuscript].
3. Garrison, E. & Marth, G. Haplotype-based variant detection from short-read sequencing. *arXiv:1207.3907v2 [q-bio.GN]* (2012). [ref. 101 in main manuscript].

Content list of supplementary SourceData&analysis_Mycotoolscircuits.zup file

- SourceData&analysis_Fluorimetries.xlsx with data corresponding to figure 1 and Suppl. Fig. 5
- SourceData&analysis_TimeCourses.xlsx with data corresponding to figures 1, 2 and 4; and Suppl. Fig. 2, 3, 6, 7, 11, 15, A and B.
- SourceData&analysis_E.F.xlsx with data corresponding to figure 4
- SourceData&analysis_qPCR.xlsx with data corresponding to Suppl. Fig. 12

ksanalysis

June 15, 2021

1 Variant calling analysis Killswitch circuit

Defining variants (SNPs, insertions and deletions) for a set of HiSeq samples exploring the variability in populations of *M. pneumoniae* transformed with a killswitch cassette (C5 samples) or two (C20 samples); sequencing performed at different selection passages (2, 3, 15; 2 replicates in each condition and cassette) and a pair of replicates at passage 3 were the cassette was induced by IPTG.

Authors: Samuel Miravet-Verde and Alicia Broto

Last update: 14/06/2021

[662]:
```
%load_ext autoreload
%autoreload 2
%matplotlib widget

import sys, os
import glob
import pandas as pd
import numpy as np
import seaborn as sns
import matplotlib.pyplot as plt
import scripts
from collections import Counter
from Bio import SeqIO
from Bio.SeqFeature import SeqFeature, FeatureLocation

sns.set_style("whitegrid", {'axes.grid' : False})
```

```
The autoreload extension is already loaded. To reload it, use:
  %reload_ext autoreload
```

[663]:
```
# General info about the genome references and the location of the cassettes in␣
↪the genomes
seq_record5 = SeqIO.read('./data/C5_refSeq.gb', "genbank")
seq_record20 = SeqIO.read('./data/C20_refSeq.gb', "genbank")
genome_C5 = str(seq_record5.seq)
genome_C20 = str(seq_record5.seq)
```

```
cass_par = [565510, 572124] # Present in C5 and C20
cass_3b = [780850, 787445]  # Only in C20

# Annotation information to work faster with dataframes
ncbi = pd.read_csv('./data/mpn_annotation.csv', sep='\t', header=None) # Genome␣
↪annotation
ncbi.columns = ['gene', 'start', 'end', 'strand']
ncbi.set_index('gene', inplace=True)
gold = pd.read_csv('./data/goldsets.csv', sep='\t')  # Gold set

posN = []
posE = []
for gene, cat in zip(gold['gene'], gold['class']):
    if cat=='E':
        posE += range(ncbi.loc[gene][0], ncbi.loc[gene][1]+1)
    else:
        posN += range(ncbi.loc[gene][0], ncbi.loc[gene][1]+1)

# Define the different set of positions in the genome were we will perform the␣
↪analysis
posN = set(posN)
posE = set(posE)
cas1 = set(range(cass_par[0], cass_par[1]+1))
cas2 = set(range(cass_3b[0], cass_3b[1]+1))
cas12 = cas1.union(cas2)
geno = set(range(1, 816395))

ingene = []
for st, en in zip(ncbi['start'], ncbi['end']):
    ingene+=list(range(st, en))
ingene = set(ingene)
outgene = geno.difference(ingene)
```

1.1 1. Map variations

We will be using Snippy for the first steps related to sequence mapping. To run it in parallel for all the samples we create a input file:

[664]:
```
### 1. Make a multifile to map with snippy-multi.
files = glob.glob('../data/C[5|20]*/*_R1_*') # The gre expression refers to the␣
↪raw sequencing files located in data (this has to point to the directory␣
↪where fastq files are stored)

fo1 = open('./tmp_files/snippy_input1.txt', 'w')
fo2 = open('./tmp_files/snippy_input2.txt', 'w')
```

```
for f in files:
    ide = f.split('/')[2:]
    if 'C5' in f:
        ide = ide[0].replace('C5', '')+'_'+'_'.join(ide[1].split('_')[:-3])
        fo1.write('{}\t{}\t{}\n'.format(ide, f, f.replace('_R1_', '_R2_')))
    else:
        ide = ide[0].replace('C20', '')+'_'+'_'.join(ide[1].split('_')[:-3])
        fo2.write('{}\t{}\t{}\n'.format(ide, f, f.replace('_R1_', '_R2_')))
fo1.close()
fo2.close()
files
```

[664]:
```
['../data/C20p3/B_S1_L001_R1_001.fastq.gz',
 '../data/C20p3/B_S2_L001_R1_001.fastq.gz',
 '../data/C5p3/D_S2_L001_R1_001.fastq.gz',
 '../data/C5p3/D_S3_L001_R1_001.fastq.gz',
 '../data/C5p3IPTG/A_S1_L001_R1_001.fastq.gz',
 '../data/C5p3IPTG/A_S1b_L001_R1_001.fastq.gz',
 '../data/C20p15/7_S3_L001_R1_001.fastq.gz',
 '../data/C20p15/7_S5_L001_R1_001.fastq.gz',
 '../data/C5p15/F_S5_L001_R1_001.fastq.gz',
 '../data/C5p15/F_S1_L001_R1_001.fastq.gz',
 '../data/C5p2/2_S2_L001_R1_001.fastq.gz',
 '../data/C5p2/2_S3_L001_R1_001.fastq.gz',
 '../data/C5p2/2_S1_L001_R1_001.fastq.gz',
 '../data/C20p2/6_S2_L001_R1_001.fastq.gz',
 '../data/C20p2/6_S4_L001_R1_001.fastq.gz']
```

Now run the command in the snippy environment:

```
conda activate snippy
snippy-multi ./data/snippy_input.txt --ref ./data/C5_refSeq.gb --cpus 4 --report >  ./snippy_ru
sh ./snippy_run.sh
```

Run freebayes in a permissive manner. This command will create a directory with the name of the sample were all the files will be stored. This is expected to be run in the terminal copy-pasting the printed command. It can also be run changing the print to `os.system` to execute it for each file:

[5]:
```
# Listed samples in order
order1 = ['p2_2_S1','p2_2_S2','p2_2_S3','p3_D_S2',␣
↪'p3_D_S3','p15_F_S1','p15_F_S5', 'p3IPTG_A_S1', 'p3IPTG_A_S1b']
order2 = ['p2_6_S2','p2_6_S4','p3_B_S1','p3_B_S2','p15_7_S3','p15_7_S5']
```

[6]:
```
# freebayes-parallel reference/ref.txt 4 -p 2 -P 0 -C 2 -F 0.05 --min-coverage␣
↪10 --min-repeat-entropy 1.0 -q 13 -m 60 --strict-vcf   -f reference/ref.fa␣
↪snps.bam > snps.raw.vcf
for fil in glob.glob('../p*/*.bam'):
    # again this has to point to directories were snipy output is stored
```

```
    ide = fil.split('/')[1]
    #print('freebayes-parallel {}/reference/ref.txt 4 -p 1 -P 0 -C 1 -F 0.01 -q␣
↪13 -m 60 --strict-vcf -f {}/reference/ref.fa {} > filter10/{}.raw.vcf'.
↪format(ide, ide, fil, ide))
    #print('freebayes-parallel {}/reference/ref.txt 4 -p 1 -P 0 -C 2 -F 0.05 -q␣
↪13 -m 60 --strict-vcf -f {}/reference/ref.fa {} > filter50/{}.raw.vcf'.
↪format(ide, ide, fil, ide))
    if ide in order1:
        print('freebayes-parallel {}/reference/ref.txt 4 -p 1 -P 0 -C 1 -F 0.
↪001 -q 13 -m 60 --strict-vcf -f {}/reference/ref.fa {} > filter1/{}.raw.vcf'.
↪format(ide, ide, fil, ide))
    else:
        print('freebayes-parallel {}/reference/ref.txt 4 -p 1 -P 0 -C 1 -F 0.
↪001 -q 13 -m 60 --strict-vcf -f {}/reference/ref.fa {} > filter2/{}.raw.vcf'.
↪format(ide, ide, fil, ide))
```

```
freebayes-parallel p3_D_S2/reference/ref.txt 4 -p 1 -P 0 -C 1 -F 0.001 -q 13 -m
60 --strict-vcf -f p3_D_S2/reference/ref.fa ../p3_D_S2/snps.bam >
filter1/p3_D_S2.raw.vcf
freebayes-parallel p2_6_S2/reference/ref.txt 4 -p 1 -P 0 -C 1 -F 0.001 -q 13 -m
60 --strict-vcf -f p2_6_S2/reference/ref.fa ../p2_6_S2/snps.bam >
filter2/p2_6_S2.raw.vcf
freebayes-parallel p2_2_S1/reference/ref.txt 4 -p 1 -P 0 -C 1 -F 0.001 -q 13 -m
60 --strict-vcf -f p2_2_S1/reference/ref.fa ../p2_2_S1/snps.bam >
filter1/p2_2_S1.raw.vcf
freebayes-parallel p15_7_S5/reference/ref.txt 4 -p 1 -P 0 -C 1 -F 0.001 -q 13 -m
60 --strict-vcf -f p15_7_S5/reference/ref.fa ../p15_7_S5/snps.bam >
filter2/p15_7_S5.raw.vcf
freebayes-parallel p3IPTG_A_S1/reference/ref.txt 4 -p 1 -P 0 -C 1 -F 0.001 -q 13
-m 60 --strict-vcf -f p3IPTG_A_S1/reference/ref.fa ../p3IPTG_A_S1/snps.bam >
filter1/p3IPTG_A_S1.raw.vcf
freebayes-parallel p3_D_S3/reference/ref.txt 4 -p 1 -P 0 -C 1 -F 0.001 -q 13 -m
60 --strict-vcf -f p3_D_S3/reference/ref.fa ../p3_D_S3/snps.bam >
filter1/p3_D_S3.raw.vcf
freebayes-parallel p3_B_S1/reference/ref.txt 4 -p 1 -P 0 -C 1 -F 0.001 -q 13 -m
60 --strict-vcf -f p3_B_S1/reference/ref.fa ../p3_B_S1/snps.bam >
filter2/p3_B_S1.raw.vcf
freebayes-parallel p15_7_S3/reference/ref.txt 4 -p 1 -P 0 -C 1 -F 0.001 -q 13 -m
60 --strict-vcf -f p15_7_S3/reference/ref.fa ../p15_7_S3/snps.bam >
filter2/p15_7_S3.raw.vcf
freebayes-parallel p2_2_S3/reference/ref.txt 4 -p 1 -P 0 -C 1 -F 0.001 -q 13 -m
60 --strict-vcf -f p2_2_S3/reference/ref.fa ../p2_2_S3/snps.bam >
filter1/p2_2_S3.raw.vcf
freebayes-parallel p15_F_S1/reference/ref.txt 4 -p 1 -P 0 -C 1 -F 0.001 -q 13 -m
60 --strict-vcf -f p15_F_S1/reference/ref.fa ../p15_F_S1/snps.bam >
filter1/p15_F_S1.raw.vcf
```

```
freebayes-parallel p2_6_S4/reference/ref.txt 4 -p 1 -P 0 -C 1 -F 0.001 -q 13 -m
60 --strict-vcf -f p2_6_S4/reference/ref.fa ../p2_6_S4/snps.bam >
filter2/p2_6_S4.raw.vcf
freebayes-parallel p2_2_S2/reference/ref.txt 4 -p 1 -P 0 -C 1 -F 0.001 -q 13 -m
60 --strict-vcf -f p2_2_S2/reference/ref.fa ../p2_2_S2/snps.bam >
filter1/p2_2_S2.raw.vcf
freebayes-parallel p15_F_S5/reference/ref.txt 4 -p 1 -P 0 -C 1 -F 0.001 -q 13 -m
60 --strict-vcf -f p15_F_S5/reference/ref.fa ../p15_F_S5/snps.bam >
filter1/p15_F_S5.raw.vcf
freebayes-parallel p3_B_S2/reference/ref.txt 4 -p 1 -P 0 -C 1 -F 0.001 -q 13 -m
60 --strict-vcf -f p3_B_S2/reference/ref.fa ../p3_B_S2/snps.bam >
filter2/p3_B_S2.raw.vcf
freebayes-parallel p3IPTG_A_S1b/reference/ref.txt 4 -p 1 -P 0 -C 1 -F 0.001 -q
13 -m 60 --strict-vcf -f p3IPTG_A_S1b/reference/ref.fa ../p3IPTG_A_S1b/snps.bam
> filter1/p3IPTG_A_S1b.raw.vcf
```

To get the total number of mapped reads:

```
for fil in glob.glob('./p*/*.bam'):
    ide = fil.split('/')[1]
    # get the total number of reads of a BAM file (may include unmapped and duplicated multi-a
    print('samtools view -c {} >> total_readcount.txt'.format(fil))
    print('samtools view -c {} -F 260 >> total_readcount_mapped.txt'.format(fil))
```

Finally we annotate the effect of the variants using SnpEff. This requires to add the custom annotation to the sources, specifying that the translation code is set to 4 for *M. pneumoniae*.

1. Download SnpEff and edited the config (in software_crg/SnpEff) to include the C5 and C20 refseq references
2. Build the custom db:

   ```
   java -jar snpEff.jar build -genbank -v C5ali
   java -jar snpEff.jar build -genbank -v C20ali
   ```

3. Annotate vcf files in bash (in directory of the software)

   ```
   for fil in `ls ./filter1/p*.vcf`; do java -Xmx8g -jar snpEff.jar C5ali $fil > $fil.ann; dc
   for fil in `ls ./filter2/p*.vcf`; do java -Xmx8g -jar snpEff.jar C20ali $fil > $fil.ann; c
   ```

1.2 2. Data loading

We will parse the variations to keep the columns of interest to perform the exploratory analysis. Dataframes include the following information (by column header): - SAMPLE: sample identifier includes information about the passage (e.g. p3IPTG is the passage 3 induced) - PASS: passage (2,3 or 15) - POS: genome loci (base pair position) where the variant is found - QUAL: estimate of the probability that there is a polymorphism at the loci described by the record - TOT: total number of reads covering a loci - REFN: total number of reads covering a loci matching the reference - ALTN: number of reads presenting a variant - REF: reference sequence - ALT: alternative sequence - EFF: potential effect of the variant as given by snpeff - IMPACT: potential impact of the variant, can be LOW (synonimous mutations), MODERATE (missense), HIGH (non-synonimous mutations,

start lost, stop lost), or MODIFIER (when it occurs in an intergenic region) - AFF: gene affected - MUT: mutation (in nucleotide if not coding in amino acid if coding) - ANN_TYPE: annotation of the loci, can be intergenic, gene, or cassette (covering the principal elements in the cassette)

[7]:
```python
# This will concat all the annotated vcf files keeping the fields of interest
data1 = scripts.parse_variations('./filter1/*.raw.vcf.ann', order1, genome=5, fname='./results/data1.pickle')
data2 = scripts.parse_variations('./filter2/*.raw.vcf.ann', order2, genome=20, fname='./results/data2.pickle')
```

```
Loading data for genome 5
Loading data for genome 20
```

We can now explore the variants, for example to extract those present in the cassette with high impact (non-synonimous) in samples induced (assigned as passage 18 despite they are passage 3), could be retrieved as:

[8]:
```python
# Example:
## Notice the information is grouped by position, we will deal with this in section 3.
data1[(data1['ANN_TYPE']=='cassette') & (data1['IMPACT']=='HIGH') & (data1['PASS']==18)].sort_values('FRAC')
```

[8]:

	SAMPLE	PASS	POS	QUAL	TOT	REFN	ALTN	FRAC	\
443045	p3IPTG_A_S1	18	566372	2.184800e-15	677	676	1	0.147929	
443082	p3IPTG_A_S1	18	566622	0.000000e+00	647	646	1	0.154799	
443002	p3IPTG_A_S1	18	565989	2.863080e-15	582	581	1	0.172117	
443294	p3IPTG_A_S1	18	568518	7.700020e-15	577	576	1	0.173611	
443402	p3IPTG_A_S1	18	569451	6.132760e-15	572	571	1	0.175131	
…	…	…	…	…	…	…	…	…	
443663	p3IPTG_A_S1	18	571681	0.000000e+00	481	469	12	2.558635	
495218	p3IPTG_A_S1b	18	571681	7.895900e-15	191	182	9	4.945055	
495059	p3IPTG_A_S1b	18	569127	8.076710e-15	235	210	25	11.904762	
443366	p3IPTG_A_S1	18	569125	0.000000e+00	534	463	71	15.334773	
443009	p3IPTG_A_S1	18	566053	1.947940e-13	559	446	113	25.336323	

	REF	ALT	EFF	\
443045	CTA	TTT	stop_gained	
443082	A	C	stop_gained	
443002	TCCCTCG	TCCTCG	frameshift_variant	
443294	G	A	stop_gained	
443402	C	A	stop_gained	
…	…	…	…	
443663	ATTTTTTTGATA	ATTTTTTTGATA	frameshift_variant	
495218	ATTTTTTTTGATACT	ATTTCTTTAGAAACA	stop_gained	
495059	CAAACT	CAACT	frameshift_variant	
443366	ATCAAACT	GTCAACC	frameshift_variant&missense_variant	
443009	CGCAAA	TGCTAT	stop_gained	

```
       IMPACT    AFF                MUT  ANN_TYPE
443045   HIGH  LacI4       p.LeuGly102*  cassette
443082   HIGH  LacI4           p.Tyr19*  cassette
443002   HIGH  LacI4         p.Gly230fs  cassette
443294   HIGH  cas9B        p.Gln1101*  cassette
443402   HIGH  cas9B          p.Glu790*  cassette
…         …     …                 …        …
443663   HIGH  cas9B          p.Asn46fs  cassette
495218   HIGH  cas9B p.SerIleLysLys42*  cassette
495059   HIGH  cas9B         p.Phe897fs  cassette
443366   HIGH  cas9B         p.Lys896fs  cassette
443009   HIGH  LacI4       p.LeuArg208*  cassette

[101 rows x 15 columns]
```

[9]:
```python
# For C20 we have snpcalls2 in the same format
data2[(data2['ANN_TYPE']=='cassette') & (data2['IMPACT']=='HIGH') &␣
 ↪(data2['PASS']==3)].sort_values('FRAC')
```

[9]:
```
          SAMPLE  PASS     POS          QUAL  TOT  REFN  ALTN      FRAC  \
225393  p3_B_S1     3  786189  0.000000e+00  280   279     1  0.358423
225340  p3_B_S1     3  785573  2.991160e-15  253   252     1  0.396825
225346  p3_B_S1     3  785643  0.000000e+00  252   251     1  0.398406
264083  p3_B_S2     3  566670  0.000000e+00  226   225     1  0.444444
225402  p3_B_S1     3  787394  0.000000e+00  150   149     1  0.671141
277049  p3_B_S2     3  785709  1.702880e-15  258   256     2  0.781250
277050  p3_B_S2     3  785714  1.642610e-15  255   253     2  0.790514
209632  p3_B_S1     3  566676  1.994980e-14  226   224     2  0.892857
209630  p3_B_S1     3  566634  2.558530e-14  112   111     1  0.900901
225397  p3_B_S1     3  786318  0.000000e+00   98    97     1  1.030928
225356  p3_B_S1     3  785712  0.000000e+00  264   261     3  1.149425

                REF          ALT                EFF IMPACT         AFF  \
225393         TTAC         CTAT        stop_gained   HIGH    cas2 cat
225340  GAAAAAAATCAC GAAAAAAAATCAC  frameshift_variant   HIGH    cas2 cat
225346            C            T        stop_gained   HIGH    cas2 cat
264083       CTTTGC        CTTGC  frameshift_variant   HIGH  cas1 LacI4
225402         AGCG         TGCA          stop_lost   HIGH  cas2 lacI4
277049          GTA           GA  frameshift_variant   HIGH    cas2 cat
277050   GAAAAATAAGC   GAAAATAAGC  frameshift_variant   HIGH    cas2 cat
209632         CATA         TATT         start_lost   HIGH  cas1 LacI4
209630        AGGCT       AGGGCT  frameshift_variant   HIGH  cas1 LacI4
225397        GCCTG       GCCCTG  frameshift_variant   HIGH  cas2 lacI4
225356      AAGAAAA      TAGTAAT        stop_gained   HIGH    cas2 cat

                          MUT  ANN_TYPE
```

```
225393                p.Gln209*  cassette
225340                 p.Ile5fs  cassette
225346                 p.Gln26*  cassette
264083                 p.Lys3fs  cassette
225402  p.TerArg373LeuGlnext*?  cassette
277049                p.Val48fs  cassette
277050                p.Asn51fs  cassette
209632                  p.Met1?  cassette
209630                p.Val16fs  cassette
225397                p.Val16fs  cassette
225356           p.LysLysAsn49*  cassette
```

[644]:

```
# Save supplementary 1
sup1 = './results/suptableS1.xlsx'
if not os.path.isfile(sup1):
    writer = pd.ExcelWriter(sup1, engine='xlsxwriter')
    for c, data in zip(['C5_', 'C20_'], [data1, data2]):
        for sample in set(data['SAMPLE']):
            subdata = data[data['SAMPLE']==sample].copy()
            subdata.to_excel(writer, sheet_name=c+sample)
    writer.save()
```

1.3 3. Rate and fraction of mutations in cassette compared to other distributions (supplementary figure 1)

Explore the frequency at which we found a variant within the cassette compared to other types of annotations and also their representation within the population by means of the fraction values.

[665]:

```
def plot_percentage(df, genome=5):
    """ Plot to show the percentage of variants mapping to each type of␣
↪annotation """
    rs = {}
    c = 0
    lens = {}
    lens['gene'] = len(ingene)
    lens['intergenic'] = len(outgene)
    lens['essential'] = len(posE)
    lens['non-essential'] = len(posN)
    if genome==5:
        lens['chromosome'] = len(genome_C5)
        lens['cassette'] = len(cas1)
    else:
        lens['chromosome'] = len(genome_C20)
        lens['cassette'] = len(cas12)
    # Extract positions in passage 2
    for pas in set(df['PASS']):
```

```
        for sample in set(df[df['PASS']==pas]['SAMPLE']):
            for impact in set(df['IMPACT']):
                rs[c] = [pas,
                         100*len(set(df[(df['PASS']==pas) &␣
↪(df['IMPACT']==impact)  & (df['SAMPLE']==sample)]['POS']))/
↪lens['chromosome'],
                         sample, 'TOTAL', impact]
                c+=1
            rs[c] = [pas, 100*len(set(df[(df['PASS']==pas)  &␣
↪(df['SAMPLE']==sample)]['POS']))/lens['chromosome'],sample, 'chromosome',␣
↪'TOTAL']
            c+=1
            for ann in set(df['ANN_TYPE']):
                if ann in lens:
                    rs[c] = [pas,
                             100*len(set(df[(df['PASS']==pas) &␣
↪(df['ANN_TYPE']==ann)  & (df['SAMPLE']==sample)]['POS']))/lens[ann],
                             sample, ann, 'TOTAL']
                    c+=1
                    for impact in set(df['IMPACT']):
                        rs[c] = [pas,
                                 100*len(set(df[(df['PASS']==pas) &␣
↪(df['ANN_TYPE']==ann) & (df['IMPACT']==impact)  &␣
↪(df['SAMPLE']==sample)]['POS']))/lens[ann],
                                 sample, ann, impact]
                        c+=1
    rs = pd.DataFrame.from_dict(rs, orient='index')
    rs.columns = ['PASS', 'PERC', 'sample', 'annotation', 'IMPACT']
    rs['HUE'] = rs['annotation']+rs['IMPACT']
    return rs.sort_values('annotation')

subdf1 = data1[(data1['FRAC']<100) & (data1['ALTN']>=2)].
↪sort_values(['ANN_TYPE', 'PASS']).copy()
subdf2 = data2[(data2['FRAC']<100) & (data2['ALTN']>=2)].
↪sort_values(['ANN_TYPE', 'PASS']).copy()

plot1 = plot_percentage(subdf1)
plot2 = plot_percentage(subdf2)

subdf1['log2Frac'] = np.log2(subdf1['FRAC'])
subdf1['Condition'] = ['{}'.format(i) if i!=18 else '3IPTG' for i in␣
↪subdf1['PASS']]
subdf1['annotation'] = subdf1['ANN_TYPE']

subdf2['log2Frac'] = np.log2(subdf2['FRAC'])
```

```
subdf2['Condition'] = ['{}'.format(i) if i!=18 else '3IPTG' for i in␣
↪subdf2['PASS']]
subdf2['annotation'] = subdf2['ANN_TYPE']

plt.close('all')
plt.figure(figsize=(10, 10))
plt.subplot(2,3,1)
sns.countplot(x='Condition', hue='annotation', data=subdf1[(subdf1['ANN_TYPE'].
↪isin(['cassette', 'gene', 'intergenic']))])

plt.subplot(2,3,2)
sns.barplot(x='PASS', y='PERC', hue='annotation',␣
↪data=plot1[(plot1['IMPACT']=='TOTAL') & (plot1['annotation'].
↪isin(['cassette', 'gene', 'intergenic']))])
plt.xticks([0,1,2,3], ['2', '3', '15', '3IPTG'])
plt.xlabel('Condition')
plt.ylabel('Percentage')
plt.title('C5 samples')

plt.subplot(2,3,3)
sns.boxplot(y='log2Frac', x='Condition', hue='annotation',␣
↪data=subdf1[(subdf1['log2Frac']>0) & (subdf1['ANN_TYPE'].isin(['cassette',␣
↪'gene', 'intergenic']))])
plt.xlabel('Condition')
plt.ylabel('log2(Fraction)')

#plt.ylim(0,3.2)
plt.subplot(2,3,4)
sns.countplot(x='PASS', hue='annotation', data=subdf2[(subdf2['ANN_TYPE'].
↪isin(['cassette', 'gene', 'intergenic']))])
plt.xlabel('Condition')

plt.subplot(2,3,5)
sns.barplot(x='PASS', y='PERC', hue='annotation',␣
↪data=plot2[(plot2['IMPACT']=='TOTAL') & (plot2['annotation'].
↪isin(['cassette', 'gene', 'intergenic']))])
plt.xlabel('Condition')
plt.ylabel('Percentage')
plt.title('C20 samples')
#plt.ylim(0,3.2)

plt.subplot(2,3,6)
sns.boxplot(y='log2Frac', x='PASS', hue='annotation',␣
↪data=subdf2[(subdf2['log2Frac']>0) & (subdf2['ANN_TYPE'].isin(['cassette',␣
↪'gene', 'intergenic']))])
plt.xlabel('Condition')
```

```
plt.ylabel('log2(Fraction)')

plt.tight_layout()
plt.savefig('./results/supfigS1.svg')
```

```
Canvas(toolbar=Toolbar(toolitems=[('Home', 'Reset original view', 'home', 'home'), ('Back', 'Ba
```

Let's calculate the statistics for this comparisons:

[666]:
```
percentage_pvalues = {}
fraction_pvalues = {}

perc1 = plot1[(plot1['IMPACT']=='TOTAL') & (plot1['annotation'].
 ↪isin(['cassette', 'gene', 'intergenic']))].copy()
perc2 = plot2[(plot2['IMPACT']=='TOTAL') & (plot2['annotation'].
 ↪isin(['cassette', 'gene', 'intergenic']))].copy()

frac1 = subdf1[(subdf1['log2Frac']>0) & (subdf1['ANN_TYPE'].isin(['cassette',␣
 ↪'gene', 'intergenic']))].copy()
frac2 = subdf2[(subdf2['log2Frac']>0) & (subdf2['ANN_TYPE'].isin(['cassette',␣
 ↪'gene', 'intergenic']))].copy()

c = 0
for pas in set(perc1['PASS']):
    x = perc1[(perc1['PASS']==pas) & (perc1['annotation']=='cassette')]['PERC']
    for annot in ['gene', 'intergenic']:
        y = perc1[(perc1['PASS']==pas) & (perc1['annotation']==annot)]['PERC']
        percentage_pvalues[c] = ['C5', pas, annot, round(mannwhitneyu(x, y)[1]/
 ↪2, 4)]
        c+=1
for pas in set(perc2['PASS']):
    x = perc2[(perc2['PASS']==pas) & (perc2['annotation']=='cassette')]['PERC']
    for annot in ['gene', 'intergenic']:
        y = perc2[(perc2['PASS']==pas) & (perc2['annotation']==annot)]['PERC']
        percentage_pvalues[c] = ['C20', pas, annot,round(mannwhitneyu(x, y)[1]/
 ↪2, 4)]
        c+=1

c = 0
for pas in set(frac1['PASS']):
    x = frac1[(frac1['PASS']==pas) &␣
 ↪(frac1['annotation']=='cassette')]['log2Frac']
    for annot in ['gene', 'intergenic']:
        y = frac1[(frac1['PASS']==pas) &␣
 ↪(frac1['annotation']==annot)]['log2Frac']
```

```
        fraction_pvalues[c] = ['C5', pas, annot, round(mannwhitneyu(x, y)[1]/2,␣
↪4)]
        c+=1
for pas in set(frac2['PASS']):
    x = frac2[(frac2['PASS']==pas) &␣
↪(frac2['annotation']=='cassette')]['log2Frac']
    for annot in ['gene', 'intergenic']:
        y = frac2[(frac2['PASS']==pas) &␣
↪(frac2['annotation']==annot)]['log2Frac']
        fraction_pvalues[c] = ['C20', pas, annot,round(mannwhitneyu(x, y)[1]/2,␣
↪4)]
        c+=1
```

[667]:

```
print('Average rate of mutations per base in the cassette', np.
↪mean(perc1[(perc1['annotation']=='cassette')]['PERC']))
print('Average rate of mutations per base in the intergenic', np.
↪mean(perc1[(perc1['annotation']=='intergenic')]['PERC']))
print('Average rate of mutations per base in the gene', np.
↪mean(perc1[(perc1['annotation']=='gene')]['PERC']))

print('Average rate of mutations per base in the cassette', np.
↪mean(perc2[(perc2['annotation']=='cassette')]['PERC']))
print('Average rate of mutations per base in the intergenic', np.
↪mean(perc2[(perc2['annotation']=='intergenic')]['PERC']))
print('Average rate of mutations per base in the gene', np.
↪mean(perc2[(perc2['annotation']=='gene')]['PERC']))
```

```
Average rate of mutations per base in the cassette 2.825228856974889
Average rate of mutations per base in the intergenic 1.8428205685101458
Average rate of mutations per base in the gene 1.4096396774343913
Average rate of mutations per base in the cassette 0.9750566893424035
Average rate of mutations per base in the intergenic 2.42446081044616
Average rate of mutations per base in the gene 1.8454062368899453
```

[668]:

```
percentage_pvalues
```

[668]:

```
{0: ['C5', 18, 'gene', 0.0613],
 1: ['C5', 18, 'intergenic', 0.1746],
 2: ['C5', 2, 'gene', 0.0476],
 3: ['C5', 2, 'intergenic', 0.0476],
 4: ['C5', 3, 'gene', 0.1746],
 5: ['C5', 3, 'intergenic', 0.1746],
 6: ['C5', 15, 'gene', 0.0613],
 7: ['C5', 15, 'intergenic', 0.0613],
 8: ['C20', 2, 'gene', 0.0613],
 9: ['C20', 2, 'intergenic', 0.0613],
```

```
 10: ['C20', 3, 'gene', 0.0613],
 11: ['C20', 3, 'intergenic', 0.0613],
 12: ['C20', 15, 'gene', 0.0613],
 13: ['C20', 15, 'intergenic', 0.0613]}
```

[669]:
```
print('Average rate of mutations per base in the cassette', np.
 ↪percentile(frac1[(frac1['annotation']=='cassette')  &␣
 ↪(frac1['log2Frac']>0)]['FRAC'], 50))
print('Average rate of mutations per base in the intergenic', np.
 ↪percentile(frac1[(frac1['annotation']=='intergenic') & (frac1['log2Frac']>0)␣
 ↪]['FRAC'], 50))
print('Average rate of mutations per base in the gene', np.
 ↪percentile(frac1[(frac1['annotation']=='gene')&␣
 ↪(frac1['log2Frac']>0)]['FRAC'], 50))

print('Average rate of mutations per base in the cassette', np.
 ↪median(frac2[(frac2['annotation']=='cassette')]['FRAC']))
print('Average rate of mutations per base in the intergenic', np.
 ↪median(frac2[(frac2['annotation']=='intergenic')]['FRAC']))
print('Average rate of mutations per base in the gene', np.
 ↪median(frac2[(frac2['annotation']=='gene')]['log2Frac']))
```

```
Average rate of mutations per base in the cassette 1.492537260055542
Average rate of mutations per base in the intergenic 1.5564202070236206
Average rate of mutations per base in the gene 1.470588207244873
Average rate of mutations per base in the cassette 1.5151515
Average rate of mutations per base in the intergenic 1.3513514
Average rate of mutations per base in the gene 0.38646838
```

[670]:
```
fraction_pvalues
```

[670]:
```
{0: ['C5', 18, 'gene', 0.0117],
 1: ['C5', 18, 'intergenic', 0.2241],
 2: ['C5', 2, 'gene', 0.0],
 3: ['C5', 2, 'intergenic', 0.0],
 4: ['C5', 3, 'gene', 0.1343],
 5: ['C5', 3, 'intergenic', 0.0988],
 6: ['C5', 15, 'gene', 0.1662],
 7: ['C5', 15, 'intergenic', 0.0021],
 8: ['C20', 2, 'gene', 0.0002],
 9: ['C20', 2, 'intergenic', 0.0016],
 10: ['C20', 3, 'gene', 0.0],
 11: ['C20', 3, 'intergenic', 0.0],
 12: ['C20', 15, 'gene', 0.0],
 13: ['C20', 15, 'intergenic', 0.002]}
```

1.4 4. Location of the mutations within the cassettes (supplementary figure 2)

[682]:
```
subdf1 = data1[(data1['FRAC']<100) & (data1['ALTN']>=2) & (data1['QUAL']>0.0)].
 ↪sort_values(['ANN_TYPE', 'PASS']).copy()
subdf2 = data2[(data2['FRAC']<100) & (data2['ALTN']>=2) & (data2['QUAL']>0.0)].
 ↪sort_values(['ANN_TYPE', 'PASS']).copy()

plot1 = plot_percentage(subdf1)
plot2 = plot_percentage(subdf2)

subdf1['log2Frac'] = np.log2(subdf1['FRAC'])
subdf1['Condition'] = ['{}'.format(i) if i!=18 else '3IPTG' for i in␣
 ↪subdf1['PASS']]
subdf1['annotation'] = subdf1['ANN_TYPE']

subdf2['log2Frac'] = np.log2(subdf2['FRAC'])
subdf2['Condition'] = ['{}'.format(i) if i!=18 else '3IPTG' for i in␣
 ↪subdf2['PASS']]
subdf2['annotation'] = subdf2['ANN_TYPE']

plt.close('all')
plt.figure(figsize=(15, 10))
plt.subplot(2,2,1)
sns.countplot(x='AFF', hue='Condition', data=subdf1[(subdf1['ANN_TYPE'].
 ↪isin(['cassette']))])
plt.xticks(rotation=45, ha='right')
plt.xlabel('Element')

plt.subplot(2,2,2)
sns.swarmplot(y='FRAC', x='PASS', hue='AFF', data=subdf1[(subdf1['log2Frac']>0)␣
 ↪& (subdf1['ANN_TYPE'].isin(['cassette']))])
plt.xlabel('Passage')
plt.ylabel('Fraction')
plt.title('C5 samples')
plt.legend(bbox_to_anchor=(1.05, 1))


#plt.ylim(0,3.2)
plt.subplot(2,2,3)
sns.countplot(x='AFF', hue='Condition', data=subdf2[(subdf2['ANN_TYPE'].
 ↪isin(['cassette']))])
plt.xticks(rotation=45, ha='right')
plt.xlabel('Element')


plt.subplot(2,2,4)
```

```
sns.swarmplot(y='FRAC', x='PASS', hue='AFF', data=subdf2[(subdf2['log2Frac']>0)␣
↪& (subdf2['ANN_TYPE'].isin(['cassette']))])
plt.xlabel('Passage')
plt.ylabel('Fraction')
plt.title('C20 samples')
plt.legend(bbox_to_anchor=(1.05, 1))

plt.tight_layout()
plt.savefig('./results/supfigS2.svg')
```

```
Canvas(toolbar=Toolbar(toolitems=[('Home', 'Reset original view', 'home', 'home'), ('Back', 'Ba
```

1.5 5. Exploration of the effect of the mutations explaining the escape rate (supplementary figure 3)

Integrating the previous results we will explore the mutations that could mainly explain the rate of cell escaping the killswitch circuit (we use the median value to filter out non-significant positions)

[679]:
```
plt.figure(figsize=(10,10))
plt.subplot(2,1,1)
sns.swarmplot(x='AFF', y='FRAC', hue='IMPACT',data=subdf1[(subdf1['FRAC']>=1.5)␣
↪& (subdf1['ANN_TYPE']=='cassette')])
plt.xticks(rotation=45, ha='right')
plt.xlabel('Element')
plt.ylabel('Fraction')

plt.subplot(2,1,2)
sns.swarmplot(x='AFF', y='FRAC', hue='IMPACT',data=subdf2[(subdf2['FRAC']>=1.5)␣
↪& (subdf2['ANN_TYPE']=='cassette')])
plt.xticks(rotation=45, ha='right')
plt.xlabel('Element')
plt.ylabel('Fraction')

plt.tight_layout()
plt.savefig('./results/supfigS3.svg')
```

```
Canvas(toolbar=Toolbar(toolitems=[('Home', 'Reset original view', 'home', 'home'), ('Back', 'Ba
```

[677]:
```
selected = data1[(data1['PASS']==18) & (data1['ANN_TYPE'].isin(['cassette']))].
↪sort_values(['FRAC'])
selected['TOP'] = [1 if z>=1.5 else 0 for z in selected['FRAC']]
selected.to_excel('./results/suptableS2.xlsx')
selected
```

[677]:

```
                SAMPLE  PASS     POS          QUAL  TOT  REFN  ALTN       FRAC  \
443045    p3IPTG_A_S1    18  566372  2.184800e-15  677   676     1   0.147929
443032    p3IPTG_A_S1    18  566245  1.137610e-15  670   669     1   0.149477
443043    p3IPTG_A_S1    18  566351  0.000000e+00  655   654     1   0.152905
443077    p3IPTG_A_S1    18  566562  0.000000e+00  655   654     1   0.152905
443082    p3IPTG_A_S1    18  566622  0.000000e+00  647   646     1   0.154799
…                  …     …       …            …    …     …     …          …
494861   p3IPTG_A_S1b    18  566416  3.471600e-14  281   255    26  10.196078
495059   p3IPTG_A_S1b    18  569127  8.076710e-15  235   210    25  11.904762
443366    p3IPTG_A_S1    18  569125  0.000000e+00  534   463    71  15.334773
494831   p3IPTG_A_S1b    18  566053  0.000000e+00  271   224    47  20.982143
443009    p3IPTG_A_S1    18  566053  1.947940e-13  559   446   113  25.336323

             REF      ALT                               EFF    IMPACT  \
443045       CTA      TTT                       stop_gained      HIGH
443032         G        T                  missense_variant  MODERATE
443043         T        C                  missense_variant  MODERATE
443077         T        G                synonymous_variant       LOW
443082         A        C                       stop_gained      HIGH
…              …        …                                 …         …
494861   GGTGCGT  CGTTCTT                  missense_variant  MODERATE
495059    CAAACT    CAACT                frameshift_variant      HIGH
443366   ATCAAACT GTCAACC  frameshift_variant&missense_variant      HIGH
494831     CGCAA    CACAT                  missense_variant  MODERATE
443009    CGCAAA   TGCTAT                       stop_gained      HIGH

           AFF                    MUT  ANN_TYPE  TOP
443045   LacI4          p.LeuGly102*  cassette    0
443032   LacI4           p.Ala145Asp  cassette    0
443043   LacI4           p.Met110Val  cassette    0
443077   LacI4            p.Ala39Ala  cassette    0
443082   LacI4              p.Tyr19*  cassette    0
…            …                     …         …    …
494861   LacI4  p.HisAlaPro86GlnGluArg  cassette    1
495059   cas9B            p.Phe897fs  cassette    1
443366   cas9B            p.Lys896fs  cassette    1
494831   LacI4       p.LeuArg208MetCys  cassette    1
443009   LacI4          p.LeuArg208*  cassette    1

[1237 rows x 16 columns]
```

[678]:

```python
selected[selected['POS'].isin([566423, 571681, 566053])]
```

[678]:

```
                SAMPLE  PASS     POS          QUAL  TOT  REFN  ALTN       FRAC  \
443045    p3IPTG_A_S1    18  566372  2.184800e-15  677   676     1   0.147929
443032    p3IPTG_A_S1    18  566245  1.137610e-15  670   669     1   0.149477
443043    p3IPTG_A_S1    18  566351  0.000000e+00  655   654     1   0.152905
```

```
443077    p3IPTG_A_S1    18  566562  0.000000e+00  655   654      1   0.152905
443082    p3IPTG_A_S1    18  566622  0.000000e+00  647   646      1   0.154799
…                 …   …     …             …   …     …    …     …
494861   p3IPTG_A_S1b    18  566416  3.471600e-14  281   255     26  10.196078
495059   p3IPTG_A_S1b    18  569127  8.076710e-15  235   210     25  11.904762
443366    p3IPTG_A_S1    18  569125  0.000000e+00  534   463     71  15.334773
494831   p3IPTG_A_S1b    18  566053  0.000000e+00  271   224     47  20.982143
443009    p3IPTG_A_S1    18  566053  1.947940e-13  559   446    113  25.336323

              REF      ALT                                EFF    IMPACT  \
443045        CTA      TTT                        stop_gained      HIGH
443032          G        T                   missense_variant  MODERATE
443043          T        C                   missense_variant  MODERATE
443077          T        G                 synonymous_variant       LOW
443082          A        C                        stop_gained      HIGH
…             …      …                                    …       …
494861    GGTGCGT  CGTTCTT                   missense_variant  MODERATE
495059      CAAACT    CAACT                 frameshift_variant      HIGH
443366    ATCAAACT  GTCAACC  frameshift_variant&missense_variant      HIGH
494831       CGCAA    CACAT                   missense_variant  MODERATE
443009      CGCAAA   TGCTAT                        stop_gained      HIGH

           AFF                    MUT  ANN_TYPE  TOP
443045  LacI4            p.LeuGly102*  cassette    0
443032  LacI4             p.Ala145Asp  cassette    0
443043  LacI4             p.Met110Val  cassette    0
443077  LacI4              p.Ala39Ala  cassette    0
443082  LacI4                p.Tyr19*  cassette    0
…          …                      …       …    …
494861  LacI4  p.HisAlaPro86GlnGluArg  cassette    1
495059  cas9B              p.Phe897fs  cassette    1
443366  cas9B              p.Lys896fs  cassette    1
494831  LacI4       p.LeuArg208MetCys  cassette    1
443009  LacI4            p.LeuArg208*  cassette    1

[1237 rows x 16 columns]
```

[681]:
```
data2[(data2['ANN_TYPE']=='cassette') & (data2['FRAC']>10)]
```

[681]:
```
            SAMPLE  PASS     POS          QUAL  TOT  REFN  ALTN          FRAC  \
54232      p2_6_S2     2  566587  3.889600e-06    4     3     1     33.333332
163230     p2_6_S4     2  780968  4.857580e+03  156     1   155  15500.000000
163253     p2_6_S4     2  781179  6.057650e-15   80    71     9     12.676056
163342     p2_6_S4     2  786151  5.734500e+03  182     1   181  18100.000000
163362     p2_6_S4     2  786347  0.000000e+00   14    11     3     27.272728
225311     p3_B_S1     3  780968  6.689500e+03  214     2   212  10600.000000
277012     p3_B_S2     3  780964  5.288950e+03  170     2   168   8400.000000
```

```
277062   p3_B_S2    3  786151  7.229180e+03  228     1   227  22700.000000
413818  p15_7_S5   15  780968  3.700950e+03  118     1   117  11700.000000
413844  p15_7_S5   15  785418  4.709100e-15    7     6     1     16.666666
413909  p15_7_S5   15  786347  6.060810e-12   10     6     4     66.666664

                         REF                       ALT                  EFF  \
54232                      G                         A     missense_variant
163230                 AGTCC                     CGTCC  upstream_gene_variant
163253  GACCCAACTGCCACGAAGTTTT  GACCCAACTGCCTTGATGTTAT  upstream_gene_variant
163342                   TCT                       TTT   synonymous_variant
163362                     A                         G     missense_variant
225311                 AGTCC                     CGTCC  upstream_gene_variant
277012             GGCTAGTCC                 GGCTCGTCC  upstream_gene_variant
277062                TCTGTG                    TTTGTG   synonymous_variant
413818                 AGTCC                     CGTCC  upstream_gene_variant
413844                     A                         C   synonymous_variant
413909                     A                         G     missense_variant

          IMPACT                   AFF                                   MUT  \
54232   MODERATE            cas1 LacI4                             p.Thr31Ile
163230  MODIFIER  cas2 regulator gRNA5                            c.-4520T>G
163253  MODIFIER  cas2 regulator gRNA2  c.-4751_-4743delAAACTTCGTinsTAACATCAA
163342       LOW             cas2 cat                            p.Val195Val
163362  MODERATE            cas2 lacI4                             p.Tyr24Cys
225311  MODIFIER  cas2 regulator gRNA5                            c.-4520T>G
277012  MODIFIER  cas2 regulator gRNA5                            c.-4520T>G
277062       LOW             cas2 cat                            p.Val195Val
413818  MODIFIER  cas2 regulator gRNA5                            c.-4520T>G
413844       LOW            cas2 cas9B                           p.Thr1339Thr
413909  MODERATE            cas2 lacI4                             p.Tyr24Cys

        ANN_TYPE
54232   cassette
163230  cassette
163253  cassette
163342  cassette
163362  cassette
225311  cassette
277012  cassette
277062  cassette
413818  cassette
413844  cassette
413909  cassette
```

[661]:

```
selected[selected['IMPACT']=='HIGH']
```

[661]:

	SAMPLE	PASS	POS	QUAL	TOT	REFN	ALTN	FRAC	\
494840	p3IPTG_A_S1b	18	566136	0.000000e+00	243	238	5	2.100840	
494818	p3IPTG_A_S1b	18	565929	6.208090e-15	232	227	5	2.202643	
443663	p3IPTG_A_S1	18	571681	0.000000e+00	481	469	12	2.558635	
495218	p3IPTG_A_S1b	18	571681	7.895900e-15	191	182	9	4.945055	
495059	p3IPTG_A_S1b	18	569127	8.076710e-15	235	210	25	11.904762	
443366	p3IPTG_A_S1	18	569125	0.000000e+00	534	463	71	15.334773	
443009	p3IPTG_A_S1	18	566053	1.947940e-13	559	446	113	25.336323	

	REF	ALT	\
494840	TAAGCGGGTCCCATCTTCGT	TTAGCCGGTCCCATCTTCGT	
494818	AACAATCCCCTCATTTAA	AACAATCCCCTTTTTTTA	
443663	ATTTTTTTTGATA	ATTTTTTTGATA	
495218	ATTTTTTTTGATACT	ATTTCTTTAGAAACA	
495059	CAAACT	CAACT	
443366	ATCAAACT	GTCAACC	
443009	CGCAAA	TGCTAT	

	EFF	IMPACT	AFF	MUT	\
494840	stop_gained	HIGH	LacI4	p.ArgLeu180*	
494818	stop_gained	HIGH	LacI4	p.LeuAsnGlu245*	
443663	frameshift_variant	HIGH	cas9B	p.Asn46fs	
495218	stop_gained	HIGH	cas9B	p.SerIleLysLys42*	
495059	frameshift_variant	HIGH	cas9B	p.Phe897fs	
443366	frameshift_variant&missense_variant	HIGH	cas9B	p.Lys896fs	
443009	stop_gained	HIGH	LacI4	p.LeuArg208*	

	ANN_TYPE
494840	cassette
494818	cassette
443663	cassette
495218	cassette
495059	cassette
443366	cassette
443009	cassette

1.6 NOT USED

[635]:
```
subdf1[(subdf1['FRAC']>=25) & (subdf1['ANN_TYPE']=='intergenic')]
```

[635]:

	SAMPLE	PASS	POS	QUAL	TOT	REFN	ALTN	FRAC	\
20557	p2_2_S1	2	629173	1.383230e-14	30	17	13	76.470589	
33962	p2_2_S2	2	113969	2.790510e-07	6	4	2	50.000000	
35494	p2_2_S2	2	141270	4.924100e-14	59	40	19	47.500000	
57248	p2_2_S2	2	528761	2.688110e-14	45	33	12	36.363636	
63072	p2_2_S2	2	629170	2.953720e-15	57	38	19	50.000000	

85859	p2_2_S3	2	141267	1.219870e-13	159	97	62	63.917526
161975	p3_D_S2	3	195424	4.551630e-13	413	239	174	72.803345
214268	p3_D_S2	3	629169	2.384300e-13	355	212	143	67.452827
274642	p3_D_S3	3	528755	8.763400e-14	140	84	56	66.666664
280252	p3_D_S3	3	618608	8.492520e-09	7	5	2	40.000000
406624	p3IPTG_A_S1	18	141270	2.114290e-13	235	158	77	48.734177
447613	p3IPTG_A_S1	18	618608	2.102690e-14	9	7	2	28.571428
448420	p3IPTG_A_S1	18	629167	7.256610e-14	246	139	107	76.978416
493008	p3IPTG_A_S1b	18	528758	4.628800e-14	80	44	36	81.818184

	REF	\
20557	GTTTTTTTTTTTTTTTTAGTTTGAAC	
33962	T	
35494	CAGAGAGAGAGAGAGAGAGAGC	
57248	GTTTTTTTTTTTTTTTTTGAAAGA	
63072	CTAGTTTTTTTTTTTTTTTTAGTTTGAAC	
85859	TCTCAGAGAGAGAGAGAGAGAGAGC	
161975	TTTCCAAAAAAAAAAAAAAAGTAAAATAGAAAAGC	
214268	TCTAGTTTTTTTTTTTTTTTTAGTTTGAAC	
274642	TCAAACGTTTTTTTTTTTTTTTTTGAAAGAAATTGATTGCT	
280252	A	
406624	CAGAGAGAGAGAGAGAGAGAGC	
447613	A	
448420	ATTCTAGTTTTTTTTTTTTTTTTAGTTTGAACTCAAAA	
493008	AACGTTTTTTTTTTTTTTTTTGAAAGA	

	ALT	EFF	\
20557	GTTTTTTTTTATTTATAAGTTTGAAC	upstream_gene_variant	
33962	G	upstream_gene_variant	
35494	CAGAGAGAGAGAGAGAGAGC	upstream_gene_variant	
57248	GTTTTTTTTTTTTTTTTGAAAGA	upstream_gene_variant	
63072	TTAGATTTTTTTTTTTTTTTTAGTTTGAAC	upstream_gene_variant	
85859	ACTCAGAGAGAGAGAGAGAGAGAGAGC	upstream_gene_variant	
161975	ATTCTGAAAAAAAAAAAAAAAAGTAAAATAGAAAAGC	upstream_gene_variant	
214268	TCTAGTTTTTTTTTTTTCTATAGTTTGAAC	upstream_gene_variant	
274642	CCAATCGTTTTTTTTTTTTTTTTTGAAAGAAATTGATTGCT	upstream_gene_variant	
280252	G	upstream_gene_variant	
406624	CAGAGAGAAAAAGAGAGAGAGC	upstream_gene_variant	
447613	G	upstream_gene_variant	
448420	TTTTTAATTTTTTTTTTTTTTAGTTTGAACTCAAAA	upstream_gene_variant	
493008	ACCCTTTTTTTTTTTTTTTTTGAAAGA	upstream_gene_variant	

	IMPACT	AFF	MUT	\
20557	MODIFIER	MPN508	c.-2605_-2599delAAAAAAAinsTATAAAT	
33962	MODIFIER	MPN094	c.-2292T>G	
35494	MODIFIER	MPN109	c.-624_-623delAG	
57248	MODIFIER	MPN435	c.-3956delA	

```
63072   MODIFIER  MPN508  c.-2614_-2586delGTTCAAACTAAAAAAAAAAAAAAAACTAGi…
85859   MODIFIER  MPN109  c.-646_-622delTCTCAGAGAGAGAGAGAGAGAGAGCinsACTC…
161975  MODIFIER  MPN148  c.-97_-63delTTTCCAAAAAAAAAAAAAAAGTAAAATAGAAAAG…
214268  MODIFIER  MPN508                        c.-2604_-2602delAAAinsTAG
274642  MODIFIER  MPN435                    c.-3937_-3933delTTTGAinsATTGG
280252  MODIFIER  MPN504                                        c.-702A>G
406624  MODIFIER  MPN109                          c.-635_-633delGAGinsAAA
447613  MODIFIER  MPN504                                        c.-702A>G
448420  MODIFIER  MPN508  c.-2620_-2583delTTTTGAGTTCAAACTAAAAAAAAAAAAAAA…
493008  MODIFIER  MPN435                        c.-3939_-3937delCGTinsGGG

          ANN_TYPE  log2Frac Condition  annotation
20557   intergenic  6.256833         2  intergenic
33962   intergenic  5.643856         2  intergenic
35494   intergenic  5.569856         2  intergenic
57248   intergenic  5.184424         2  intergenic
63072   intergenic  5.643856         2  intergenic
85859   intergenic  5.998140         2  intergenic
161975  intergenic  6.185933         3  intergenic
214268  intergenic  6.075807         3  intergenic
274642  intergenic  6.058894         3  intergenic
280252  intergenic  5.321928         3  intergenic
406624  intergenic  5.606862     3IPTG  intergenic
447613  intergenic  4.836501     3IPTG  intergenic
448420  intergenic  6.266382     3IPTG  intergenic
493008  intergenic  6.354350     3IPTG  intergenic
```

[632]:

```
# For genes
plt.figure()
plt.subplot(2,1,1)
sns.swarmplot(x='IMPACT', y='FRAC', hue='PASS', data=subdf1[(subdf1['FRAC']>=5)␣
 ↪& (subdf1['ANN_TYPE']=='gene')])
plt.xticks(rotation=45, ha='right')

plt.subplot(2,1,2)
sns.swarmplot(x='IMPACT', y='FRAC', hue='PASS',data=subdf2[(subdf2['FRAC']>=5)␣
 ↪& (subdf2['ANN_TYPE']=='gene')])
plt.xticks(rotation=45, ha='right')


# For intergenic
plt.figure()
plt.subplot(2,1,1)
sns.swarmplot(x='IMPACT', y='FRAC', hue='PASS',data=subdf1[(subdf1['FRAC']>=5)␣
 ↪& (subdf1['ANN_TYPE']=='intergenic')])
plt.xticks(rotation=45, ha='right')
```

```
plt.subplot(2,1,2)
sns.swarmplot(x='IMPACT', y='FRAC', hue='PASS',data=subdf2[(subdf2['FRAC']>=5)␣
↪& (subdf2['ANN_TYPE']=='intergenic')])
plt.xticks(rotation=45, ha='right')
```

```
Canvas(toolbar=Toolbar(toolitems=[('Home', 'Reset original view', 'home', 'home'), ('Back', 'Ba
```

```
Canvas(toolbar=Toolbar(toolitems=[('Home', 'Reset original view', 'home', 'home'), ('Back', 'Ba
```

[632]:
```
(array([0, 1]), <a list of 2 Text xticklabel objects>)
```

[288]:
```
def plot_fixation(df):
    """ Plot to show the percentage of variants mapping to each type of␣
↪annotation """
    colord = {'intergenic':0, 'gene':1, 'essential':2, 'non-essential':3,␣
↪'cassette':4}
    pasind = {2:0, 3:1, 15:2, 18:3}
    rs = {}
    colors = []
    for k, v in Counter(list(df['POS'])).items():
        if v>2:
            rs[k] = [0.0,0.0,0.0,0.0]
            subdf = df[df['POS']==k]
            for passage in set(subdf['PASS']):
                rs[k][pasind[passage]] =␣
↪list(subdf[subdf['PASS']==passage]['FRAC'])
    return rs
dic  = plot_fixation(data1)
```

[327]:
```
rs = {}
columns = ['p2_2_S1', 'p2_2_S2', 'p2_2_S3', 'p3_D_S2', 'p3_D_S3', 'p15_F_S1',␣
↪'p15_F_S5',  'p3IPTG_A_S1', 'p3IPTG_A_S1b']
for n in columns:
    rs[n] = []
    for m in columns:
        rs[n].append(round(mannwhitneyu(data1[(data1['SAMPLE']==n) &␣
↪(data1['FRAC']>=1)]['FRAC'], data1[(data1['SAMPLE']==m) &␣
↪(data1['FRAC']>=1)]['FRAC'])[1], 8))
pvalues = pd.DataFrame.from_dict(rs, orient='index')
pvalues.columns=columns
pvalues
```

[327]:
```
              p2_2_S1   p2_2_S2   p2_2_S3   p3_D_S2   p3_D_S3  p15_F_S1  \
p2_2_S1           0.5  0.000000  0.000000  0.000000  0.000000  0.000000
```

p2_2_S2	0.0	0.500000	0.000000	0.000000	0.000000	0.304095
p2_2_S3	0.0	0.000000	0.499998	0.000000	0.368914	0.000000
p3_D_S2	0.0	0.000000	0.000000	0.499997	0.000000	0.000000
p3_D_S3	0.0	0.000000	0.368914	0.000000	0.499996	0.000000
p15_F_S1	0.0	0.304095	0.000000	0.000000	0.000000	0.500000
p15_F_S5	0.0	0.000000	0.000000	0.481911	0.000000	0.000000
p3IPTG_A_S1	0.0	0.000000	0.297108	0.000000	0.370263	0.000000
p3IPTG_A_S1b	0.0	0.000000	0.000005	0.000000	0.000963	0.000000

	p15_F_S5	p3IPTG_A_S1	p3IPTG_A_S1b
p2_2_S1	0.000000	0.000000	0.000000
p2_2_S2	0.000000	0.000000	0.000000
p2_2_S3	0.000000	0.297108	0.000005
p3_D_S2	0.481911	0.000000	0.000000
p3_D_S3	0.000000	0.370263	0.000963
p15_F_S1	0.000000	0.000000	0.000000
p15_F_S5	0.499997	0.000000	0.000000
p3IPTG_A_S1	0.000000	0.499991	0.008616
p3IPTG_A_S1b	0.000000	0.008616	0.499999

[352]:
```
def extracta(df, sample):
    rs = []
    subdf = df[df['SAMPLE']==sample].copy()
    fractions = {}
    for k, v in zip(subdf['POS'], subdf['FRAC']):
        if k in fractions:
            fractions[k].append(v)
        else:
            fractions[k] = [v]
    fractions = {k:np.mean(v) for k, v in fractions.items()}
    for i in range(1, len(genome_C5)+1):
        if i in fractions:
            if fractions[i]>=1:
                rs.append(i)
            else:
                rs.append(1)
    return np.array(rs)

def volcano():
    rs = {}
    for sample in set(data1[data1['PASS'].isin([3,18])]['SAMPLE']):
         rs[sample] = extracta(data1, sample)
    fcs = []
    pvals = []
    positions = []
    for i in range(1, len(genome_C5)+1):
        try:
```

```
            vals = np.array([rs['p3IPTG_A_S1b'][i], rs['p3IPTG_A_S1'][i],␣
↪rs['p3_D_S3'][i], rs['p3_D_S2'][i]])
            if len(vals[vals>1])==0:
                pass
            else:
                fc = np.log2(np.mean([rs['p3IPTG_A_S1b'][i],␣
↪rs['p3IPTG_A_S1'][i]]))-np.log2(np.mean([rs['p3_D_S3'][i],␣
↪rs['p3_D_S2'][i]]))
                pval = -1*np.log10(mannwhitneyu([rs['p3IPTG_A_S1b'][i],␣
↪rs['p3IPTG_A_S1'][i]], [rs['p3_D_S3'][i], rs['p3_D_S2'][i]])[1])
                fcs.append(fc)
                pvals.append(pval)
                positions.append(i)
        except:
            pass
    return fcs, pvals, positions
```

[353]:
```
f, pv, p  = volcano()
```

[357]:
```
plt.figure()
plt.scatter(f, pv)
plt.axhline(y=-1*np.log10(0.05), linestyle='--', color='r')
```

```
/home/smiravet/.local/lib/python3.6/site-packages/ipykernel_launcher.py:1:
RuntimeWarning: More than 20 figures have been opened. Figures created through
the pyplot interface (`matplotlib.pyplot.figure`) are retained until explicitly
closed and may consume too much memory. (To control this warning, see the
rcParam `figure.max_open_warning`).
  """Entry point for launching an IPython kernel.
```

```
Canvas(toolbar=Toolbar(toolitems=[('Home', 'Reset original view', 'home', 'home'), ('Back', 'Ba
```

[357]:
```
<matplotlib.lines.Line2D at 0x7fef2ff662b0>
```

[601]:
```
background = np.log2(data1[(data1['PASS']==3) & (data1['FRAC']>=1)]['FRAC'])
plt.figure()
sns.distplot(background)
plt.axvline(x=np.mean(background))
plt.axvline(x=np.percentile(background,99), color='r', linestyle='--')
selected = data1[data1['PASS']==18].copy()
#selected['pvalue'] = [mannwhitneyu(frac, background) for frac in␣
↪list(selected['FRAC'])]
```

```
/home/smiravet/.local/lib/python3.6/site-packages/ipykernel_launcher.py:2:
RuntimeWarning: More than 20 figures have been opened. Figures created through
the pyplot interface (`matplotlib.pyplot.figure`) are retained until explicitly
```

```
closed and may consume too much memory. (To control this warning, see the
rcParam `figure.max_open_warning`).
```

```
Canvas(toolbar=Toolbar(toolitems=[('Home', 'Reset original view', 'home', 'home'), ('Back', 'Ba
```

[250]:
```
new_dic = {}
for k, v in dic.items():
    a = np.array(v)
    try:
        if len(a[1])==2 and len(a[-1])==2:
            new_dic[k] = a
    except:
        pass
```

[270]:
```
x3, x3i, x15 = [[], [], []]
positions = []
for k, v in dic.items():
    a = np.array(v)
    try:
        if len(a[0])==2:
            if len(a[1])==2:
                x3.append(np.mean(a[1])/np.mean(a[0]))
            elif len(a[2])==2:
                x15.append(np.mean(a[2])/np.mean(a[0]))
            elif len(a[-1])==2:
                positions.append(k)
                x3i.append(np.mean(a[-1])/np.mean(a[0]))
            else:
                pass
    except:
        pass
```

[271]:
```
plt.figure()
plt.violinplot([x3, x15, x3i])
plt.xticks([1,2,3],['P3/P2', 'P15/P2', 'P3-IPTG/P2'])
plt.axhline(y=np.mean(x3)+2*np.std(x3), color='red', linestyle='--', label='95%␣
↪threshold P3/P2')
```

```
Canvas(toolbar=Toolbar(toolitems=[('Home', 'Reset original view', 'home', 'home'), ('Back', 'Ba
```

[271]:
```
<matplotlib.lines.Line2D at 0x7fef462b1e10>
```

[274]:
```
thr = np.mean(x3)+2*np.std(x3)
thr
```

[274]:
```
1.0411993871078709
```

[279]:
```
a = np.array(x3i)
b = np.array(positions)
```

[282]:
```
b[a>=thr]
```

[282]:
```
array([211633, 264869, 198159, 224676, 254989, 458314, 553466, 675401,
       767918, 807060])
```

1.7 3. Fixation of the mutations

In this section we evaluate which mutations increase their representation from passage 2 to 3 and comparing this ratio between non-treated and induced with IPTG samples.

[115]:
```
data1[(data1['ALTN']>10) & (data1['PASS']==3)]
```

[115]:
```
          SAMPLE  PASS     POS          QUAL  TOT  REFN  ALTN       FRAC  \
137985  p3_D_S2     3    6772  1.989160e-15  582   569    13   2.284710
138227  p3_D_S2     3    8563  0.000000e+00  523   512    11   2.148438
139095  p3_D_S2     3   15285  4.221290e-16  684   673    11   1.634472
139787  p3_D_S2     3   20319  2.260140e-14  599   588    11   1.870748
140828  p3_D_S2     3   28083  0.000000e+00  644   633    11   1.737757
…           …   …     …         …   …    …    …     …
267794  p3_D_S3     3  414743  2.170210e-15  172   161    11   6.832298
269396  p3_D_S3     3  442878  0.000000e+00  235   222    13   5.855856
274642  p3_D_S3     3  528755  8.763400e-14  140    84    56  66.666664
280845  p3_D_S3     3  629170  0.000000e+00  175   104    71  68.269234
286599  p3_D_S3     3  712979  0.000000e+00  228   216    12   5.555555

                                              REF  \
137985                              TCTTTAAGGAAAA
138227          AAACCAAAATAAACCAATGAAACAAGGCGTGT
139095                             CCCTAAAGAACAAGG
139787                              CCCGTTCCCGCGTG
140828                             AATCCACGGCCGTTT
…                                              …
267794                                          T
269396                                    TAGTGGT
274642  TCAAACGTTTTTTTTTTTTTTTTTGAAAGAAATTGATTGCT
280845          CTAGTTTTTTTTTTTTTTTTAGTTTGAACTCA
286599                              ACCCCCCCCGCTA

                                              ALT                     EFF  \
137985                              ACTTAAAGGAAAA        missense_variant
138227          AAACCAAAATAATCTATTGAAACAAGGCGTGT  initiator_codon_variant
139095                             TCCTGAAGAACAAGG        missense_variant
139787                              CCCGTTCCCGAGAG        missense_variant
```

```
140828                            AATCCACGGCTGTCT        missense_variant
…                                               …                       …
267794                                          A     synonymous_variant
269396                                    TGGAGGT       missense_variant
274642  CCAATCGTTTTTTTTTTTTTTTTTGAAAGAAATTGATTGCT    upstream_gene_variant
280845          TTAGCTTTTTTTTTTTTTTTTTAGTTTGAACTCA    upstream_gene_variant
286599                               ACCCCCCCGCTA    upstream_gene_variant

          IMPACT     AFF                                            MUT  \
137985  MODERATE  MPN004                              p.LeuPhe651HisLeu
138227       LOW  MPN006                                        p.Met1?
139095  MODERATE  MPN013                                    p.Lys100Glu
139787  MODERATE  MPN018                                    p.Val336Glu
140828  MODERATE  MPN022                              p.ArgPhe260CysLeu
…            …      …                                              …
267794       LOW  MPN347                                    p.Gly268Gly
269396  MODERATE  MPN370                                    p.Val544Glu
274642  MODIFIER  MPN435                    c.-3937_-3933delTTTGAinsATTGG
280845  MODIFIER  MPN508  c.-2617_-2586delTGAGTTCAAACTAAAAAAAAAAAAAAAACT…
286599  MODIFIER  MPN579                                    c.-4224delG

          ANN_TYPE
137985        gene
138227        gene
139095        gene
139787        gene
140828        gene
…              …
267794        gene
269396        gene
274642  intergenic
280845  intergenic
286599  intergenic

[68 rows x 15 columns]
```

[116]:

```
Canvas(toolbar=Toolbar(toolitems=[('Home', 'Reset original view', 'home', 'home'), ('Back', 'B
```

[116]: <matplotlib.axes._subplots.AxesSubplot at 0x7fa3e0a23a90>

[40]:

```python
def plot_fixation(df):

    subdf = df.groupby(['PASS', 'POS', 'ALT']).sum()
    return subdf
```

```
    positions = set([k for k, v in Counter(subdf['POS']).items() if v>=3])
    return df[df['POS'].isin(positions)]
```

[57]:
```
np.array([i for i in plot_fixation(snpcalls1).index])
```

[57]:
```
array([['2', '6', 'T'],
       ['2', '13', 'C'],
       ['2', '31', 'A'],
       …,
       ['18', '822913', 'TGCGTGAAA'],
       ['18', '822925', 'C'],
       ['18', '822931', 'CAAGTTCT']], dtype='<U73')
```

[196]:
```
plt.figure(figsize=(10,10))
sns.scatterplot(x='POS', y='FRAC', hue='PASS',␣
 ↪data=effect1[(effect1['IMPACT']=='HIGH') & (effect1['FRAC']>=1)])
```

```
Canvas(toolbar=Toolbar(toolitems=[('Home', 'Reset original view', 'home', 'home'), ('Back', 'Ba
```

[196]:
```
<matplotlib.axes._subplots.AxesSubplot at 0x7f1bd89350f0>
```

[80]:
```
plt.figure()
effect21['logFRAC'] = np.log2(effect21['FRAC'])
sns.violinplot(x='IMPACT', y='FRAC', hue='PASS', data=effect1)
```

```
/home/smiravet/.local/lib/python3.6/site-packages/ipykernel_launcher.py:1:
RuntimeWarning: More than 20 figures have been opened. Figures created through
the pyplot interface (`matplotlib.pyplot.figure`) are retained until explicitly
closed and may consume too much memory. (To control this warning, see the
rcParam `figure.max_open_warning`).
  """Entry point for launching an IPython kernel.
```

```
Canvas(toolbar=Toolbar(toolitems=[('Home', 'Reset original view', 'home', 'home'), ('Back', 'Ba
```

[80]:
```
<matplotlib.axes._subplots.AxesSubplot at 0x7f92f451a828>
```

[42]:
```
snpcalls1[snpcalls1['SAMPLE']=='p2_2_S1'].sort_values('FRAC')
```

[42]:
```
          POS                              REF  \
18482  566325                                C
18481  566311                                A
18485  566383                              GCC
18483  566361                                A
18476  566244                                A
…         …                              …
6416   195428  CAAAAAAAAAAAAAAAGTAAAATAGAAAAGC
```

```
17103  528761        GTTTTTTTTTTTTTTTTTGAAAGA
20563  629173      GTTTTTTTTTTTTTTTTAGTTTGAAC
4753   141270          CAGAGAGAGAGAGAGAGAGAGC
19735  602077                               T

                                                     ALT          QUAL  TOT  \
18482                                                  T  1.469330e-15  205
18481                                                  G  7.046470e-16  198
18485                                                CCT  0.000000e+00  181
18483                                                  G  0.000000e+00  172
18476                                                  G  1.167980e-15  171
…                                                    …             …    …
6416   CAAAATAAGAAAAAAAGTAAAATAGAAAAGC,CAAAAAAAAAAAAA…  0.000000e+00   24
17103  GTTTTTTTTTTTTTTTTGAAAGA,GTTTTTTTTTTTTTTTTTTGAAAGA  0.000000e+00   28
20563  GTTTTTTTTTATTTATAAGTTTGAAC,GTTTTTTTTTTTTTTTAGT…  1.383230e-14   30
4753       CAGAGAGAGAGAGAGAGAGC,CAGAGAGAGAGAGAGAGAGAGAGC  0.000000e+00   52
19735                                                  A  1.607630e+01    1

       REFN  ALTN       ALTS        FRAC    SAMPLE  P  \
18482   204     1          1    0.487805  p2_2_S1  2
18481   197     1          1    0.505051  p2_2_S1  2
18485   180     1          1    0.552486  p2_2_S1  2
18483   171     1          1    0.581395  p2_2_S1  2
18476   170     1          1    0.584795  p2_2_S1  2
…       …    …        …          …         … ..
6416     15     9    1,1,1,6   37.500000  p2_2_S1  2
17103    16    12        6,6   42.857143  p2_2_S1  2
20563    17    13  1,2,6,2,2   43.333333  p2_2_S1  2
4753     27    25       7,18   48.076923  p2_2_S1  2
19735     0     1          1  100.000000  p2_2_S1  2

                                                     EFF
18482  T|synonymous_variant|LOW|Gene_565549_566677|Ge…
18481  G|missense_variant|MODERATE|Gene_565549_566677…
18485  CCT|missense_variant|MODERATE|Gene_565549_5666…
18483  G|synonymous_variant|LOW|Gene_565549_566677|Ge…
18476  G|synonymous_variant|LOW|Gene_565549_566677|Ge…
…                                                    …
6416   CAAAATAAGAAAAAAAGTAAAATAGAAAAGC|upstream_gene_…
17103  GTTTTTTTTTTTTTTTTGAAAGA|upstream_gene_variant|…
20563  GTTTTTTTTTATTTATAAGTTTGAAC|upstream_gene_varia…
4753   CAGAGAGAGAGAGAGAGAGC|upstream_gene_variant|MOD…
19735  A|missense_variant|MODERATE|P02_orf1300|MPN489…

[27073 rows x 12 columns]
```

[46]:

[47]:
```
simplify_effect(snpcalls1, genome=20)
```

```
                                                                              ␣
↪---------------------------------------------------------------------------

      UnboundLocalError                         Traceback (most recent call␣
↪last)

      <ipython-input-47-d335a4bfad19> in <module>
    ----> 1 simplify_effect(snpcalls1, genome=20)


      <ipython-input-46-bf2fb86f217b> in simplify_effect(df, positions,␣
↪genome, modify_original, impact, mintot, minrefn, minaltn)
          7         annotations = correspondance(genome=genome,␣
↪positions=positions)
          8     else:
    ----> 9         annotations
         10     return annotations
         11     annotations = {v.qualifiers['label'][0]:[int(v.location.start),␣
↪int(v.location.end), '-'] for k, v in annotations.items() if 'label' in v.
↪qualifiers}


      UnboundLocalError: local variable 'annotations' referenced before␣
↪assignment
```

[45]:
```
# Figure


def plot_analysis(df):
    repro = [k for k, v in Counter(list(df.POS)).items() if v>=2]
    plt.figure()
    plt.subplot(3,3,1)
    sns.lineplot(x='P', y='FRAC', data=df[(df['POS'].isin(posE)) & (df['POS'].
↪isin(repro))], label='E')
    sns.lineplot(x='P', y='FRAC', data=df[(df['POS'].isin(posN)) & (df['POS'].
↪isin(repro))], label='NE')
    sns.lineplot(x='P', y='FRAC', data=df[(df['POS'].isin(cas1)) & (df['POS'].
↪isin(repro))], label='C')


plot_analysis(snpcalls1)
```

```
Canvas(toolbar=Toolbar(toolitems=[('Home', 'Reset original view', 'home', 'home'), ('Back', 'Ba
```

1.8 4. Substituion rate per base comparing cassette versus other distributions

To evaluate the selection in the cassette we compare the rate of substitutions per base occuring within the cassette, a set of essential and non-essential genes and with the general distribution observed at genome level. In this case we do not care about the fraction each variant is found but how many times a variant is found in the cassette versus the other distributions.

[11]:
```
# Show the ratio of mutation in cassette versus general
def mutation_rate(snpdf, segment, mintot=5, minrefn=3, minaltn=2):
    _df = snpdf[(snpdf['TOT']>=mintot) & (snpdf['REFN']>=minrefn) &␣
↪(snpdf['ALTN']>=minaltn)].copy()
    rs = {}
    for sample in set(_df.SAMPLE):
        total_len = 816394
        _df2 = _df[_df['SAMPLE']==sample].copy()
        NE = _df2[_df2['POS'].isin(posN)]
        ES = _df2[_df2['POS'].isin(posE)]
        TG = _df2[~_df2['POS'].isin(segment)]
        CS = _df2[_df2['POS'].isin(segment)]
        #total_len+=len(segment)
        rs[sample] = [sample, sample.split('_')[0],
                      int(sample.split('_')[0].replace('p', '').
↪replace('3IPTG', '18')),
                      TG.shape[0], ES.shape[0], NE.shape[0], CS.shape[0],
                      sum(TG['FRAC']), sum(ES['FRAC']), sum(NE['FRAC']),␣
↪sum(CS['FRAC'])]
    jj = pd.DataFrame.from_dict(rs, orient='index')
    jj.columns = ['SAMPLE', 'COND', 'P', 'T', 'E', 'N', 'C', 'TF', 'EF', 'NF',␣
↪'CF']
    jj['T%'] = 100*jj['T']/(total_len)
    jj['E%'] = 100*jj['E']/len(posE)
    jj['N%'] = 100*jj['N']/len(posN)
    jj['C%'] = 100*jj['C']/len(segment)
    return jj

def tp(df, order):
    rs = {}
    c=0
    for row, col in df.iterrows():
        rs[c] = [col['COND'],'Essential', col['E%'], col['EF']]
        rs[c+1] = [col['COND'],'Non-essential', col['N%'], col['NF']]
        rs[c+2] = [col['COND'],'Chromosome', col['T%'], col['TF']]
        rs[c+3] = [col['COND'],'Cassette', col['C%'], col['CF']]
        c+=4
    a = pd.DataFrame.from_dict(rs, orient='index')
```

```
    a.columns = ['Condition', 'Loci', 'Variation per base [%]', 'Accumulated␣
↪Fraction [%]']
    return orderdf(a, ordered_classes=order, col='Condition')
```

[32]:
```
mutrate11 = mutation_rate(snpcalls1, segment=cas1)
mutrate21 = mutation_rate(snpcalls2, segment=cas1)
mutrate22 = mutation_rate(snpcalls2, segment=cas2)
mutrate212 = mutation_rate(snpcalls2, segment=cas12)
```

[33]:
```
plt.close('all')
plt.figure(figsize=(15, 3))
c = 1
for text, df in zip(['C5 Par', 'C20 Par', 'C20 3b'], [mutrate11, mutrate21,␣
↪mutrate22]):
    plt.subplot(1,3,c)
    if text=='C5 Par':
        sns.barplot(x='Condition', y='Variation per base [%]', hue='Loci',␣
↪palette='mako', data=tp(df, ['p2','p3','p15', 'p3IPTG']))
    else:
        sns.barplot(x='Condition', y='Variation per base [%]', hue='Loci',␣
↪palette='mako', data=tp(df, ['p2','p3','p15']))
    plt.ylim(0, 15)
    c+=1
plt.tight_layout()
```

```
Canvas(toolbar=Toolbar(toolitems=[('Home', 'Reset original view', 'home', 'home'), ('Back', 'B
```

[27]:
```
from dnds_functions import evolution_of_sample, dnds
ttt = evolution_of_sample(effect1[effect1['IMPACT']=='HIGH'])
```

[40]:
```
effectC= simplify_effect(snpcalls1, cas1, minaltn=3)
effectE= simplify_effect(snpcalls1, posE, minaltn=3)
effectN= simplify_effect(snpcalls1, posN, minaltn=3)

tttC = evolution_of_sample(effectC)
tttE = evolution_of_sample(effectE)
tttN = evolution_of_sample(effectN)
```

[53]:
```
tttE
```

[53]:
```
{'p15_F_S1': [0.015,
  0.011,
  1.3636363636363638,
  0.015,
  0.011,
  1.3636363636363638],
```

```
'p3IPTG_A_S1': [0.418,
 0.265,
 1.5773584905660376,
 0.612,
 0.327,
 1.8715596330275228],
'p3_D_S3': [0.012, 0.0, '+', 0.012, 0.0, '+'],
'p2_2_S1': [0, 0, '/', 0, 0, '/'],
'p3IPTG_A_S1b': [0, 0, '/', 0, 0, '/'],
'p15_F_S5': [0, 0, '/', 0, 0, '/'],
'p2_2_S3': [0.009000000000000001,
 0.022,
 0.40909090909090917,
 0.009000000000000001,
 0.022,
 0.40909090909090917],
'p2_2_S2': [0.006, 0.0, '+', 0.006, 0.0, '+'],
'p3_D_S2': [0.03,
 0.06599999999999999,
 0.4545454545454546,
 0.03,
 0.06599999999999999,
 0.4545454545454546]}
```

[55]:
```
plt.figure()
x, y = [[], []]
for k, v in tttC.items():
    if type(v[-1])==float and type(tttE[k][-1])==float:
        x.append(tttE[k][-1])
        y.append(v[-1])
plt.scatter(x, y)
```

```
Canvas(toolbar=Toolbar(toolitems=[('Home', 'Reset original view', 'home', 'home'), ('Back', 'Ba
```

[55]:
```
<matplotlib.collections.PathCollection at 0x7f92ec6b1be0>
```

[307]:
```
plt.figure()
plt.subplot(2,2,1)
sns.countplot(x='SAMPLE', data=snpcalls1)
plt.xticks(rotation=45, ha='right')
plt.title('Variant count per sample')

plt.subplot(2,2,2)
sns.boxplot(x='SAMPLE', y='FRAC', data=snpcalls1)
plt.xticks(rotation=45, ha='right')
plt.title('Fraction distribution per sample')
```

```
plt.subplot(2,2,3)
sns.countplot(x='SAMPLE', data=snpcalls2)
plt.xticks(rotation=45, ha='right')
plt.title('Variant count per sample')

plt.subplot(2,2,4)
sns.boxplot(x='SAMPLE', y='FRAC', data=snpcalls2)
plt.xticks(rotation=45, ha='right')
plt.title('Fraction distribution per sample')

plt.tight_layout()
```

```
Canvas(toolbar=Toolbar(toolitems=[('Home', 'Reset original view', 'home', 'home'), ('Back', 'Ba
```

General exploration of the most representative variants:

[308]:

```
plt.figure(figsize=(15, 10))
plt.subplot(3,1,1)
sns.scatterplot(x='POS', y='FRAC', hue='SAMPLE',␣
↪data=snpcalls1[(snpcalls1['FRAC']>=5) & (snpcalls1['TOT']>=10) &␣
↪(snpcalls1['P']<=15) & (snpcalls1['FRAC']<100)])
plt.axvspan(565510, 572114, color='gray', alpha=0.2)
plt.xlabel('')
plt.ylabel('Fraction of variant reads [%]')
plt.xlim(1, 890000)

plt.subplot(3,1,2)
sns.scatterplot(x='POS', y='FRAC', hue='SAMPLE',␣
↪data=snpcalls1[(snpcalls1['FRAC']>=5) & (snpcalls1['TOT']>=10) &␣
↪(snpcalls1['P']>15) & (snpcalls1['FRAC']<100)])
plt.axvspan(565510, 572114, color='gray', alpha=0.2)
plt.xlabel('')
plt.ylabel('Fraction of variant reads [%]')
plt.xlim(1, 890000)

plt.subplot(3,1,3)
sns.scatterplot(x='POS', y='FRAC', hue='SAMPLE',␣
↪data=snpcalls2[(snpcalls2['FRAC']>=5) & (snpcalls2['TOT']>=10) &␣
↪(snpcalls2['FRAC']<100)])
plt.axvspan(cass_par[0], cass_par[1], color='gray', alpha=0.2)
plt.axvspan(cass_3b[0], cass_3b[1], color='gray', alpha=0.2)
plt.xlabel('genome position [bp]')
plt.ylabel('Fraction of variant reads [%]')
plt.xlim(1, 890000)

plt.savefig('./figures/supfig1.svg')
```

```
plt.savefig('./figures/supfig1.png')
```

```
Canvas(toolbar=Toolbar(toolitems=[('Home', 'Reset original view', 'home', 'home'), ('Back', 'Ba
```

```
[ ]:
```

```
[ ]:
```

2 Selection of variants

In this case we evaluate the fraction of each variant and how it is fixed along passages. As not all the samples are directly related, we consider the induced sample (IPTG) as 18. In this sense, if a mutation found in passages 2, 3 or 15 is found in 18 with a higher rate it implies that this variants are able to confere the capability to surpass the killswitch

```
[610]: def plot_evolution(df):
    positions = set(df[df['FRAC']>1].POS)
    plt.figure()
    plt.subplot(1,2,1)
    sns.lineplot(x='PASS', y='FRAC', hue='POS', data=df[df['POS'].
 ↪isin(positions)])
    plt.subplot(1,2,2)
    sns.boxplot(x='PASS', y='FRAC', data=df[df['POS'].isin(positions)])
```

```
[611]: plot_evolution(data1)
```

```
/home/smiravet/.local/lib/python3.6/site-packages/ipykernel_launcher.py:3:
RuntimeWarning: More than 20 figures have been opened. Figures created through
the pyplot interface (`matplotlib.pyplot.figure`) are retained until explicitly
closed and may consume too much memory. (To control this warning, see the
rcParam `figure.max_open_warning`).
  This is separate from the ipykernel package so we can avoid doing imports
until
```

```
Canvas(toolbar=Toolbar(toolitems=[('Home', 'Reset original view', 'home', 'home'), ('Back', 'Ba
```

```
        ␣
 ↪---------------------------------------------------------------------------

        KeyboardInterrupt                         Traceback (most recent call␣
 ↪last)

        <ipython-input-611-effa08ace5dc> in <module>
    ----> 1 plot_evolution(data1)
```

```
    <ipython-input-610-c53d1b5b5f59> in plot_evolution(df)
      3     plt.figure()
      4     plt.subplot(1,2,1)
----> 5     sns.lineplot(x='PASS', y='FRAC', hue='POS', data=df[df['POS'].isin(positions)])
      6     plt.subplot(1,2,2)
      7     sns.boxplot(x='PASS', y='FRAC', data=df[df['POS'].isin(positions)])


    /usr/local/lib/python3.6/dist-packages/seaborn/relational.py in lineplot(x, y, hue, size, style, data, palette, hue_order, hue_norm, sizes, size_order, size_norm, dashes, markers, style_order, units, estimator, ci, n_boot, sort, err_style, err_kws, legend, ax, **kwargs)
   1082         ax = plt.gca()
   1083
-> 1084     p.plot(ax, kwargs)
   1085
   1086     return ax


    /usr/local/lib/python3.6/dist-packages/seaborn/relational.py in plot(self, ax, kws)
    764         # Loop over the semantic subsets and draw a line for each
    765
--> 766         for semantics, data in self.subset_data():
    767
    768             hue, size, style = semantics


    /usr/local/lib/python3.6/dist-packages/seaborn/relational.py in subset_data(self)
    330
    331             if self.sort:
--> 332                 subset_data = sort_df(subset_data, ["units", "x", "y"])
    333
    334             if self.units is None:


    /usr/local/lib/python3.6/dist-packages/seaborn/utils.py in sort_df(df, *args, **kwargs)
     43     """Wrapper to handle different pandas sorting API pre/post 0.17."""
     44     try:
```

```
---> 45         return df.sort_values(*args, **kwargs)
     46     except AttributeError:
     47         return df.sort(*args, **kwargs)


/usr/local/lib/python3.6/dist-packages/pandas/core/frame.py in sort_values(self, by, axis, ascending, inplace, kind, na_position)
   5001
   5002         new_data = self._data.take(
-> 5003             indexer, axis=self._get_block_manager_axis(axis), verify=False
   5004         )
   5005


/usr/local/lib/python3.6/dist-packages/pandas/core/internals/managers.py in take(self, indexer, axis, verify, convert)
   1395         new_labels = self.axes[axis].take(indexer)
   1396         return self.reindex_indexer(
-> 1397             new_axis=new_labels, indexer=indexer, axis=axis, allow_dups=True
   1398         )
   1399


/usr/local/lib/python3.6/dist-packages/pandas/core/internals/managers.py in reindex_indexer(self, new_axis, indexer, axis, fill_value, allow_dups, copy)
   1265                     ),
   1266                 )
-> 1267                 for blk in self.blocks
   1268             ]
   1269


/usr/local/lib/python3.6/dist-packages/pandas/core/internals/managers.py in <listcomp>(.0)
   1265                     ),
   1266                 )
-> 1267                 for blk in self.blocks
   1268             ]
   1269


/usr/local/lib/python3.6/dist-packages/pandas/core/internals/blocks.py in take_nd(self, indexer, axis, new_mgr_locs, fill_tuple)
   1312
   1313         new_values = algos.take_nd(
```

```
   -> 1314             values, indexer, axis=axis, allow_fill=allow_fill,␣
↪fill_value=fill_value
      1315         )
      1316


       /usr/local/lib/python3.6/dist-packages/pandas/core/algorithms.py in␣
↪take_nd(arr, indexer, axis, out, fill_value, mask_info, allow_fill)
      1649     if is_extension_array_dtype(arr):
      1650         return arr.take(indexer, fill_value=fill_value,␣
↪allow_fill=allow_fill)
   -> 1651     elif is_datetime64tz_dtype(arr):
      1652         return arr.take(indexer, fill_value=fill_value,␣
↪allow_fill=allow_fill)
      1653     elif is_interval_dtype(arr):


       KeyboardInterrupt:
```

[182]:

```
annotations
```

[182]:

```
{'LacI4': [565549, 566678, '-'],
 'pS': [566678, 566721, '-'],
 'Par cassette': [566747, 567674, '-'],
 'cas9B': [567711, 571818, '-'],
 'pG64': [571818, 571934, '-'],
 'gRNA2': [571942, 572044, '-'],
 'protospacer12 (10 targets)': [572024, 572044, '-'],
 'p438': [572044, 572066, '-'],
 'IR-OR': [572088, 572114, '-'],
 'MTn insertion point': [572113, 572124, '-']}
```

[330]:

```
from Bio.Seq import Seq
genes_affected = {}
for k, v in annotations.items():
    if k in ['cas9B', 'LacI4']:
        if k=='cas9B':
            seq = Seq(genome_C5[v[0]:v[1]])
        else:
            seq = Seq(genome_C5[v[0]:v[1]])
        print(len(seq)/3)
        rvc = seq.reverse_complement()
        mrn = rvc.transcribe()
        prt = mrn.translate(table=4)
        genes_affected[k] = [seq, rvc, mrn, prt, len(prt)]
```

```
376.3333333333333
1369.0
```

```
/home/smiravet/.local/lib/python3.6/site-packages/Bio/Seq.py:2309:
BiopythonWarning: Partial codon, len(sequence) not a multiple of three.
Explicitly trim the sequence or add trailing N before translation. This may
become an error in future.
  BiopythonWarning)
```

[333]:
```
genes_affected['LacI4'][3][-10:]
```

[333]:
```
Seq('RLESGQ*R*V', HasStopCodon(ExtendedIUPACProtein(), '*'))
```

LacI4 has extra bases in the annotation, that makes it to be non-codonic. I will consider the first stop found (move the end 10 bases)...

[33]:
```
from Bio.Seq import Seq
genes_affected = {}
for k, v in annotations.items():
    if k in ['cas9B', 'LacI4']:
        if k=='cas9B':
            seq = Seq(genome_seq[v[0]:v[1]])
        else:
            seq = Seq(genome_seq[v[0]+10:v[1]])
        print(len(seq)/3)
        rvc = seq.reverse_complement()
        mrn = rvc.transcribe()
        prt = mrn.translate(table=4)
        genes_affected[k] = [seq, rvc, mrn, prt, len(prt)]
```

```
        ␣
↪---------------------------------------------------------------------------

        NameError                                 Traceback (most recent call␣
↪last)

        <ipython-input-33-84fcf2bf1dbc> in <module>
          1 from Bio.Seq import Seq
          2 genes_affected = {}
    ----> 3 for k, v in annotations.items():
          4     if k in ['cas9B', 'LacI4']:
          5         if k=='cas9B':


        NameError: name 'annotations' is not defined
```

[34]:
```
genes_affected['LacI4'][3][-10:]
```

```
                                                                              ␣
↪---------------------------------------------------------------------------

        KeyError                                  Traceback (most recent call␣
↪last)

        <ipython-input-34-2cf0e14076cd> in <module>
    ----> 1 genes_affected['LacI4'][3][-10:]


        KeyError: 'LacI4'
```

[ ]:
```
aa
```

3 Evaluate effect of the selected mutations

[35]:
```
snpcalls1
```

[35]:
```
          POS        REF        ALT          QUAL  TOT  REFN  ALTN      FRAC  \
0          70          C          T  4.927350e-15   60    58     2  3.333333
1         149          T          C  0.000000e+00   75    74     1  1.333333
2         150          A          T  2.263910e-15   69    68     1  1.449275
3         154          T          C  0.000000e+00   62    61     1  1.612903
4         176       TAAT       CAAC  3.420820e-15   58    57     1  1.724138
…         …          …          …             …  …    …     …        …
41166  822775      AACGT      GACGC  3.631530e-15  171   170     1  0.584795
41167  822787          A          G  1.152910e-15  175   173     2  1.142857
41168  822830          T          G  3.857310e-15  165   164     1  0.606061
41169  822867      TTTTT      CTTTC  0.000000e+00  172   171     1  0.581395
41170  822913  CGCGCGTAT  TGCGTGAAA  0.000000e+00  161   160     1  0.621118

           SAMPLE   P                                                EFF
0         p2_2_S1   2  T|upstream_gene_variant|MODIFIER|dnaN|MPN001|t…
1         p2_2_S1   2  C|upstream_gene_variant|MODIFIER|dnaN|MPN001|t…
2         p2_2_S1   2  T|upstream_gene_variant|MODIFIER|dnaN|MPN001|t…
3         p2_2_S1   2  C|upstream_gene_variant|MODIFIER|dnaN|MPN001|t…
4         p2_2_S1   2  CAAC|upstream_gene_variant|MODIFIER|dnaN|MPN00…
…             …  ..                                                …
41166  p3IPTG_A_S1b  18  GACGC|missense_variant|MODERATE|soj|MPN688|tra…
41167  p3IPTG_A_S1b  18  G|synonymous_variant|LOW|soj|MPN688|transcript…
41168  p3IPTG_A_S1b  18  G|missense_variant|MODERATE|soj|MPN688|transcr…
```

```
41169  p3IPTG_A_S1b  18  CTTTC|missense_variant|MODERATE|soj|MPN688|tra…
41170  p3IPTG_A_S1b  18  TGCGTGAAA|upstream_gene_variant|MODIFIER|K05_o…

[508440 rows x 11 columns]
```

[36]:

```
␣
↪---------------------------------------------------------------------------

    NameError                                 Traceback (most recent call␣
↪last)

    <ipython-input-36-96ce78c8031b> in <module>
      1 c = 1
      2 rs = {}
----> 3 for i, j in selected.iterrows():
      4     for eff in str(j.EFF).split(','):
      5         jjj = eff.split('|')


    NameError: name 'selected' is not defined
```

[184]:

```
selected
```

```
␣
↪---------------------------------------------------------------------------

    NameError                                 Traceback (most recent call␣
↪last)

    <ipython-input-184-19ff2ad0f5a8> in <module>
----> 1 selected


    NameError: name 'selected' is not defined
```

[38]:

```
selected_ann
```

```
␣
↪---------------------------------------------------------------------------
```

```
    NameError                                 Traceback (most recent call␣
↪last)

    <ipython-input-38-28fde1192669> in <module>
----> 1 selected_ann


    NameError: name 'selected_ann' is not defined
```

[39]:
```
selected_ann[selected_ann['IMPACT']=='HIGH']
```

```
      ␣
↪---------------------------------------------------------------------------

    NameError                                 Traceback (most recent call␣
↪last)

    <ipython-input-39-e70eb41dc56f> in <module>
----> 1 selected_ann[selected_ann['IMPACT']=='HIGH']


    NameError: name 'selected_ann' is not defined
```

4 Mutation rate study

[92]:
```
plt.close('all')
```

[ ]:
```

```

[44]:
```
pd.melt(mutrate1.sort_values('P')[[i for i in mutrate1.columns if '%' in i]])
```

```
      ␣
↪---------------------------------------------------------------------------

    NameError                                 Traceback (most recent call␣
↪last)

    <ipython-input-44-d63c0ec7e1ed> in <module>
----> 1 pd.melt(mutrate1.sort_values('P')[[i for i in mutrate1.columns if␣
↪'%' in i]])
```

```
NameError: name 'mutrate1' is not defined
```

[45]:
```python
plt.figure()
plt.boxplot([mutrate1['N%'], mutrate2['N%']])
```

```
Canvas(toolbar=Toolbar(toolitems=[('Home', 'Reset original view', 'home', 'home'), ('Back', 'Ba
```

```
␣
↪---------------------------------------------------------------------------

NameError                                 Traceback (most recent call␣
↪last)

<ipython-input-45-05ad25cb1d90> in <module>
      1 plt.figure()
----> 2 plt.boxplot([mutrate1['N%'], mutrate2['N%']])


NameError: name 'mutrate1' is not defined
```

[46]:
```python
plt.close('all')
plt.figure()
x, y = ['OUT%', 'IN%']
plt.plot([0,16],[0,16], c='grey', linestyle='--')
sns.scatterplot(x=x, y=y, data=mutrate1)
for i in range(mutrate1.shape[0]):
    plt.text(x=mutrate1[x][i]+0.2,y=mutrate1[y][i]+0.3,s=mutrate1.index[i],
             fontdict=dict(size=8))
sns.scatterplot(x=x, y=y, data=mutrate2)
c=0
for i in range(mutrate2.shape[0]):

    if c==0:
        plt.text(x=mutrate2[x][i]+0.2,y=mutrate2[y][i]+0.4,s=mutrate2.index[i],
                 fontdict=dict(size=10))
        c==1
    else:
        plt.text(x=mutrate2[x][i]+0.2,y=mutrate2[y][i]+0.2,s=mutrate2.index[i],
                 fontdict=dict(size=8))
        c==0
plt.xlabel('Genome Variant Rate [%]')
plt.ylabel('Cassette Variant Rate [%]')
plt.xlim(0,16)
plt.ylim(0,16)
```

```
Canvas(toolbar=Toolbar(toolitems=[('Home', 'Reset original view', 'home', 'home'), ('Back', 'Ba
```

```
␣
↪---------------------------------------------------------------------------

    NameError                                 Traceback (most recent call␣
↪last)

    <ipython-input-46-c03ce5ec3171> in <module>
      3 x, y = ['OUT%', 'IN%']
      4 plt.plot([0,16],[0,16], c='grey', linestyle='--')
----> 5 sns.scatterplot(x=x, y=y, data=mutrate1)
      6 for i in range(mutrate1.shape[0]):
      7     plt.text(x=mutrate1[x][i]+0.2,y=mutrate1[y][i]+0.3,s=mutrate1.
↪index[i],


    NameError: name 'mutrate1' is not defined
```

[47]:
```
from scipy.stats import wilcoxon, ttest_rel
from collections import Counter

wrs = {}
trs = {}
ns = dict(Counter(mutrate.P))
for p in set(mutrate.P):
    wrs[p] = wilcoxon(x=list(mutrate[mutrate['P']==p]['OUT%']),
                      y=list(mutrate[mutrate['P']==p]['IN%']))[1]
    trs[p] = ttest_rel(list(mutrate[mutrate['P']==p]['OUT%']),
                       list(mutrate[mutrate['P']==p]['IN%']))[1]
```

```
␣
↪---------------------------------------------------------------------------

    NameError                                 Traceback (most recent call␣
↪last)

    <ipython-input-47-8c5c8001c3c9> in <module>
      4 wrs = {}
      5 trs = {}
----> 6 ns = dict(Counter(mutrate.P))
      7 for p in set(mutrate.P):
      8     wrs[p] = wilcoxon(x=list(mutrate[mutrate['P']==p]['OUT%']),
```

```
NameError: name 'mutrate' is not defined
```

[48]:
```
ns, wrs, trs
```

```
↪---------------------------------------------------------------------------

    NameError                                 Traceback (most recent call
↪last)

    <ipython-input-48-5ec18e92d2c6> in <module>
----> 1 ns, wrs, trs


    NameError: name 'ns' is not defined
```

null hypothesis (same mut rate) cannot be rejected at a confidence level of 5%, only at early passages 2 and 3 seems to be limited

[49]:
```
snpcalls50
```

```
↪---------------------------------------------------------------------------

    NameError                                 Traceback (most recent call
↪last)

    <ipython-input-49-bf7c51358a6e> in <module>
----> 1 snpcalls50


    NameError: name 'snpcalls50' is not defined
```

5 Plot variations

[50]:
```
cass_par
```

[50]:
```
[565510, 572124]
```

[51]:
```
selected
```

```
␣
↪---------------------------------------------------------------------------

    NameError                                 Traceback (most recent call␣
↪last)

    <ipython-input-51-19ff2ad0f5a8> in <module>
  ----> 1 selected


    NameError: name 'selected' is not defined
```

[52]:
```
from reportlab.lib import colors
from reportlab.lib.units import cm
from Bio.Graphics import GenomeDiagram


def plot_variations(genbank_file, coords, variations):
    record = SeqIO.read(genbank_file, "genbank")

    gd_diagram = GenomeDiagram.Diagram(record.id)
    gd_track_for_features = gd_diagram.new_track(1, name="Annotated Features")
    gd_feature_set = gd_track_for_features.new_set()

    for feature in record.features:
        if feature.location.start>=coords[0] and feature.location.
↪start<=coords[1] and feature.type!='Polymorphism':
            print(feature.type, feature.qualifiers['label'], feature.location)
            feature.location.strand=-1
            #if feature.type != "gene":
                # Exclude this feature
            #    continue
            #if len(gd_feature_set) % 2 == 0:
            #    color = colors.blue
            if feature.type=='CDS':
                color = colors.lightblue
                gd_feature_set.add_feature(
                    feature, sigil="ARROW", arrowshaft_height=1.0, color=color,␣
↪height=0.2, label=True, label_size=14, label_angle=0
                )
            # else:
            #     color = colors.lightblue
            #     gd_feature_set.add_feature(
            #         feature, sigil="BOX", color=color, height=0.1,␣
↪label=True, label_size=14, label_angle=10)
```

```
    colordic = {'stop': colors.firebrick, 'frameshift':colors.red, 'missense':
↪colors.orange}
    done = []

    for pos, eff in zip(variations.POS, variations.EFF):
        if pos not in done:
            effi = eff.split('|')[1].split('_')[0]
            color = colordic[effi]
            feature = SeqFeature(FeatureLocation(pos, pos+1))
            gd_feature_set.add_feature(
                    feature,
                    color=color,
                    name='  '+effi,
                    label=True,
                    label_size=15,
                    size=20
                    #label_color=color,
                )
            done.append(pos)
            for i in range(pos-30, pos+30):
                done.append(i)
    gd_diagram.draw(format="linear", pagesize="A5", fragments=1, start=565510,␣
↪end=572124)
    gd_diagram.write("plasmid_linear_nice.svg", "SVG")
```

[53]:
```
plot_variations('./data/C5_refSeq.gb', coords=[565549,571818],␣
↪variations=selected)
```

```
     ␣
↪---------------------------------------------------------------------------

     NameError                                 Traceback (most recent call␣
↪last)

     <ipython-input-53-3c5b915f50d3> in <module>
  ----> 1 plot_variations('./data/C5_refSeq.gb', coords=[565549,571818],␣
↪variations=selected)


     NameError: name 'selected' is not defined
```

[653]:
```

```

[637]:
```
raw_seq, new_seq = get_new_sequence(seq_record5, 'LacI4', pos=[565576, 'AGGC',␣
↪'GGGT'])
yyy = dnds(raw_seq, new_seq)
```

```
268.6666666666668
```

[654]:
```

```

[648]:
```
effect1[effect1['POS']==571815]
```

[648]:
```
        SAMPLE  PASS     POS  QUAL  TOT  REFN  ALTN      FRAC         REF  \
2140  p3_D_S2     3  571815   0.0  674   668     6  0.898204  CCAGATGTAA

             ALT         EFF IMPACT    AFF          MUT
2140  TCTGTTGTTT  start_lost   HIGH  cas9B  p.LeuAsp1?
```

[656]:
```
for k, v in ttt.items():
    print(k, v[-1])
```

```
p3IPTG_A_S1 2.404754044239023
p3_D_S2 1.7427616926503313
p15_F_S1 2.3935483870967738
p15_F_S5 3.4130506790327924
p3IPTG_A_S1b 2.6608581600149894
p3_D_S3 2.117820324005891
p2_2_S3 2.3640178337267255
p2_2_S1 2.127371273712737
p2_2_S2 1.7000000000000002
```

[620]:
```

```

[56]:
```
df = pd.read_csv(, header=60, sep='\t')
df2 = df[(df['INFO'].str.contains('CIGAR=1X;'))].sample(n=150, random_state=1).
↪copy()
```

```
  File "<ipython-input-56-9a8f373c509c>", line 1
    df = pd.read_csv(, header=60, sep='\t')
                     ^
SyntaxError: invalid syntax
```

[57]:
```
pn, ps, pns, dn, ds, dns = 0, 0, 0, 0, 0, 0
for item in sorted(output[chromosome]):
    raw_seq, new_seq = get_new_sequence(args.reference, chromosome,␣
↪output[chromosome][item][0].qualifiers[
```

```
        'locus_tag'][0], output[chromosome][item][1])
    newpn, newps, newdn, newds = dnds(raw_seq, new_seq)
    pn += newpn
    ps += newps
    dn += newdn
    ds += newds
if pn == 0 and ps == 0:
    pns, dns = '/', '/'
elif ps == 0:
    pns, dns = '+', '+'
elif pn == 0:
    pns, dns = '-', '-'
else:
    pns, dns = pn / ps, dn / ds
print args.query, chromosome, pn, ps, pns, dn, ds, dns
```

```
  File "<ipython-input-57-012244273d6f>", line 18
    print args.query, chromosome, pn, ps, pns, dn, ds, dns
             ^
SyntaxError: Missing parentheses in call to 'print'. Did you mean print(args.
↪query, chromosome, pn, ps, pns, dn, ds, dns)?
```

[58]:
```
for records in SeqIO.parse(, "genbank"):
    print(/)
    raw_seq, new_seq = get_new_sequence('./data/C5_refSeq.gb', 'C5',␣
↪output[chromosome][item][0].qualifiers[
        'locus_tag'][0], output[chromosome][item][1])
```

```
  File "<ipython-input-58-9fd2afaa1715>", line 1
    for records in SeqIO.parse(, "genbank"):
                               ^
SyntaxError: invalid syntax
```

[ ]:
```
```

[ ]:
```
```

Suppl. Fig. 1 – uncropped WBs

Primary Ab:

A) mouse monoclonal M2 anti-Flag (Sigma F1804, 1:2000 dilution) Ab

B) mouse monoclonal V5-10 anti-V5 (Sigma V8012, 1:200 dilution) Ab

C) mouse monoclonal 0.T.81 anti-LacI (abcom 33832, 1:500 dilution) Ab

D) mouse monoclonal 9E10 anti-cMyc (Sigma M4439, 1:2000 dilution) Ab

Secondary Ab:

Polyclonal anti-mouse IgG (Jackson Immune Research 515-035-003, 1:5000 dilution) Ab conjugated to horseradish peroxidase

A

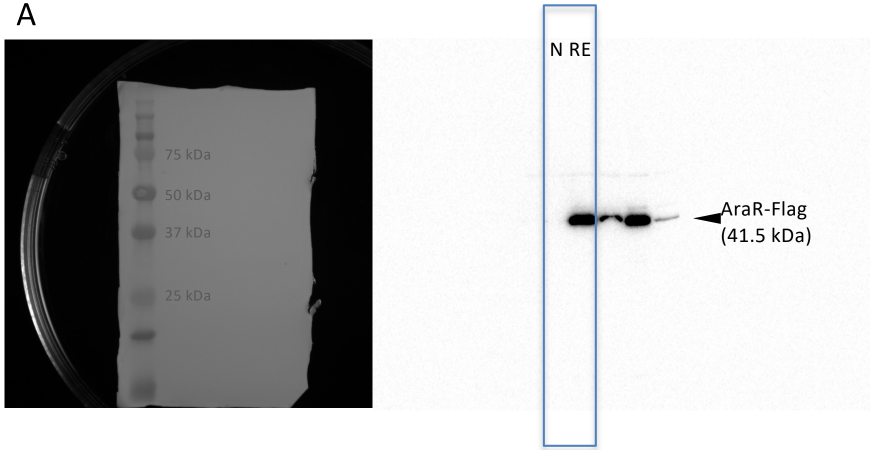

B

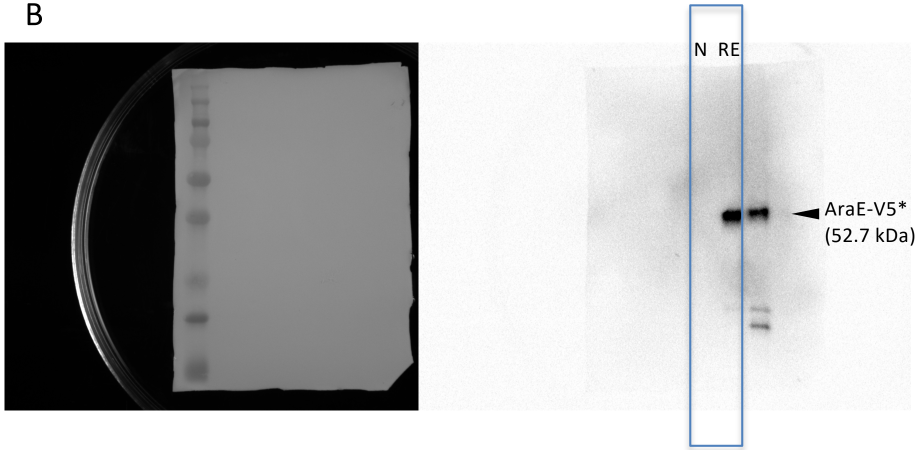

C

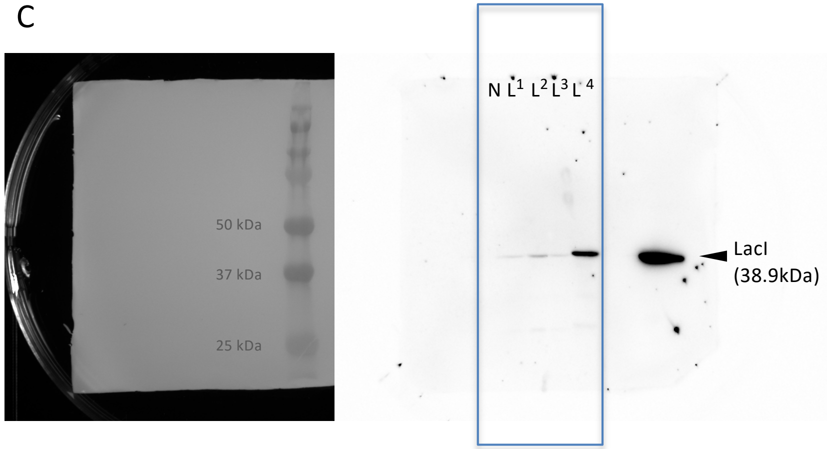

D

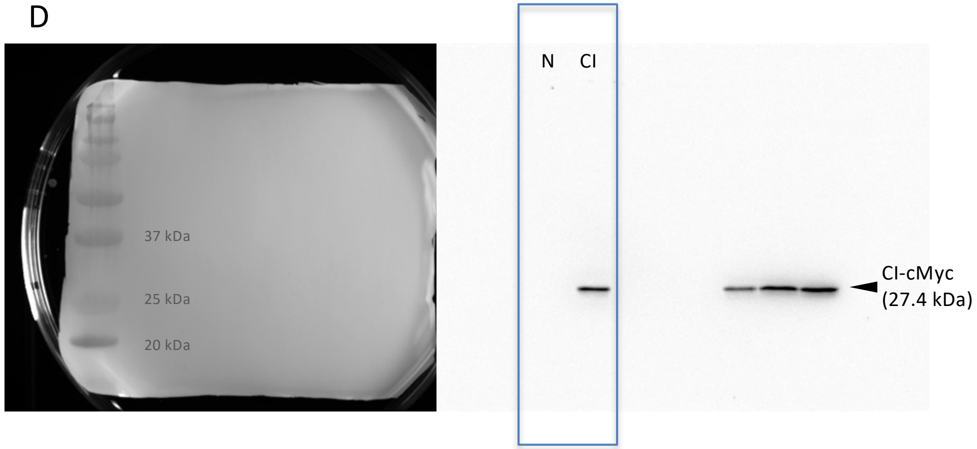

Suppl. Fig. 2B – uncropped WBs

Primary Ab: mouse monoclonal 8C5.5 anti-mCherry (BioLegend 677702, 1:1000 dilution) Ab

Secondary Ab: Polyclonal anti-mouse IgG (Jackson Immune Research 515-035-003, 1:5000 dilution) Ab conjugated to horseradish peroxidase

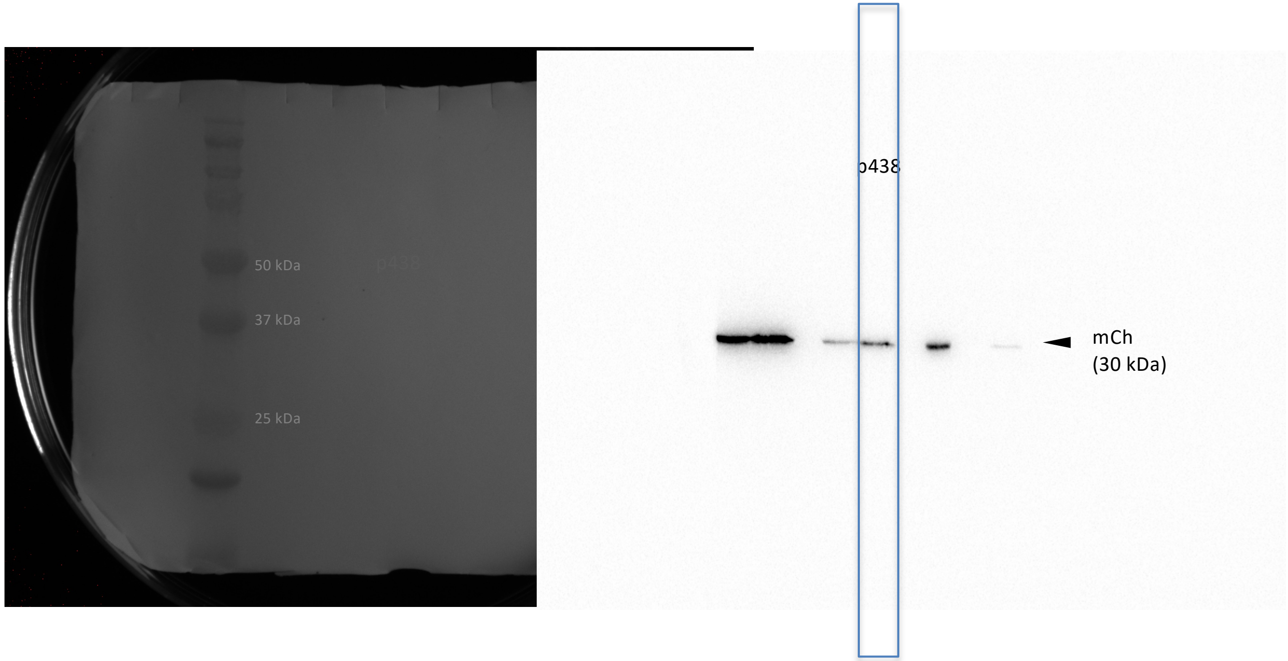

Suppl. Fig. 6B – uncropped WBs

Primary Ab: mouse monoclonal V5-10 anti-V5 (Sigma V8012, 1:200 dilution) Ab
Secondary Ab: Polyclonal anti-mouse IgG (Jackson Immune Research 515-035-003, 1:5000 dilution) Ab conjugated to horseradish peroxidase

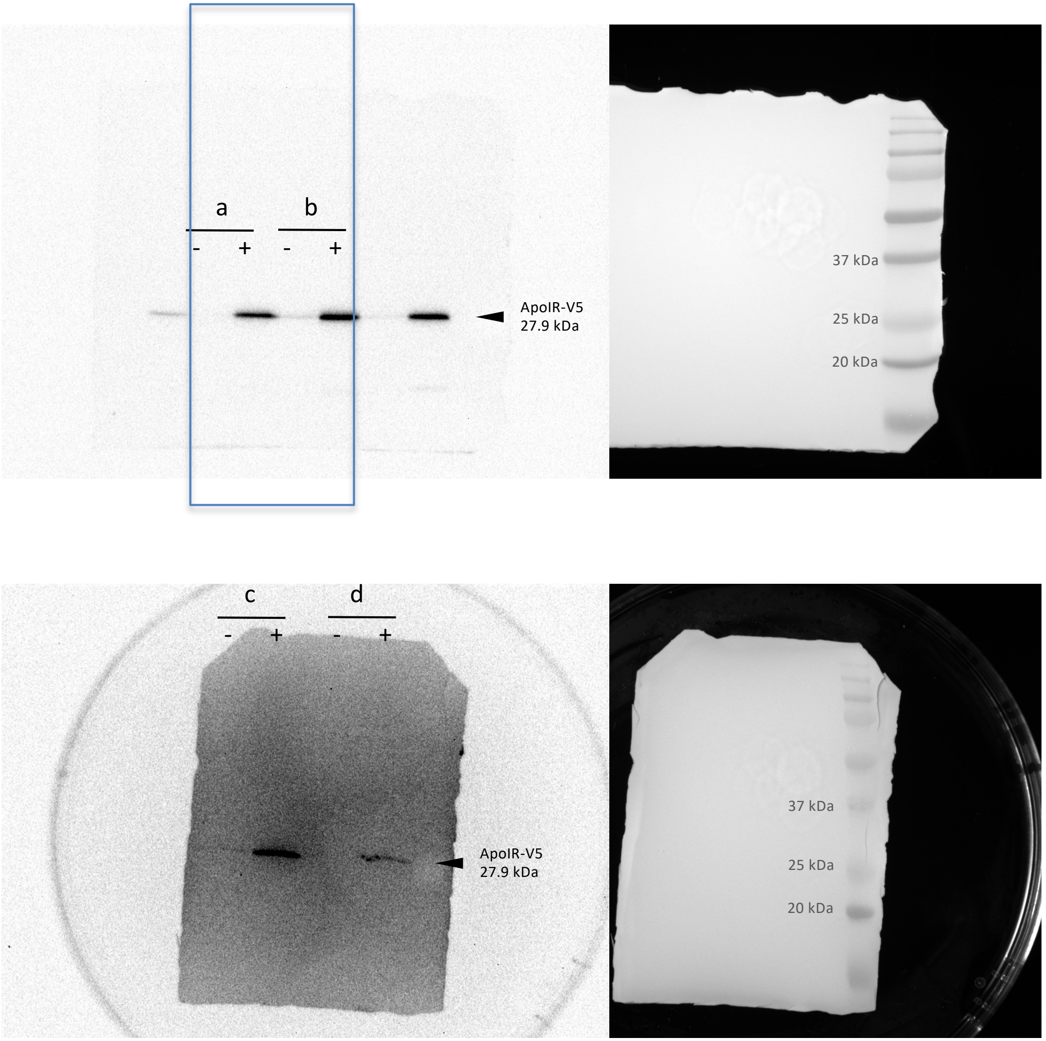

Suppl. Fig. 9 – uncropped DNA gel and WB

C) DNA gel

D) WB

Primary Ab: rabbit polyclonal anti-TetR (Sigma T0951, 1:1000 dilution)Ab

Secondary Ab: polyclonal anti-rabbit IgG (Jackson Immune Research 111-035-003, 1:5000 dilution) Ab conjugated to horseradish peroxidase

C

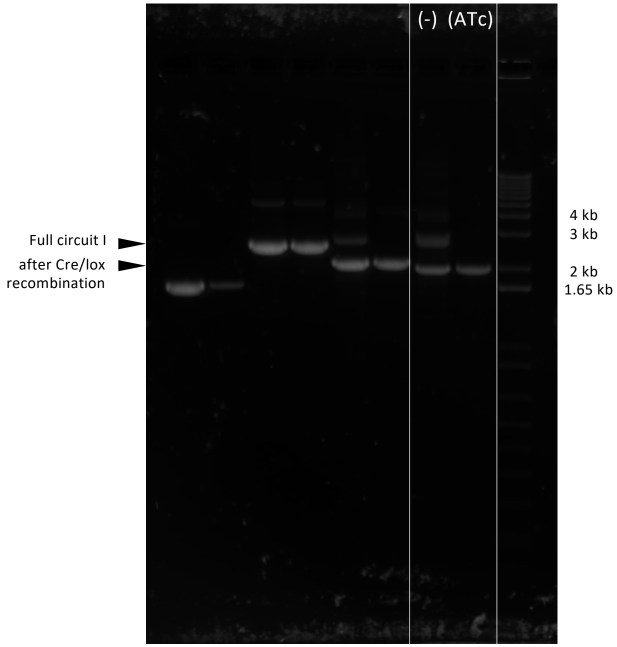

D

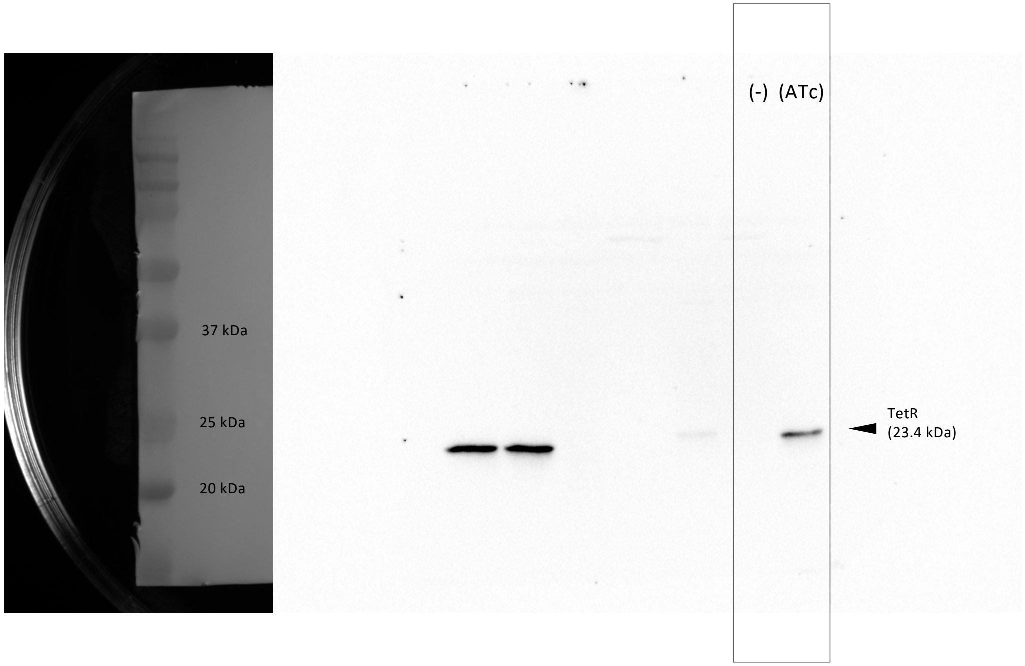

Suppl. Fig. 10 – uncropped DNA gel

C

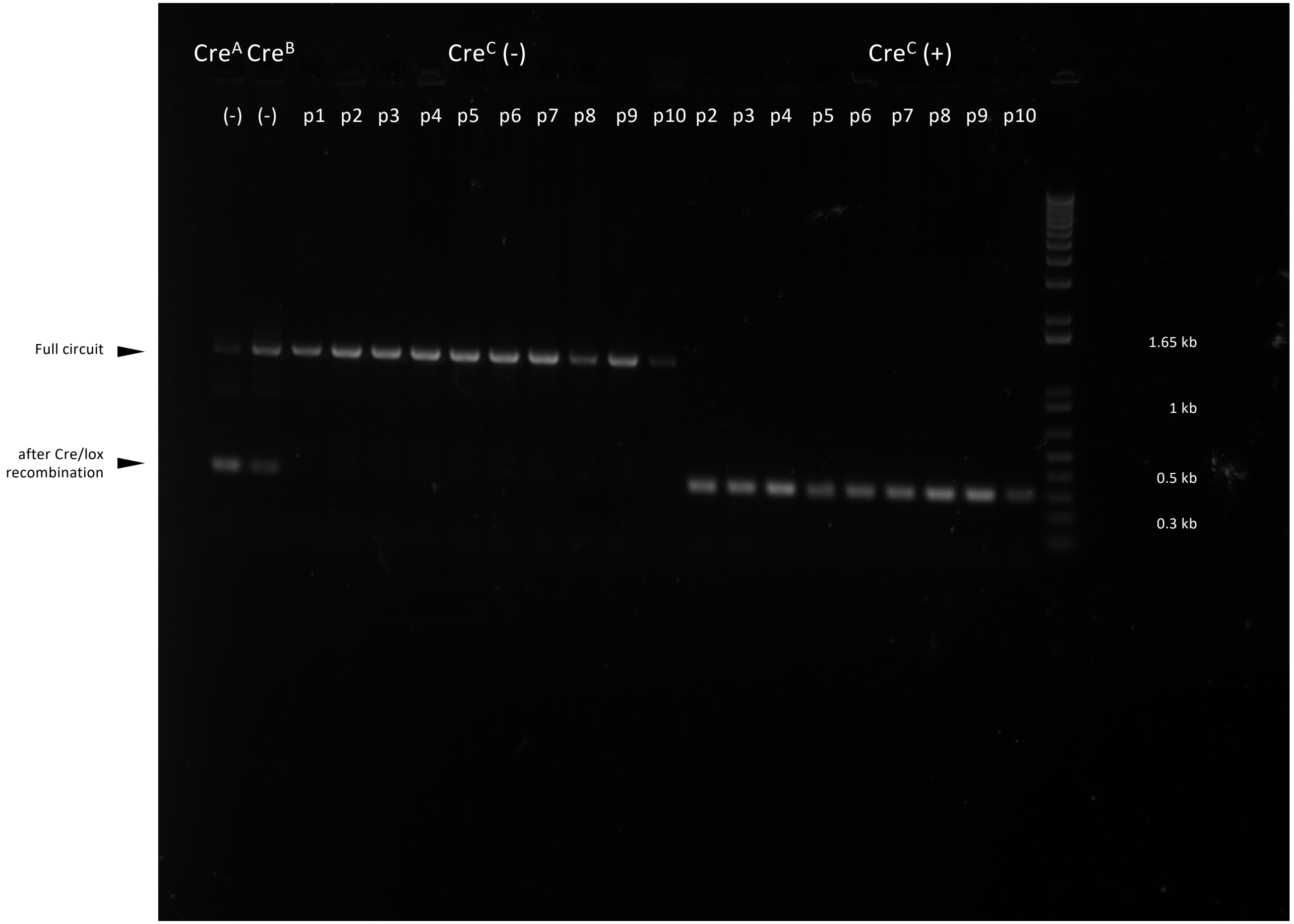

Suppl. Fig. 10 – uncropped WBs

D) Primary Ab: mouse monoclonal 7.23 anti-Cre recombinase (BioLegend 900901, 1:500 dilution)
Secondary Ab: Polyclonal anti-mouse IgG (Jackson Immune Research 515-035-003, 1:5000 dilution) Ab conjugated to horseradish peroxidase

E) Primary Ab:
Up) rabbit polyclonal anti-TetR (Sigma T0951, 1:1000 dilution)Ab
Mid) mouse monoclonal 9E10 anti-cMyc (Sigma M4439, 1:2000 dilution)
Low) mouse monoclonal 8C5.5 anti-mCherry (BioLegend 677702, 1:1000 dilution) Ab
Secondary Ab: Polyclonal anti-mouse IgG (Jackson Immune Research 515-035-003, 1:5000 dilution) or Polyclonal anti-rabbit IgG (Jackson Immune Research 111-035-003, 1:5000 dilution) Ab conjugated to horseradish peroxidase

E (up)

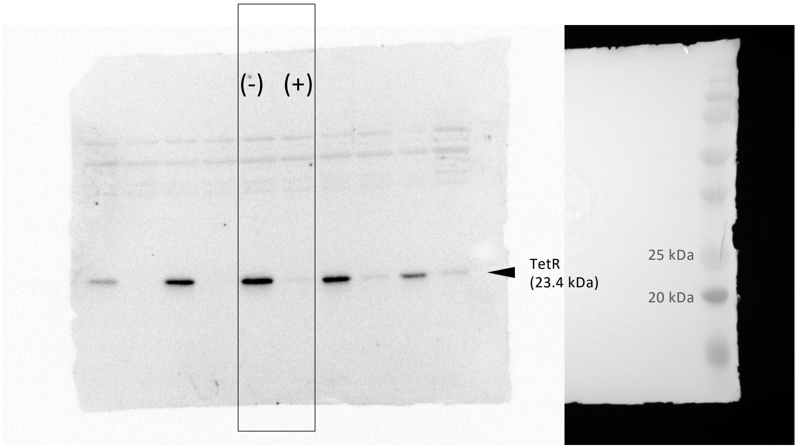

D

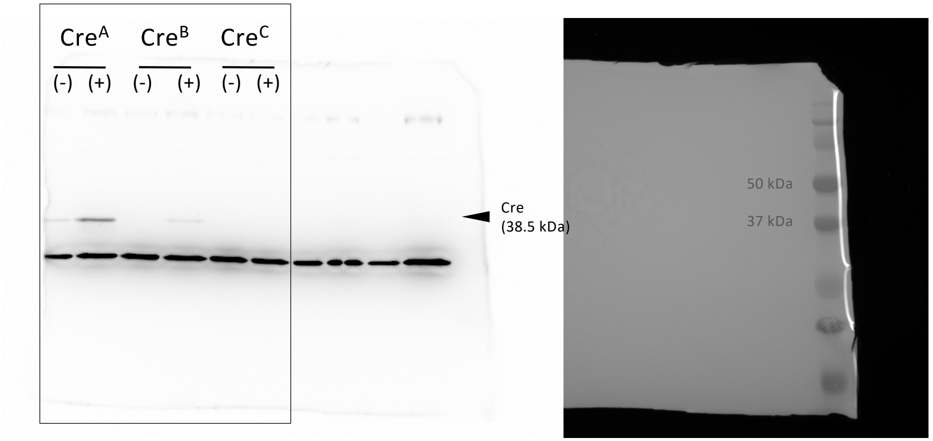

E (mid)

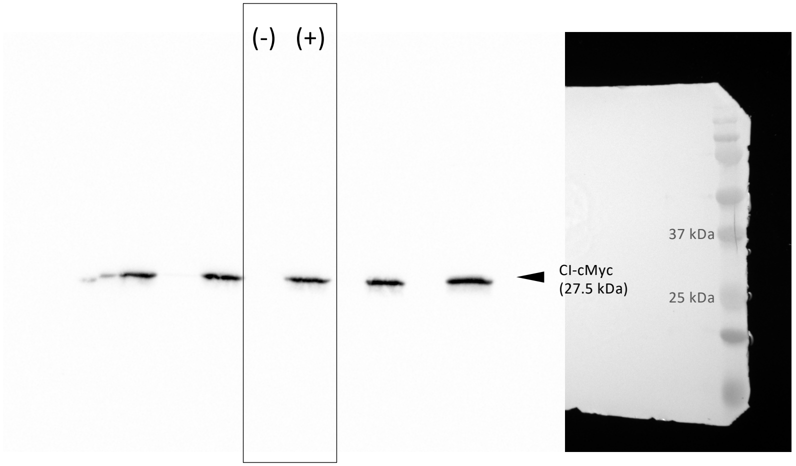

E (low)

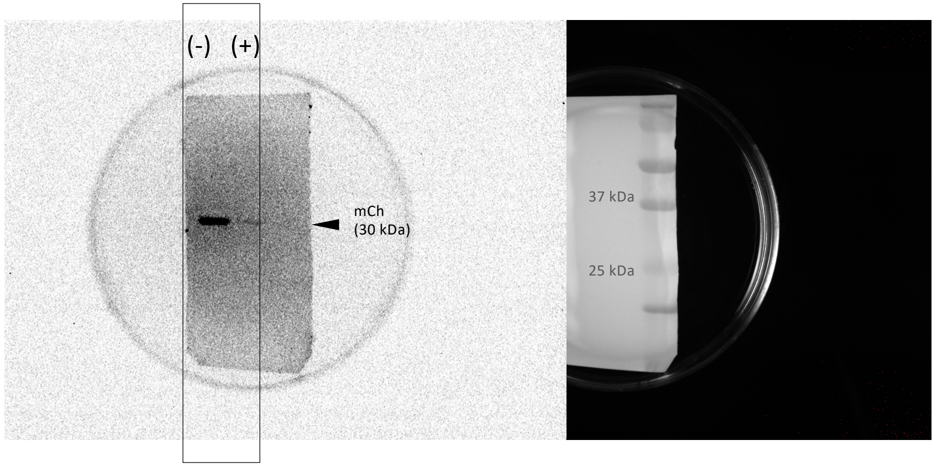

Supplement: Supplementary file 1 — Supplementary Information [file 41467_2022_29574_MOESM1_ESM.pdf]
