# Supplementary material for: A genetic toolkit and gene switches to limit Mycoplasma growth for biosafety applications
Source: Nat Commun. 2022 Apr 7;13:1910. doi: 10.1038/s41467-022-29574-0 (PMC8991246; doi:10.1038/s41467-022-29574-0)
Supplement: Supplementary file 2 — Reporting Summary [file 41467_2022_29574_MOESM2_ESM.pdf]

nature portfolio

Corresponding author(s): Dr. Mark Isalan

Last updated by author(s): 16/03/2022

Reporting Summary

Nature Portfolio wishes to improve the reproducibility of the work that we publish. This form provides structure for consistency and transparency in reporting. For further information on Nature Portfolio policies, see our Editorial Policies and the Editorial Policy Checklist.

Statistics

For all statistical analyses, confirm that the following items are present in the figure legend, table legend, main text, or Methods section.

n/a	Confirmed	
☐	☒	The exact sample size (*n*) for each experimental group/condition, given as a discrete number and unit of measurement
☐	☒	A statement on whether measurements were taken from distinct samples or whether the same sample was measured repeatedly
☐	☒	The statistical test(s) used AND whether they are one- or two-sided<br>*Only common tests should be described solely by name; describe more complex techniques in the Methods section.*
☐	☒	A description of all covariates tested
☐	☒	A description of any assumptions or corrections, such as tests of normality and adjustment for multiple comparisons
☐	☒	A full description of the statistical parameters including central tendency (e.g. means) or other basic estimates (e.g. regression coefficient) AND variation (e.g. standard deviation) or associated estimates of uncertainty (e.g. confidence intervals)
☐	☒	For null hypothesis testing, the test statistic (e.g. *F*, *t*, *r*) with confidence intervals, effect sizes, degrees of freedom and *P* value noted<br>*Give P values as exact values whenever suitable.*
☒	☐	For Bayesian analysis, information on the choice of priors and Markov chain Monte Carlo settings
☒	☐	For hierarchical and complex designs, identification of the appropriate level for tests and full reporting of outcomes
☐	☒	Estimates of effect sizes (e.g. Cohen's *d*, Pearson's *r*), indicating how they were calculated

Our web collection on statistics for biologists contains articles on many of the points above.

Software and code

Policy information about availability of computer code

Data collection	A Tecan infinite 200Pro plate reader was use for the time-course experiments and fluorimetries.<br>A ChemiDoc XRS+ Gel Imaging System (BioRad) was used for western blot imaging.<br>Snippy (https://github.com/tseemann/snippy, under license GPL v2, was used to extract SNP information from the sequencing raw files. Rest of code used was developed with Python 3 and can be found at https://github.com/CRG-CNAG/killswitch.
Data analysis	Codes used were developed with Python 3.6.9 and 3.4.4, and R 4.0.0. Codes can be found at https://github.com/CRG-CNAG/killswitch. Open source packages were used to work with biological sequences, processing, statistical analysis and plotting, including: pandas, numpy, seaborn, matplotlib, collections, cobrapy v. 0.5.11, biopython and Growthcurver (R) v. 0.3.1. For mapping raw sequencing reads to the reference genomes we use the bwa-short mode from Burrows-Wheeler Aligner (BWA) and freebayes program to call for sequence variants. This was run in the conda environment provided by the pipeline snippy. Models, analysis and data availability complie with the (FAIR) Data Management policies of the NWO, The Netherlands.

For manuscripts utilizing custom algorithms or software that are central to the research but not yet described in published literature, software must be made available to editors and reviewers. We strongly encourage code deposition in a community repository (e.g. GitHub). See the Nature Portfolio guidelines for submitting code & software for further information.

Data

Policy information about availability of data

All manuscripts must include a data availability statement. This statement should provide the following information, where applicable:

- Accession codes, unique identifiers, or web links for publicly available datasets
- A description of any restrictions on data availability
- For clinical datasets or third party data, please ensure that the statement adheres to our policy

The sequencing data generated in this study have been deposited in the ArrayExpress database at EMBL-EBI, under accession number E-MTAB-10981 (https://www.ebi.ac.uk/arrayexpress/experiments/E-MTAB-10981/). The source and processed data for all fluorimetries, time courses, E.F. and qPCR generated in this study are provided with this paper as Supplementary files.

Field-specific reporting

Please select the one below that is the best fit for your research. If you are not sure, read the appropriate sections before making your selection.

☒ Life sciences ☐ Behavioural & social sciences ☐ Ecological, evolutionary & environmental sciences

For a reference copy of the document with all sections, see nature.com/documents/nr-reporting-summary-flat.pdf

Life sciences study design

All studies must disclose on these points even when the disclosure is negative.

Sample size	No sample-size calculations were made. Sample sizes were decided based on the literature and prior experience in our laboratory. We chosen a minimum of n=3 bioreplicates, except n=2 in the case of the whole-genome sequencing analysis. We determined this to be sufficient considering the low observed variability.
Data exclusions	No data were excluded from the analysis.
Replication	All analyses were performed in parallel with three bioreplicate samples and statistical analyses were performed taking them into consideration. Experiments were repeated at least twice. All replication attempts were successful.
Randomization	Randomization was not relevant for this study since huge differences between treated and untreated mycoplasma samples were observed.
Blinding	All data collection in analyses was performed without labels. Sample names were only considered at the end to discuss the results. Also, all the bioinformatic analysis was done in a blind manner without knowing beforehand the type of each sample as data collection and data analysis was performed by different people. Sample names are only considered at the final steps of visualization and comparative between groups.

Reporting for specific materials, systems and methods

We require information from authors about some types of materials, experimental systems and methods used in many studies. Here, indicate whether each material, system or method listed is relevant to your study. If you are not sure if a list item applies to your research, read the appropriate section before selecting a response.

Materials & experimental systems

n/a	Involved in the study	
☐	☒	Antibodies
☒	☐	Eukaryotic cell lines
☒	☐	Palaeontology and archaeology
☒	☐	Animals and other organisms
☒	☐	Human research participants
☒	☐	Clinical data
☒	☐	Dual use research of concern

Methods

n/a	Involved in the study	
☒	☐	ChIP-seq
☒	☐	Flow cytometry
☒	☐	MRI-based neuroimaging

Antibodies

Antibodies used	Primary antibodies used in western blotting: Mouse monoclonal antibody clone 8C5.5 anti-mCherry from BioLegend cat. #677702; Mouse monoclonal antibody clone M2 anti-Flag from Sigma cat. #F1804; Mouse monoclonal antibody clone V5-10 anti-V5 from Sigma cat. #V8012; Mouse monoclonal antibody clone 0.T.81 anti-LacI from abcom cat. #33832; Mouse monoclonal antibody clone 9E10 anti-cMyc from Sigma cat. #M4439; Rabbit polyclonal antibody anti-TetR from Sigma cat. #T0951; Mouse monoclonal antibody clone 7.23 anti-Cre recombinase from BioLegend cat. #900901; Mouse monoclonal antibody clone 7A9 anti-Cas9 from BioLegend cat.

#844301; And, as secondary antibodies: Polyclonal sheep anti-mouse IgG from Jackson Immune Research cat. #515-035-003 and Polyclonal goat anti-rabbit IgG from Jackson Immune Research cat. #111-035-003, both conjugated to horseradish peroxidase.

Validation

Primary antibodies used in this study are against different heterologous proteins expressed in Mycoplasma. All antibodies were also tested against WT Mycoplasma extracts with negative results, shown in the corresponding blots included in the manuscript. All commercial primary antibodies used have been validated for use in western blot as stated on the corresponding manufactures product pages, as follows:

a) Mouse monoclonal antibody clone 8C5.5 anti-mCherry from BioLegend cat. #677702; Applications: WB - Quality tested IF, IP – Validated; Recommended Usage: Each lot of this antibody is quality control tested by Western blotting. For Western blotting, the suggested use of this reagent is 1.0 - 2.5 µg per ml. For immunofluorescence microscopy, a concentration range of 1-5 µg per ml is recommended. For immunoprecipitation, the suggested use of this reagent is 2-10 µg per ml. It is recommended that the reagent be titrated for optimal performance for each application.

b) Mouse monoclonal antibody clone M2 anti-Flag from Sigma cat. #F1804; Monoclonal ANTI-FLAG M2 is a mouse derived, affinity purified IgG1 monoclonal antibody that binds to fusion proteins containing a FLAG peptide sequence. The M2 antibody will recognize a FLAG peptide sequence at the N-terminus, Met-N-terminus, C-terminus, or internal sites of a fusion protein. Binding of the M2 antibody is not calcium dependent. Monoclonal ANTI-FLAG M2 is useful for detection, identification, and capture of fusion proteins containing a FLAG peptide sequence by common immunological procedures, such as Western blotting, immunofluorescence, and immunoprecipitation. Antigenic binding site: N-Asp-Tyr-Lys-Asp-Asp-Asp-Asp-Lys-C. Specificity: The monoclonal antibody detects only the target protein band(s) on a Western blot from an E. coli, plant or mammalian crude cell lysate. Sensitivity: The monoclonal antibody detects as little as 2 ng of target protein by dot blot. The Western blot is tested down to 10 ng, but may detect lower using the procedure detailed below.

c) Mouse monoclonal antibody clone V5-10 anti-V5 from Sigma cat. #V8012; Monoclonal Anti-V5 (mouse IgG1 isotype) is derived from the V5-10 hybridoma produced by the fusion of mouse myeloma cells and splenocytes from a BALB/c mouse immunized with a synthetic peptide correspond- ing to amino acid residues GKPIPNPLLGLDST (95-108) of the P/V proteins of the Paramyxovirus SV5, conjugated to KLH. The isotype is determined using Mouse Monoclonal Antibody Isotyping Reagents, Catalog No.ISO2. Monoclonal Anti-V5 reacts specifically with V5-tagged recombinant fusion proteins expressed in transfected mammalian cells or produced by in vitro translation. The antibody may be used for ELISA, immunoblotting and immunocytochemistry (methanol-acetone fixation). Product Profile: Immunoblotting: a working concentration of 0.5-1 µg/ml is determined using whole extract of 293T (human embryonal kidney) cells transfected with pcDNA 3.1/V5- His/LacZ plasmid.

d) Mouse monoclonal antibody clone 0.T.81 anti-LacI from abcom cat. #33832; Tested applications: Suitable for: WB, ICC/IF, IHC-FoFr; Species reactivity: Reacts with: Escherichia coli; Positive control: Lac1 transfected Cos-1 cells. Application notes: WB: Use at a concentration of 0.5 - 2 µg/ml. Detects a band of approximately 38 kDa.

e) Mouse monoclonal antibody clone 9E10 anti-cMyc from Sigma cat. #M4439: Monoclonal Anti-c-Myc recognizes an epitope located within the sequence EQKLISEEDL (residues 410-419) of the product of the human oncogene c-myc, known as the c-Myc tag.2 The antibody recognizes the c-Myc tag sequence when it is expressed at either the amino or the carboxyl terminus of the fusion protein. The antibody reacts specifically with c-Myc tagged fusion proteins in immunoblotting, immunoprecipitation and immunofluorescence applications. Reaction of the antibody in immunoblotting is inhibited by the c-Myc peptide (Product No. M 2435). A minimum working dilution of 1:5000 is determined by immunoblotting of an E. Coli extract expressing a recombinant c-Myc-tagged fusion protein. The tagged protein was detected using a chemiluminescent substrate.

f) Rabbit polyclonal antibody anti-TetR from Sigma cat. #T0951; Anti-Tet Repressor (TETR) is produced in rabbit using as immunogen a peptide corresponding to the 22 kDa Tet repressor protein (amino acids 31-49). The antibody is affinity-purified using the immunizing peptide immobilized on agarose. Anti-Tet Repressor (TETR) reacts with amino acid residues 31-49 (AQKLGVEQPTLYWHVKNKR) of the Tet repressor protein. The antibody may be used in indirect ELISA and immunoblotting (~22 kDa, calculated). Product Profile Immunoblotting: a working dilution of ~1:1,000 is recommended.

g) Mouse monoclonal antibody clone 7.23 anti-Cre recombinase from BioLegend cat. #900901: This antibody is effective in immunoblotting (WB). The optimal working dilution should be determined for each specific assay condition, recomended WB: 1:500.

h) Mouse monoclonal antibody clone 7A9 anti-Cas9 from BioLegend cat. #844301 is IP, WB - Validated; For western blotting, the suggested use of this reagent is 0.5 - 1.0 µg per ml. It is recommended that the reagent be titrated for optimal performance for each application.
