# Supplementary material for: A genetic toolkit and gene switches to limit Mycoplasma growth for biosafety applications
Source: Nat Commun. 2022 Apr 7;13:1910. doi: 10.1038/s41467-022-29574-0 (PMC8991246; doi:10.1038/s41467-022-29574-0)
Supplement: Supplementary file 4 — Description of Additional Supplementary Files [file 41467_2022_29574_MOESM4_ESM.pdf]

Title: Supplementary Data 1
Description: Tables of key results and materials. (1A) Table of results of the *in silico* prediction of promoters for *M. pneumoniae*; (1B) Table of DNA sequences of all parts used or design for *M. pneumoniae* in this work; (1C) Table of DNA sequences of tools design for *M. pneumoniae* in this work; (1D) Table of DNA sequences of Kill switches and circuits engineered for *M. pneumoniae* in this work; (1E) Table - List of relevant primers used in this work; (1F) Table - List of MTn vectors used in this work; (1G) Table - List of Strains used in this work.

Title: Supplementary Data 2
Description: Variant calling sequence analyses to explore potential mutations in the population that could be generating escapees for the kill-switch mechanism.

Title: Supplementary Data 3
Description: Full list of sequence variants found in this study (related to main Table 1). Columns correspond to genome loci (base pair position in the C5 genome; POS); Estimate of the probability of a polymorphism at the loci described by the record (QUAL). This value is presented in phred scale and takes into consideration both mapping and genotype quality for a specific variant; total number of reads obtained for the loci (TOT); Total number of reads matching the reference (REFN); number of reads presenting a variant (ALTN); Frequency of the variant normalized by the total reads found mapping that specific loci (FRAC); Reference sequence in the genome (REF); variant sequence (ALT); Degree of impact of the variant (IMPACT), which is classified as "LOW" (synonymous mutations), "MODERATE" (missense), "HIGH" (non-synonymous mutations, start or stop loss), or MODIFIER (variant found in an intergenic region); gene affected (AFF, in case the mutation is found intergenic, it is reported the closest downstream gene); Mutation or variant (MUT; an asterisk indicating stop codon); potential effect of the variant as given by snpeff (EFF); and, in last column (INFO), short explanation of the expected effect of the variant in the mechanism of the kill-switch based on the literature.

Title: Supplementary Data 4
Description: Table of growth upon gene disruption of MPN genes. Growth-after-disruption of the single genes has been compared to the wild-type growth of M. pneumoniae strain M129 in iEG158_mpn and percentages of decrease in growth have been computed.

Title: Supplementary Software
Description: ksanalysis software: 1. Variant calling analysis for the killswitch circuit; 2. Selection of variants; 3. Evaluation of effect of the selected mutations; 4. Mutation rate study.
