# Supplementary material for: A genetic toolkit and gene switches to limit Mycoplasma growth for biosafety applications
Source: Nat Commun. 2022 Apr 7;13:1910. doi: 10.1038/s41467-022-29574-0 (PMC8991246; doi:10.1038/s41467-022-29574-0)
Supplement: Supplementary file 5 — Supplementary Software [file 41467_2022_29574_MOESM5_ESM.zip › Supplementary Software.pdf]

ksanalysis

June 15, 2021

1 Variant calling analysis Killswitch circuit

Defining variants (SNPs, insertions and deletions) for a set of HiSeq samples exploring the variability in populations of *M. pneumoniae* transformed with a killswitch cassette (C5 samples) or two (C20 samples); sequencing performed at different selection passages (2, 3, 15; 2 replicates in each condition and cassette) and a pair of replicates at passage 3 were the cassette was induced by IPTG.

Authors: Samuel Miravet-Verde and Alicia Broto

Last update: 14/06/2021

[662]:
```
%load_ext autoreload
%autoreload 2
%matplotlib widget

import sys, os
import glob
import pandas as pd
import numpy as np
import seaborn as sns
import matplotlib.pyplot as plt
import scripts
from collections import Counter
from Bio import SeqIO
from Bio.SeqFeature import SeqFeature, FeatureLocation

sns.set_style("whitegrid", {'axes.grid' : False})
```

```
The autoreload extension is already loaded. To reload it, use:
  %reload_ext autoreload
```

[663]:
```
# General info about the genome references and the location of the cassettes in␣
↪the genomes
seq_record5 = SeqIO.read('./data/C5_refSeq.gb', "genbank")
seq_record20 = SeqIO.read('./data/C20_refSeq.gb', "genbank")
genome_C5 = str(seq_record5.seq)
genome_C20 = str(seq_record5.seq)
```

```python
cass_par = [565510, 572124] # Present in C5 and C20
cass_3b = [780850, 787445]  # Only in C20

# Annotation information to work faster with dataframes
ncbi = pd.read_csv('./data/mpn_annotation.csv', sep='\t', header=None) # Genome␣
↪annotation
ncbi.columns = ['gene', 'start', 'end', 'strand']
ncbi.set_index('gene', inplace=True)
gold = pd.read_csv('./data/goldsets.csv', sep='\t')  # Gold set

posN = []
posE = []
for gene, cat in zip(gold['gene'], gold['class']):
    if cat=='E':
        posE += range(ncbi.loc[gene][0], ncbi.loc[gene][1]+1)
    else:
        posN += range(ncbi.loc[gene][0], ncbi.loc[gene][1]+1)

# Define the different set of positions in the genome were we will perform the␣
↪analysis
posN = set(posN)
posE = set(posE)
cas1 = set(range(cass_par[0], cass_par[1]+1))
cas2 = set(range(cass_3b[0], cass_3b[1]+1))
cas12 = cas1.union(cas2)
geno = set(range(1, 816395))

ingene = []
for st, en in zip(ncbi['start'], ncbi['end']):
    ingene+=list(range(st, en))
ingene = set(ingene)
outgene = geno.difference(ingene)
```

1.1 1. Map variations

We will be using Snippy for the first steps related to sequence mapping. To run it in parallel for all the samples we create a input file:

[664]:
```python
### 1. Make a multifile to map with snippy-multi.
files = glob.glob('../data/C[5|20]*/*_R1_*') # The gre expression refers to the␣
↪raw sequencing files located in data (this has to point to the directory␣
↪where fastq files are stored)

fo1 = open('./tmp_files/snippy_input1.txt', 'w')
fo2 = open('./tmp_files/snippy_input2.txt', 'w')
```

```
for f in files:
    ide = f.split('/')[2:]
    if 'C5' in f:
        ide = ide[0].replace('C5', '')+'_'+'_'.join(ide[1].split('_')[:-3])
        fo1.write('{}\t{}\t{}\n'.format(ide, f, f.replace('_R1_', '_R2_')))
    else:
        ide = ide[0].replace('C20', '')+'_'+'_'.join(ide[1].split('_')[:-3])
        fo2.write('{}\t{}\t{}\n'.format(ide, f, f.replace('_R1_', '_R2_')))
fo1.close()
fo2.close()
files
```

[664]:
```
['../data/C20p3/B_S1_L001_R1_001.fastq.gz',
 '../data/C20p3/B_S2_L001_R1_001.fastq.gz',
 '../data/C5p3/D_S2_L001_R1_001.fastq.gz',
 '../data/C5p3/D_S3_L001_R1_001.fastq.gz',
 '../data/C5p3IPTG/A_S1_L001_R1_001.fastq.gz',
 '../data/C5p3IPTG/A_S1b_L001_R1_001.fastq.gz',
 '../data/C20p15/7_S3_L001_R1_001.fastq.gz',
 '../data/C20p15/7_S5_L001_R1_001.fastq.gz',
 '../data/C5p15/F_S5_L001_R1_001.fastq.gz',
 '../data/C5p15/F_S1_L001_R1_001.fastq.gz',
 '../data/C5p2/2_S2_L001_R1_001.fastq.gz',
 '../data/C5p2/2_S3_L001_R1_001.fastq.gz',
 '../data/C5p2/2_S1_L001_R1_001.fastq.gz',
 '../data/C20p2/6_S2_L001_R1_001.fastq.gz',
 '../data/C20p2/6_S4_L001_R1_001.fastq.gz']
```

Now run the command in the snippy environment:

```
conda activate snippy
snippy-multi ./data/snippy_input.txt --ref ./data/C5_refSeq.gb --cpus 4 --report >  ./snippy_ru
sh ./snippy_run.sh
```

Run freebayes in a permissive manner. This command will create a directory with the name of the sample were all the files will be stored. This is expected to be run in the terminal copy-pasting the printed command. It can also be run changing the print to `os.system` to execute it for each file:

[5]:
```
# Listed samples in order
order1 = ['p2_2_S1','p2_2_S2','p2_2_S3','p3_D_S2',␣
↪'p3_D_S3','p15_F_S1','p15_F_S5', 'p3IPTG_A_S1', 'p3IPTG_A_S1b']
order2 = ['p2_6_S2','p2_6_S4','p3_B_S1','p3_B_S2','p15_7_S3','p15_7_S5']
```

[6]:
```
# freebayes-parallel reference/ref.txt 4 -p 2 -P 0 -C 2 -F 0.05 --min-coverage␣
↪10 --min-repeat-entropy 1.0 -q 13 -m 60 --strict-vcf   -f reference/ref.fa␣
↪snps.bam > snps.raw.vcf
for fil in glob.glob('../p*/*.bam'):
    # again this has to point to directories were snipy output is stored
```

```
    ide = fil.split('/')[1]
    #print('freebayes-parallel {}/reference/ref.txt 4 -p 1 -P 0 -C 1 -F 0.01 -q␣
↪13 -m 60 --strict-vcf -f {}/reference/ref.fa {} > filter10/{}.raw.vcf'.
↪format(ide, ide, fil, ide))
    #print('freebayes-parallel {}/reference/ref.txt 4 -p 1 -P 0 -C 2 -F 0.05 -q␣
↪13 -m 60 --strict-vcf -f {}/reference/ref.fa {} > filter50/{}.raw.vcf'.
↪format(ide, ide, fil, ide))
    if ide in order1:
        print('freebayes-parallel {}/reference/ref.txt 4 -p 1 -P 0 -C 1 -F 0.
↪001 -q 13 -m 60 --strict-vcf -f {}/reference/ref.fa {} > filter1/{}.raw.vcf'.
↪format(ide, ide, fil, ide))
    else:
        print('freebayes-parallel {}/reference/ref.txt 4 -p 1 -P 0 -C 1 -F 0.
↪001 -q 13 -m 60 --strict-vcf -f {}/reference/ref.fa {} > filter2/{}.raw.vcf'.
↪format(ide, ide, fil, ide))
```

```
freebayes-parallel p3_D_S2/reference/ref.txt 4 -p 1 -P 0 -C 1 -F 0.001 -q 13 -m
60 --strict-vcf -f p3_D_S2/reference/ref.fa ../p3_D_S2/snps.bam >
filter1/p3_D_S2.raw.vcf
freebayes-parallel p2_6_S2/reference/ref.txt 4 -p 1 -P 0 -C 1 -F 0.001 -q 13 -m
60 --strict-vcf -f p2_6_S2/reference/ref.fa ../p2_6_S2/snps.bam >
filter2/p2_6_S2.raw.vcf
freebayes-parallel p2_2_S1/reference/ref.txt 4 -p 1 -P 0 -C 1 -F 0.001 -q 13 -m
60 --strict-vcf -f p2_2_S1/reference/ref.fa ../p2_2_S1/snps.bam >
filter1/p2_2_S1.raw.vcf
freebayes-parallel p15_7_S5/reference/ref.txt 4 -p 1 -P 0 -C 1 -F 0.001 -q 13 -m
60 --strict-vcf -f p15_7_S5/reference/ref.fa ../p15_7_S5/snps.bam >
filter2/p15_7_S5.raw.vcf
freebayes-parallel p3IPTG_A_S1/reference/ref.txt 4 -p 1 -P 0 -C 1 -F 0.001 -q 13
-m 60 --strict-vcf -f p3IPTG_A_S1/reference/ref.fa ../p3IPTG_A_S1/snps.bam >
filter1/p3IPTG_A_S1.raw.vcf
freebayes-parallel p3_D_S3/reference/ref.txt 4 -p 1 -P 0 -C 1 -F 0.001 -q 13 -m
60 --strict-vcf -f p3_D_S3/reference/ref.fa ../p3_D_S3/snps.bam >
filter1/p3_D_S3.raw.vcf
freebayes-parallel p3_B_S1/reference/ref.txt 4 -p 1 -P 0 -C 1 -F 0.001 -q 13 -m
60 --strict-vcf -f p3_B_S1/reference/ref.fa ../p3_B_S1/snps.bam >
filter2/p3_B_S1.raw.vcf
freebayes-parallel p15_7_S3/reference/ref.txt 4 -p 1 -P 0 -C 1 -F 0.001 -q 13 -m
60 --strict-vcf -f p15_7_S3/reference/ref.fa ../p15_7_S3/snps.bam >
filter2/p15_7_S3.raw.vcf
freebayes-parallel p2_2_S3/reference/ref.txt 4 -p 1 -P 0 -C 1 -F 0.001 -q 13 -m
60 --strict-vcf -f p2_2_S3/reference/ref.fa ../p2_2_S3/snps.bam >
filter1/p2_2_S3.raw.vcf
freebayes-parallel p15_F_S1/reference/ref.txt 4 -p 1 -P 0 -C 1 -F 0.001 -q 13 -m
60 --strict-vcf -f p15_F_S1/reference/ref.fa ../p15_F_S1/snps.bam >
filter1/p15_F_S1.raw.vcf
```

```
freebayes-parallel p2_6_S4/reference/ref.txt 4 -p 1 -P 0 -C 1 -F 0.001 -q 13 -m
60 --strict-vcf -f p2_6_S4/reference/ref.fa ../p2_6_S4/snps.bam >
filter2/p2_6_S4.raw.vcf
freebayes-parallel p2_2_S2/reference/ref.txt 4 -p 1 -P 0 -C 1 -F 0.001 -q 13 -m
60 --strict-vcf -f p2_2_S2/reference/ref.fa ../p2_2_S2/snps.bam >
filter1/p2_2_S2.raw.vcf
freebayes-parallel p15_F_S5/reference/ref.txt 4 -p 1 -P 0 -C 1 -F 0.001 -q 13 -m
60 --strict-vcf -f p15_F_S5/reference/ref.fa ../p15_F_S5/snps.bam >
filter1/p15_F_S5.raw.vcf
freebayes-parallel p3_B_S2/reference/ref.txt 4 -p 1 -P 0 -C 1 -F 0.001 -q 13 -m
60 --strict-vcf -f p3_B_S2/reference/ref.fa ../p3_B_S2/snps.bam >
filter2/p3_B_S2.raw.vcf
freebayes-parallel p3IPTG_A_S1b/reference/ref.txt 4 -p 1 -P 0 -C 1 -F 0.001 -q
13 -m 60 --strict-vcf -f p3IPTG_A_S1b/reference/ref.fa ../p3IPTG_A_S1b/snps.bam
> filter1/p3IPTG_A_S1b.raw.vcf
```

To get the total number of mapped reads:

```
for fil in glob.glob('./p*/*.bam'):
    ide = fil.split('/')[1]
    # get the total number of reads of a BAM file (may include unmapped and duplicated multi-a
    print('samtools view -c {} >> total_readcount.txt'.format(fil))
    print('samtools view -c {} -F 260 >> total_readcount_mapped.txt'.format(fil))
```

Finally we annotate the effect of the variants using SnpEff. This requires to add the custom annotation to the sources, specifying that the translation code is set to 4 for *M. pneumoniae*.

1. Download SnpEff and edited the config (in software_crg/SnpEff) to include the C5 and C20 refseq references
2. Build the custom db:

```
java -jar snpEff.jar build -genbank -v C5ali
java -jar snpEff.jar build -genbank -v C20ali
```

3. Annotate vcf files in bash (in directory of the software)

```
for fil in `ls ./filter1/p*.vcf`; do java -Xmx8g -jar snpEff.jar C5ali $fil > $fil.ann; do
for fil in `ls ./filter2/p*.vcf`; do java -Xmx8g -jar snpEff.jar C20ali $fil > $fil.ann; d
```

1.2 2. Data loading

We will parse the variations to keep the columns of interest to perform the exploratory analysis. Dataframes include the following information (by column header): - SAMPLE: sample identifier includes information about the passage (e.g. p3IPTG is the passage 3 induced) - PASS: passage (2,3 or 15) - POS: genome loci (base pair position) where the variant is found - QUAL: estimate of the probability that there is a polymorphism at the loci described by the record - TOT: total number of reads covering a loci - REFN: total number of reads covering a loci matching the reference - ALTN: number of reads presenting a variant - REF: reference sequence - ALT: alternative sequence - EFF: potential effect of the variant as given by snpeff - IMPACT: potential impact of the variant, can be LOW (synonimous mutations), MODERATE (missense), HIGH (non-synonimous mutations,

start lost, stop lost), or MODIFIER (when it occurs in an intergenic region) - AFF: gene affected - MUT: mutation (in nucleotide if not coding in amino acid if coding) - ANN_TYPE: annotation of the loci, can be intergenic, gene, or cassette (covering the principal elements in the cassette)

[7]:
```
# This will concat all the annotated vcf files keeping the fields of interest
data1 = scripts.parse_variations('./filter1/*.raw.vcf.ann', order1, genome=5,␣
↪fname='./results/data1.pickle')
data2 = scripts.parse_variations('./filter2/*.raw.vcf.ann', order2, genome=20,␣
↪fname='./results/data2.pickle')
```

```
Loading data for genome 5
Loading data for genome 20
```

We can now explore the variants, for example to extract those present in the cassette with high impact (non-synonimous) in samples induced (assigned as passage 18 despite they are passage 3), could be retrieved as:

[8]:
```
# Example:
## Notice the information is grouped by position, we will deal with this in␣
↪section 3.
data1[(data1['ANN_TYPE']=='cassette') & (data1['IMPACT']=='HIGH') &␣
↪(data1['PASS']==18)].sort_values('FRAC')
```

[8]:
```
                SAMPLE  PASS     POS          QUAL  TOT  REFN  ALTN       FRAC  \
443045     p3IPTG_A_S1    18  566372  2.184800e-15  677   676     1   0.147929
443082     p3IPTG_A_S1    18  566622  0.000000e+00  647   646     1   0.154799
443002     p3IPTG_A_S1    18  565989  2.863080e-15  582   581     1   0.172117
443294     p3IPTG_A_S1    18  568518  7.700020e-15  577   576     1   0.173611
443402     p3IPTG_A_S1    18  569451  6.132760e-15  572   571     1   0.175131
…                  …   …     …             …   …     …    …      …
443663     p3IPTG_A_S1    18  571681  0.000000e+00  481   469    12   2.558635
495218    p3IPTG_A_S1b    18  571681  7.895900e-15  191   182     9   4.945055
495059    p3IPTG_A_S1b    18  569127  8.076710e-15  235   210    25  11.904762
443366     p3IPTG_A_S1    18  569125  0.000000e+00  534   463    71  15.334773
443009     p3IPTG_A_S1    18  566053  1.947940e-13  559   446   113  25.336323

                   REF            ALT                                  EFF  \
443045             CTA            TTT                          stop_gained
443082               A              C                          stop_gained
443002         TCCCTCG         TCCTCG                   frameshift_variant
443294               G              A                          stop_gained
443402               C              A                          stop_gained
…                  …              …                                  …
443663     ATTTTTTTGATA     ATTTTTTTGATA                 frameshift_variant
495218  ATTTTTTTTGATACT  ATTTCTTTAGAAACA                    stop_gained
495059          CAAACT          CAACT                   frameshift_variant
443366         ATCAAACT        GTCAACC  frameshift_variant&missense_variant
443009          CGCAAA         TGCTAT                          stop_gained
```

	IMPACT	AFF	MUT	ANN_TYPE
443045	HIGH	LacI4	p.LeuGly102*	cassette
443082	HIGH	LacI4	p.Tyr19*	cassette
443002	HIGH	LacI4	p.Gly230fs	cassette
443294	HIGH	cas9B	p.Gln1101*	cassette
443402	HIGH	cas9B	p.Glu790*	cassette
…	…	…	…	…
443663	HIGH	cas9B	p.Asn46fs	cassette
495218	HIGH	cas9B	p.SerIleLysLys42*	cassette
495059	HIGH	cas9B	p.Phe897fs	cassette
443366	HIGH	cas9B	p.Lys896fs	cassette
443009	HIGH	LacI4	p.LeuArg208*	cassette

[101 rows x 15 columns]

[9]:
```python
# For C20 we have snpcalls2 in the same format
data2[(data2['ANN_TYPE']=='cassette') & (data2['IMPACT']=='HIGH') &␣
↪(data2['PASS']==3)].sort_values('FRAC')
```

[9]:

	SAMPLE	PASS	POS	QUAL	TOT	REFN	ALTN	FRAC	\
225393	p3_B_S1	3	786189	0.000000e+00	280	279	1	0.358423	
225340	p3_B_S1	3	785573	2.991160e-15	253	252	1	0.396825	
225346	p3_B_S1	3	785643	0.000000e+00	252	251	1	0.398406	
264083	p3_B_S2	3	566670	0.000000e+00	226	225	1	0.444444	
225402	p3_B_S1	3	787394	0.000000e+00	150	149	1	0.671141	
277049	p3_B_S2	3	785709	1.702880e-15	258	256	2	0.781250	
277050	p3_B_S2	3	785714	1.642610e-15	255	253	2	0.790514	
209632	p3_B_S1	3	566676	1.994980e-14	226	224	2	0.892857	
209630	p3_B_S1	3	566634	2.558530e-14	112	111	1	0.900901	
225397	p3_B_S1	3	786318	0.000000e+00	98	97	1	1.030928	
225356	p3_B_S1	3	785712	0.000000e+00	264	261	3	1.149425	

	REF	ALT	EFF	IMPACT	AFF	\
225393	TTAC	CTAT	stop_gained	HIGH	cas2 cat	
225340	GAAAAAAATCAC	GAAAAAAAATCAC	frameshift_variant	HIGH	cas2 cat	
225346	C	T	stop_gained	HIGH	cas2 cat	
264083	CTTTGC	CTTGC	frameshift_variant	HIGH	cas1 LacI4	
225402	AGCG	TGCA	stop_lost	HIGH	cas2 lacI4	
277049	GTA	GA	frameshift_variant	HIGH	cas2 cat	
277050	GAAAAATAAGC	GAAAATAAGC	frameshift_variant	HIGH	cas2 cat	
209632	CATA	TATT	start_lost	HIGH	cas1 LacI4	
209630	AGGCT	AGGGCT	frameshift_variant	HIGH	cas1 LacI4	
225397	GCCTG	GCCCTG	frameshift_variant	HIGH	cas2 lacI4	
225356	AAGAAAA	TAGTAAT	stop_gained	HIGH	cas2 cat	

MUT ANN_TYPE

```
225393                 p.Gln209*  cassette
225340                  p.Ile5fs  cassette
225346                  p.Gln26*  cassette
264083                  p.Lys3fs  cassette
225402  p.TerArg373LeuGlnext*?  cassette
277049                 p.Val48fs  cassette
277050                 p.Asn51fs  cassette
209632                   p.Met1?  cassette
209630                 p.Val16fs  cassette
225397                 p.Val16fs  cassette
225356            p.LysLysAsn49*  cassette
```

[644]:
```python
# Save supplementary 1
sup1 = './results/suptableS1.xlsx'
if not os.path.isfile(sup1):
    writer = pd.ExcelWriter(sup1, engine='xlsxwriter')
    for c, data in zip(['C5_', 'C20_'], [data1, data2]):
        for sample in set(data['SAMPLE']):
            subdata = data[data['SAMPLE']==sample].copy()
            subdata.to_excel(writer, sheet_name=c+sample)
    writer.save()
```

1.3 3. Rate and fraction of mutations in cassette compared to other distributions (supplementary figure 1)

Explore the frequency at which we found a variant within the cassette compared to other types of annotations and also their representation within the population by means of the fraction values.

[665]:
```python
def plot_percentage(df, genome=5):
    """ Plot to show the percentage of variants mapping to each type of␣
↪annotation """
    rs = {}
    c = 0
    lens = {}
    lens['gene'] = len(ingene)
    lens['intergenic'] = len(outgene)
    lens['essential'] = len(posE)
    lens['non-essential'] = len(posN)
    if genome==5:
        lens['chromosome'] = len(genome_C5)
        lens['cassette'] = len(cas1)
    else:
        lens['chromosome'] = len(genome_C20)
        lens['cassette'] = len(cas12)
    # Extract positions in passage 2
    for pas in set(df['PASS']):
```

```
        for sample in set(df[df['PASS']==pas]['SAMPLE']):
            for impact in set(df['IMPACT']):
                rs[c] = [pas,
                         100*len(set(df[(df['PASS']==pas) &␣
 ↪(df['IMPACT']==impact)  & (df['SAMPLE']==sample)]['POS']))/
 ↪lens['chromosome'],
                         sample, 'TOTAL', impact]
                c+=1
            rs[c] = [pas, 100*len(set(df[(df['PASS']==pas)  &␣
 ↪(df['SAMPLE']==sample)]['POS']))/lens['chromosome'],sample, 'chromosome',␣
 ↪'TOTAL']
            c+=1
            for ann in set(df['ANN_TYPE']):
                if ann in lens:
                    rs[c] = [pas,
                             100*len(set(df[(df['PASS']==pas) &␣
 ↪(df['ANN_TYPE']==ann)  & (df['SAMPLE']==sample)]['POS']))/lens[ann],
                             sample, ann, 'TOTAL']
                    c+=1
                    for impact in set(df['IMPACT']):
                        rs[c] = [pas,
                                 100*len(set(df[(df['PASS']==pas) &␣
 ↪(df['ANN_TYPE']==ann) & (df['IMPACT']==impact)  &␣
 ↪(df['SAMPLE']==sample)]['POS']))/lens[ann],
                                 sample, ann, impact]
                        c+=1
    rs = pd.DataFrame.from_dict(rs, orient='index')
    rs.columns = ['PASS', 'PERC', 'sample', 'annotation', 'IMPACT']
    rs['HUE'] = rs['annotation']+rs['IMPACT']
    return rs.sort_values('annotation')

subdf1 = data1[(data1['FRAC']<100) & (data1['ALTN']>=2)].
 ↪sort_values(['ANN_TYPE', 'PASS']).copy()
subdf2 = data2[(data2['FRAC']<100) & (data2['ALTN']>=2)].
 ↪sort_values(['ANN_TYPE', 'PASS']).copy()

plot1 = plot_percentage(subdf1)
plot2 = plot_percentage(subdf2)

subdf1['log2Frac'] = np.log2(subdf1['FRAC'])
subdf1['Condition'] = ['{}'.format(i) if i!=18 else '3IPTG' for i in␣
 ↪subdf1['PASS']]
subdf1['annotation'] = subdf1['ANN_TYPE']

subdf2['log2Frac'] = np.log2(subdf2['FRAC'])
```

```
subdf2['Condition'] = ['{}'.format(i) if i!=18 else '3IPTG' for i in␣
↪subdf2['PASS']]
subdf2['annotation'] = subdf2['ANN_TYPE']

plt.close('all')
plt.figure(figsize=(10, 10))
plt.subplot(2,3,1)
sns.countplot(x='Condition', hue='annotation', data=subdf1[(subdf1['ANN_TYPE'].
↪isin(['cassette', 'gene', 'intergenic']))])

plt.subplot(2,3,2)
sns.barplot(x='PASS', y='PERC', hue='annotation',␣
↪data=plot1[(plot1['IMPACT']=='TOTAL') & (plot1['annotation'].
↪isin(['cassette', 'gene', 'intergenic']))])
plt.xticks([0,1,2,3], ['2', '3', '15', '3IPTG'])
plt.xlabel('Condition')
plt.ylabel('Percentage')
plt.title('C5 samples')

plt.subplot(2,3,3)
sns.boxplot(y='log2Frac', x='Condition', hue='annotation',␣
↪data=subdf1[(subdf1['log2Frac']>0) & (subdf1['ANN_TYPE'].isin(['cassette',␣
↪'gene', 'intergenic']))])
plt.xlabel('Condition')
plt.ylabel('log2(Fraction)')

#plt.ylim(0,3.2)
plt.subplot(2,3,4)
sns.countplot(x='PASS', hue='annotation', data=subdf2[(subdf2['ANN_TYPE'].
↪isin(['cassette', 'gene', 'intergenic']))])
plt.xlabel('Condition')

plt.subplot(2,3,5)
sns.barplot(x='PASS', y='PERC', hue='annotation',␣
↪data=plot2[(plot2['IMPACT']=='TOTAL') & (plot2['annotation'].
↪isin(['cassette', 'gene', 'intergenic']))])
plt.xlabel('Condition')
plt.ylabel('Percentage')
plt.title('C20 samples')
#plt.ylim(0,3.2)

plt.subplot(2,3,6)
sns.boxplot(y='log2Frac', x='PASS', hue='annotation',␣
↪data=subdf2[(subdf2['log2Frac']>0) & (subdf2['ANN_TYPE'].isin(['cassette',␣
↪'gene', 'intergenic']))])
plt.xlabel('Condition')
```

```
plt.ylabel('log2(Fraction)')

plt.tight_layout()
plt.savefig('./results/supfigS1.svg')
```

```
Canvas(toolbar=Toolbar(toolitems=[('Home', 'Reset original view', 'home', 'home'), ('Back', 'Ba
```

Let's calculate the statistics for this comparisons:

[666]:
```
percentage_pvalues = {}
fraction_pvalues = {}

perc1 = plot1[(plot1['IMPACT']=='TOTAL') & (plot1['annotation'].
 ↪isin(['cassette', 'gene', 'intergenic']))].copy()
perc2 = plot2[(plot2['IMPACT']=='TOTAL') & (plot2['annotation'].
 ↪isin(['cassette', 'gene', 'intergenic']))].copy()

frac1 = subdf1[(subdf1['log2Frac']>0) & (subdf1['ANN_TYPE'].isin(['cassette',␣
 ↪'gene', 'intergenic']))].copy()
frac2 = subdf2[(subdf2['log2Frac']>0) & (subdf2['ANN_TYPE'].isin(['cassette',␣
 ↪'gene', 'intergenic']))].copy()

c = 0
for pas in set(perc1['PASS']):
    x = perc1[(perc1['PASS']==pas) & (perc1['annotation']=='cassette')]['PERC']
    for annot in ['gene', 'intergenic']:
        y = perc1[(perc1['PASS']==pas) & (perc1['annotation']==annot)]['PERC']
        percentage_pvalues[c] = ['C5', pas, annot, round(mannwhitneyu(x, y)[1]/
 ↪2, 4)]
        c+=1
for pas in set(perc2['PASS']):
    x = perc2[(perc2['PASS']==pas) & (perc2['annotation']=='cassette')]['PERC']
    for annot in ['gene', 'intergenic']:
        y = perc2[(perc2['PASS']==pas) & (perc2['annotation']==annot)]['PERC']
        percentage_pvalues[c] = ['C20', pas, annot,round(mannwhitneyu(x, y)[1]/
 ↪2, 4)]
        c+=1

c = 0
for pas in set(frac1['PASS']):
    x = frac1[(frac1['PASS']==pas) &␣
 ↪(frac1['annotation']=='cassette')]['log2Frac']
    for annot in ['gene', 'intergenic']:
        y = frac1[(frac1['PASS']==pas) &␣
 ↪(frac1['annotation']==annot)]['log2Frac']
```

```
        fraction_pvalues[c] = ['C5', pas, annot, round(mannwhitneyu(x, y)[1]/2,␣
↪4)]
        c+=1
for pas in set(frac2['PASS']):
    x = frac2[(frac2['PASS']==pas) &␣
↪(frac2['annotation']=='cassette')]['log2Frac']
    for annot in ['gene', 'intergenic']:
        y = frac2[(frac2['PASS']==pas) &␣
↪(frac2['annotation']==annot)]['log2Frac']
        fraction_pvalues[c] = ['C20', pas, annot,round(mannwhitneyu(x, y)[1]/2,␣
↪4)]
        c+=1
```

[667]:
```
print('Average rate of mutations per base in the cassette', np.
↪mean(perc1[(perc1['annotation']=='cassette')]['PERC']))
print('Average rate of mutations per base in the intergenic', np.
↪mean(perc1[(perc1['annotation']=='intergenic')]['PERC']))
print('Average rate of mutations per base in the gene', np.
↪mean(perc1[(perc1['annotation']=='gene')]['PERC']))

print('Average rate of mutations per base in the cassette', np.
↪mean(perc2[(perc2['annotation']=='cassette')]['PERC']))
print('Average rate of mutations per base in the intergenic', np.
↪mean(perc2[(perc2['annotation']=='intergenic')]['PERC']))
print('Average rate of mutations per base in the gene', np.
↪mean(perc2[(perc2['annotation']=='gene')]['PERC']))
```

```
Average rate of mutations per base in the cassette 2.825228856974889
Average rate of mutations per base in the intergenic 1.8428205685101458
Average rate of mutations per base in the gene 1.4096396774343913
Average rate of mutations per base in the cassette 0.9750566893424035
Average rate of mutations per base in the intergenic 2.42446081044616
Average rate of mutations per base in the gene 1.8454062368899453
```

[668]:
```
percentage_pvalues
```

[668]:
```
{0: ['C5', 18, 'gene', 0.0613],
 1: ['C5', 18, 'intergenic', 0.1746],
 2: ['C5', 2, 'gene', 0.0476],
 3: ['C5', 2, 'intergenic', 0.0476],
 4: ['C5', 3, 'gene', 0.1746],
 5: ['C5', 3, 'intergenic', 0.1746],
 6: ['C5', 15, 'gene', 0.0613],
 7: ['C5', 15, 'intergenic', 0.0613],
 8: ['C20', 2, 'gene', 0.0613],
 9: ['C20', 2, 'intergenic', 0.0613],
```

```
 10: ['C20', 3, 'gene', 0.0613],
 11: ['C20', 3, 'intergenic', 0.0613],
 12: ['C20', 15, 'gene', 0.0613],
 13: ['C20', 15, 'intergenic', 0.0613]}
```

[669]:
```
print('Average rate of mutations per base in the cassette', np.
 ↪percentile(frac1[(frac1['annotation']=='cassette')  &␣
 ↪(frac1['log2Frac']>0)]['FRAC'], 50))
print('Average rate of mutations per base in the intergenic', np.
 ↪percentile(frac1[(frac1['annotation']=='intergenic') & (frac1['log2Frac']>0)␣
 ↪]['FRAC'], 50))
print('Average rate of mutations per base in the gene', np.
 ↪percentile(frac1[(frac1['annotation']=='gene')&␣
 ↪(frac1['log2Frac']>0)]['FRAC'], 50))

print('Average rate of mutations per base in the cassette', np.
 ↪median(frac2[(frac2['annotation']=='cassette')]['FRAC']))
print('Average rate of mutations per base in the intergenic', np.
 ↪median(frac2[(frac2['annotation']=='intergenic')]['FRAC']))
print('Average rate of mutations per base in the gene', np.
 ↪median(frac2[(frac2['annotation']=='gene')]['log2Frac']))
```

```
Average rate of mutations per base in the cassette 1.492537260055542
Average rate of mutations per base in the intergenic 1.5564202070236206
Average rate of mutations per base in the gene 1.470588207244873
Average rate of mutations per base in the cassette 1.5151515
Average rate of mutations per base in the intergenic 1.3513514
Average rate of mutations per base in the gene 0.38646838
```

[670]:
```
fraction_pvalues
```

[670]:
```
{0: ['C5', 18, 'gene', 0.0117],
 1: ['C5', 18, 'intergenic', 0.2241],
 2: ['C5', 2, 'gene', 0.0],
 3: ['C5', 2, 'intergenic', 0.0],
 4: ['C5', 3, 'gene', 0.1343],
 5: ['C5', 3, 'intergenic', 0.0988],
 6: ['C5', 15, 'gene', 0.1662],
 7: ['C5', 15, 'intergenic', 0.0021],
 8: ['C20', 2, 'gene', 0.0002],
 9: ['C20', 2, 'intergenic', 0.0016],
 10: ['C20', 3, 'gene', 0.0],
 11: ['C20', 3, 'intergenic', 0.0],
 12: ['C20', 15, 'gene', 0.0],
 13: ['C20', 15, 'intergenic', 0.002]}
```

1.4 4. Location of the mutations within the cassettes (supplementary figure 2)

[682]:
```
subdf1 = data1[(data1['FRAC']<100) & (data1['ALTN']>=2) & (data1['QUAL']>0.0)].
 ↪sort_values(['ANN_TYPE', 'PASS']).copy()
subdf2 = data2[(data2['FRAC']<100) & (data2['ALTN']>=2) & (data2['QUAL']>0.0)].
 ↪sort_values(['ANN_TYPE', 'PASS']).copy()

plot1 = plot_percentage(subdf1)
plot2 = plot_percentage(subdf2)

subdf1['log2Frac'] = np.log2(subdf1['FRAC'])
subdf1['Condition'] = ['{}'.format(i) if i!=18 else '3IPTG' for i in␣
 ↪subdf1['PASS']]
subdf1['annotation'] = subdf1['ANN_TYPE']

subdf2['log2Frac'] = np.log2(subdf2['FRAC'])
subdf2['Condition'] = ['{}'.format(i) if i!=18 else '3IPTG' for i in␣
 ↪subdf2['PASS']]
subdf2['annotation'] = subdf2['ANN_TYPE']

plt.close('all')
plt.figure(figsize=(15, 10))
plt.subplot(2,2,1)
sns.countplot(x='AFF', hue='Condition', data=subdf1[(subdf1['ANN_TYPE'].
 ↪isin(['cassette']))])
plt.xticks(rotation=45, ha='right')
plt.xlabel('Element')

plt.subplot(2,2,2)
sns.swarmplot(y='FRAC', x='PASS', hue='AFF', data=subdf1[(subdf1['log2Frac']>0)␣
 ↪& (subdf1['ANN_TYPE'].isin(['cassette']))])
plt.xlabel('Passage')
plt.ylabel('Fraction')
plt.title('C5 samples')
plt.legend(bbox_to_anchor=(1.05, 1))


#plt.ylim(0,3.2)
plt.subplot(2,2,3)
sns.countplot(x='AFF', hue='Condition', data=subdf2[(subdf2['ANN_TYPE'].
 ↪isin(['cassette']))])
plt.xticks(rotation=45, ha='right')
plt.xlabel('Element')


plt.subplot(2,2,4)
```

```
sns.swarmplot(y='FRAC', x='PASS', hue='AFF', data=subdf2[(subdf2['log2Frac']>0)␣
↪& (subdf2['ANN_TYPE'].isin(['cassette']))])
plt.xlabel('Passage')
plt.ylabel('Fraction')
plt.title('C20 samples')
plt.legend(bbox_to_anchor=(1.05, 1))

plt.tight_layout()
plt.savefig('./results/supfigS2.svg')
```

```
Canvas(toolbar=Toolbar(toolitems=[('Home', 'Reset original view', 'home', 'home'), ('Back', 'Ba
```

1.5 5. Exploration of the effect of the mutations explaining the escape rate (supplementary figure 3)

Integrating the previous results we will explore the mutations that could mainly explain the rate of cell escaping the killswitch circuit (we use the median value to filter out non-significant positions)

[679]:
```
plt.figure(figsize=(10,10))
plt.subplot(2,1,1)
sns.swarmplot(x='AFF', y='FRAC', hue='IMPACT',data=subdf1[(subdf1['FRAC']>=1.5)␣
↪& (subdf1['ANN_TYPE']=='cassette')])
plt.xticks(rotation=45, ha='right')
plt.xlabel('Element')
plt.ylabel('Fraction')

plt.subplot(2,1,2)
sns.swarmplot(x='AFF', y='FRAC', hue='IMPACT',data=subdf2[(subdf2['FRAC']>=1.5)␣
↪& (subdf2['ANN_TYPE']=='cassette')])
plt.xticks(rotation=45, ha='right')
plt.xlabel('Element')
plt.ylabel('Fraction')

plt.tight_layout()
plt.savefig('./results/supfigS3.svg')
```

```
Canvas(toolbar=Toolbar(toolitems=[('Home', 'Reset original view', 'home', 'home'), ('Back', 'Ba
```

[677]:
```
selected = data1[(data1['PASS']==18) & (data1['ANN_TYPE'].isin(['cassette']))].
↪sort_values(['FRAC'])
selected['TOP'] = [1 if z>=1.5 else 0 for z in selected['FRAC']]
selected.to_excel('./results/suptableS2.xlsx')
selected
```

```
[677]:              SAMPLE  PASS     POS          QUAL  TOT  REFN  ALTN       FRAC  \
        443045   p3IPTG_A_S1    18  566372  2.184800e-15  677   676     1   0.147929
        443032   p3IPTG_A_S1    18  566245  1.137610e-15  670   669     1   0.149477
        443043   p3IPTG_A_S1    18  566351  0.000000e+00  655   654     1   0.152905
        443077   p3IPTG_A_S1    18  566562  0.000000e+00  655   654     1   0.152905
        443082   p3IPTG_A_S1    18  566622  0.000000e+00  647   646     1   0.154799
        …                  …   …       …           …   …     …     …          …
        494861  p3IPTG_A_S1b    18  566416  3.471600e-14  281   255    26  10.196078
        495059  p3IPTG_A_S1b    18  569127  8.076710e-15  235   210    25  11.904762
        443366   p3IPTG_A_S1    18  569125  0.000000e+00  534   463    71  15.334773
        494831  p3IPTG_A_S1b    18  566053  0.000000e+00  271   224    47  20.982143
        443009   p3IPTG_A_S1    18  566053  1.947940e-13  559   446   113  25.336323

                    REF      ALT                                EFF    IMPACT  \
        443045      CTA      TTT                        stop_gained      HIGH
        443032        G        T                   missense_variant  MODERATE
        443043        T        C                   missense_variant  MODERATE
        443077        T        G                 synonymous_variant       LOW
        443082        A        C                        stop_gained      HIGH
        …           …       …                                  …         …
        494861  GGTGCGT  CGTTCTT                   missense_variant  MODERATE
        495059   CAAACT    CAACT                 frameshift_variant      HIGH
        443366  ATCAAACT  GTCAACC  frameshift_variant&missense_variant      HIGH
        494831    CGCAA    CACAT                   missense_variant  MODERATE
        443009   CGCAAA   TGCTAT                        stop_gained      HIGH

                  AFF                    MUT  ANN_TYPE  TOP
        443045  LacI4            p.LeuGly102*  cassette    0
        443032  LacI4             p.Ala145Asp  cassette    0
        443043  LacI4             p.Met110Val  cassette    0
        443077  LacI4              p.Ala39Ala  cassette    0
        443082  LacI4                p.Tyr19*  cassette    0
        …          …                      …       …   …
        494861  LacI4  p.HisAlaPro86GlnGluArg  cassette    1
        495059  cas9B              p.Phe897fs  cassette    1
        443366  cas9B              p.Lys896fs  cassette    1
        494831  LacI4       p.LeuArg208MetCys  cassette    1
        443009  LacI4            p.LeuArg208*  cassette    1

        [1237 rows x 16 columns]
```

```
[678]: selected[selected['POS'].isin([566423, 571681, 566053])]
```

```
[678]:              SAMPLE  PASS     POS          QUAL  TOT  REFN  ALTN       FRAC  \
        443045   p3IPTG_A_S1    18  566372  2.184800e-15  677   676     1   0.147929
        443032   p3IPTG_A_S1    18  566245  1.137610e-15  670   669     1   0.149477
        443043   p3IPTG_A_S1    18  566351  0.000000e+00  655   654     1   0.152905
```

```
443077    p3IPTG_A_S1    18  566562  0.000000e+00  655    654     1   0.152905
443082    p3IPTG_A_S1    18  566622  0.000000e+00  647    646     1   0.154799
…              …    …     …            …   …    …    …      …
494861   p3IPTG_A_S1b    18  566416  3.471600e-14  281    255    26  10.196078
495059   p3IPTG_A_S1b    18  569127  8.076710e-15  235    210    25  11.904762
443366    p3IPTG_A_S1    18  569125  0.000000e+00  534    463    71  15.334773
494831   p3IPTG_A_S1b    18  566053  0.000000e+00  271    224    47  20.982143
443009    p3IPTG_A_S1    18  566053  1.947940e-13  559    446   113  25.336323

             REF      ALT                               EFF    IMPACT  \
443045       CTA      TTT                       stop_gained      HIGH
443032         G        T                  missense_variant  MODERATE
443043         T        C                  missense_variant  MODERATE
443077         T        G                synonymous_variant       LOW
443082         A        C                       stop_gained      HIGH
…          …        …                                 …         …
494861   GGTGCGT  CGTTCTT                  missense_variant  MODERATE
495059     CAAACT    CAACT                frameshift_variant      HIGH
443366  ATCAAACT  GTCAACC  frameshift_variant&missense_variant      HIGH
494831      CGCAA    CACAT                  missense_variant  MODERATE
443009     CGCAAA   TGCTAT                       stop_gained      HIGH

          AFF                    MUT  ANN_TYPE  TOP
443045  LacI4            p.LeuGly102*  cassette    0
443032  LacI4             p.Ala145Asp  cassette    0
443043  LacI4             p.Met110Val  cassette    0
443077  LacI4              p.Ala39Ala  cassette    0
443082  LacI4                p.Tyr19*  cassette    0
…       …                        …         …    …
494861  LacI4  p.HisAlaPro86GlnGluArg  cassette    1
495059  cas9B             p.Phe897fs  cassette    1
443366  cas9B             p.Lys896fs  cassette    1
494831  LacI4      p.LeuArg208MetCys  cassette    1
443009  LacI4           p.LeuArg208*  cassette    1

[1237 rows x 16 columns]
```

[681]:
```python
data2[(data2['ANN_TYPE']=='cassette') & (data2['FRAC']>10)]
```

[681]:
```
          SAMPLE  PASS     POS          QUAL  TOT  REFN  ALTN          FRAC  \
54232    p2_6_S2     2  566587  3.889600e-06    4     3     1     33.333332
163230   p2_6_S4     2  780968  4.857580e+03  156     1   155  15500.000000
163253   p2_6_S4     2  781179  6.057650e-15   80    71     9     12.676056
163342   p2_6_S4     2  786151  5.734500e+03  182     1   181  18100.000000
163362   p2_6_S4     2  786347  0.000000e+00   14    11     3     27.272728
225311   p3_B_S1     3  780968  6.689500e+03  214     2   212  10600.000000
277012   p3_B_S2     3  780964  5.288950e+03  170     2   168   8400.000000
```

```
277062    p3_B_S2     3  786151  7.229180e+03  228      1   227  22700.000000
413818  p15_7_S5     15  780968  3.700950e+03  118      1   117  11700.000000
413844  p15_7_S5     15  785418  4.709100e-15    7      6     1     16.666666
413909  p15_7_S5     15  786347  6.060810e-12   10      6     4     66.666664

                           REF                    ALT                    EFF  \
54232                        G                      A       missense_variant
163230                   AGTCC                  CGTCC  upstream_gene_variant
163253  GACCCAACTGCCACGAAGTTTT  GACCCAACTGCCTTGATGTTAT  upstream_gene_variant
163342                     TCT                    TTT     synonymous_variant
163362                       A                      G       missense_variant
225311                   AGTCC                  CGTCC  upstream_gene_variant
277012               GGCTAGTCC              GGCTCGTCC  upstream_gene_variant
277062                  TCTGTG                 TTTGTG     synonymous_variant
413818                   AGTCC                  CGTCC  upstream_gene_variant
413844                       A                      C     synonymous_variant
413909                       A                      G       missense_variant

          IMPACT                    AFF                                 MUT  \
54232   MODERATE             cas1 LacI4                         p.Thr31Ile
163230  MODIFIER  cas2 regulator gRNA5                          c.-4520T>G
163253  MODIFIER  cas2 regulator gRNA2  c.-4751_-4743delAAACTTCGTinsTAACATCAA
163342       LOW               cas2 cat                        p.Val195Val
163362  MODERATE             cas2 lacI4                         p.Tyr24Cys
225311  MODIFIER  cas2 regulator gRNA5                          c.-4520T>G
277012  MODIFIER  cas2 regulator gRNA5                          c.-4520T>G
277062       LOW               cas2 cat                        p.Val195Val
413818  MODIFIER  cas2 regulator gRNA5                          c.-4520T>G
413844       LOW              cas2 cas9B                      p.Thr1339Thr
413909  MODERATE             cas2 lacI4                         p.Tyr24Cys

        ANN_TYPE
54232   cassette
163230  cassette
163253  cassette
163342  cassette
163362  cassette
225311  cassette
277012  cassette
277062  cassette
413818  cassette
413844  cassette
413909  cassette
```

[661]:

```
selected[selected['IMPACT']=='HIGH']
```

[661]:

	SAMPLE	PASS	POS	QUAL	TOT	REFN	ALTN	FRAC	\
494840	p3IPTG_A_S1b	18	566136	0.000000e+00	243	238	5	2.100840	
494818	p3IPTG_A_S1b	18	565929	6.208090e-15	232	227	5	2.202643	
443663	p3IPTG_A_S1	18	571681	0.000000e+00	481	469	12	2.558635	
495218	p3IPTG_A_S1b	18	571681	7.895900e-15	191	182	9	4.945055	
495059	p3IPTG_A_S1b	18	569127	8.076710e-15	235	210	25	11.904762	
443366	p3IPTG_A_S1	18	569125	0.000000e+00	534	463	71	15.334773	
443009	p3IPTG_A_S1	18	566053	1.947940e-13	559	446	113	25.336323	

	REF	ALT	\
494840	TAAGCGGGTCCCATCTTCGT	TTAGCCGGTCCCATCTTCGT	
494818	AACAATCCCCTCATTTAA	AACAATCCCCTTTTTTTA	
443663	ATTTTTTTTGATA	ATTTTTTTGATA	
495218	ATTTTTTTTGATACT	ATTTCTTTAGAAACA	
495059	CAAACT	CAACT	
443366	ATCAAACT	GTCAACC	
443009	CGCAAA	TGCTAT	

	EFF	IMPACT	AFF	MUT	\
494840	stop_gained	HIGH	LacI4	p.ArgLeu180*	
494818	stop_gained	HIGH	LacI4	p.LeuAsnGlu245*	
443663	frameshift_variant	HIGH	cas9B	p.Asn46fs	
495218	stop_gained	HIGH	cas9B	p.SerIleLysLys42*	
495059	frameshift_variant	HIGH	cas9B	p.Phe897fs	
443366	frameshift_variant&missense_variant	HIGH	cas9B	p.Lys896fs	
443009	stop_gained	HIGH	LacI4	p.LeuArg208*	

	ANN_TYPE
494840	cassette
494818	cassette
443663	cassette
495218	cassette
495059	cassette
443366	cassette
443009	cassette

1.6 NOT USED

[635]:

```
subdf1[(subdf1['FRAC']>=25) & (subdf1['ANN_TYPE']=='intergenic')]
```

[635]:

	SAMPLE	PASS	POS	QUAL	TOT	REFN	ALTN	FRAC	\
20557	p2_2_S1	2	629173	1.383230e-14	30	17	13	76.470589	
33962	p2_2_S2	2	113969	2.790510e-07	6	4	2	50.000000	
35494	p2_2_S2	2	141270	4.924100e-14	59	40	19	47.500000	
57248	p2_2_S2	2	528761	2.688110e-14	45	33	12	36.363636	
63072	p2_2_S2	2	629170	2.953720e-15	57	38	19	50.000000	

85859	p2_2_S3	2	141267	1.219870e-13	159	97	62	63.917526
161975	p3_D_S2	3	195424	4.551630e-13	413	239	174	72.803345
214268	p3_D_S2	3	629169	2.384300e-13	355	212	143	67.452827
274642	p3_D_S3	3	528755	8.763400e-14	140	84	56	66.666664
280252	p3_D_S3	3	618608	8.492520e-09	7	5	2	40.000000
406624	p3IPTG_A_S1	18	141270	2.114290e-13	235	158	77	48.734177
447613	p3IPTG_A_S1	18	618608	2.102690e-14	9	7	2	28.571428
448420	p3IPTG_A_S1	18	629167	7.256610e-14	246	139	107	76.978416
493008	p3IPTG_A_S1b	18	528758	4.628800e-14	80	44	36	81.818184

	REF	\
20557	GTTTTTTTTTTTTTTTTAGTTTGAAC	
33962	T	
35494	CAGAGAGAGAGAGAGAGAGAGC	
57248	GTTTTTTTTTTTTTTTTTGAAAGA	
63072	CTAGTTTTTTTTTTTTTTTTAGTTTGAAC	
85859	TCTCAGAGAGAGAGAGAGAGAGAGC	
161975	TTTCCAAAAAAAAAAAAAAAGTAAAATAGAAAAGC	
214268	TCTAGTTTTTTTTTTTTTTTTAGTTTGAAC	
274642	TCAAACGTTTTTTTTTTTTTTTTTGAAAGAAATTGATTGCT	
280252	A	
406624	CAGAGAGAGAGAGAGAGAGAGC	
447613	A	
448420	ATTCTAGTTTTTTTTTTTTTTTTAGTTTGAACTCAAAA	
493008	AACGTTTTTTTTTTTTTTTTTGAAAGA	

	ALT	EFF	\
20557	GTTTTTTTTTATTTATAAGTTTGAAC	upstream_gene_variant	
33962	G	upstream_gene_variant	
35494	CAGAGAGAGAGAGAGAGAGC	upstream_gene_variant	
57248	GTTTTTTTTTTTTTTTTGAAAGA	upstream_gene_variant	
63072	TTAGATTTTTTTTTTTTTTTTAGTTTGAAC	upstream_gene_variant	
85859	ACTCAGAGAGAGAGAGAGAGAGAGAGC	upstream_gene_variant	
161975	ATTCTGAAAAAAAAAAAAAAAAGTAAAATAGAAAAGC	upstream_gene_variant	
214268	TCTAGTTTTTTTTTTTTCTATAGTTTGAAC	upstream_gene_variant	
274642	CCAATCGTTTTTTTTTTTTTTTTTGAAAGAAATTGATTGCT	upstream_gene_variant	
280252	G	upstream_gene_variant	
406624	CAGAGAGAAAAAGAGAGAGAGC	upstream_gene_variant	
447613	G	upstream_gene_variant	
448420	TTTTTAATTTTTTTTTTTTTTAGTTTGAACTCAAAA	upstream_gene_variant	
493008	ACCCTTTTTTTTTTTTTTTTTGAAAGA	upstream_gene_variant	

	IMPACT	AFF	MUT	\
20557	MODIFIER	MPN508	c.-2605_-2599delAAAAAAAinsTATAAAT	
33962	MODIFIER	MPN094	c.-2292T>G	
35494	MODIFIER	MPN109	c.-624_-623delAG	
57248	MODIFIER	MPN435	c.-3956delA	

```
63072   MODIFIER  MPN508  c.-2614_-2586delGTTCAAACTAAAAAAAAAAAAAAAAACTAGi…
85859   MODIFIER  MPN109  c.-646_-622delTCTCAGAGAGAGAGAGAGAGAGAGCinsACTC…
161975  MODIFIER  MPN148  c.-97_-63delTTTCCAAAAAAAAAAAAAAAGTAAAATAGAAAAG…
214268  MODIFIER  MPN508                         c.-2604_-2602delAAAinsTAG
274642  MODIFIER  MPN435                     c.-3937_-3933delTTTGAinsATTGG
280252  MODIFIER  MPN504                                         c.-702A>G
406624  MODIFIER  MPN109                           c.-635_-633delGAGinsAAA
447613  MODIFIER  MPN504                                         c.-702A>G
448420  MODIFIER  MPN508  c.-2620_-2583delTTTTGAGTTCAAACTAAAAAAAAAAAAAAA…
493008  MODIFIER  MPN435                         c.-3939_-3937delCGTinsGGG

          ANN_TYPE  log2Frac Condition  annotation
20557   intergenic  6.256833         2  intergenic
33962   intergenic  5.643856         2  intergenic
35494   intergenic  5.569856         2  intergenic
57248   intergenic  5.184424         2  intergenic
63072   intergenic  5.643856         2  intergenic
85859   intergenic  5.998140         2  intergenic
161975  intergenic  6.185933         3  intergenic
214268  intergenic  6.075807         3  intergenic
274642  intergenic  6.058894         3  intergenic
280252  intergenic  5.321928         3  intergenic
406624  intergenic  5.606862     3IPTG  intergenic
447613  intergenic  4.836501     3IPTG  intergenic
448420  intergenic  6.266382     3IPTG  intergenic
493008  intergenic  6.354350     3IPTG  intergenic
```

[632]:

```
# For genes
plt.figure()
plt.subplot(2,1,1)
sns.swarmplot(x='IMPACT', y='FRAC', hue='PASS', data=subdf1[(subdf1['FRAC']>=5)␣
 ↪& (subdf1['ANN_TYPE']=='gene')])
plt.xticks(rotation=45, ha='right')

plt.subplot(2,1,2)
sns.swarmplot(x='IMPACT', y='FRAC', hue='PASS',data=subdf2[(subdf2['FRAC']>=5)␣
 ↪& (subdf2['ANN_TYPE']=='gene')])
plt.xticks(rotation=45, ha='right')


# For intergenic
plt.figure()
plt.subplot(2,1,1)
sns.swarmplot(x='IMPACT', y='FRAC', hue='PASS',data=subdf1[(subdf1['FRAC']>=5)␣
 ↪& (subdf1['ANN_TYPE']=='intergenic')])
plt.xticks(rotation=45, ha='right')
```

```
plt.subplot(2,1,2)
sns.swarmplot(x='IMPACT', y='FRAC', hue='PASS',data=subdf2[(subdf2['FRAC']>=5)␣
↪& (subdf2['ANN_TYPE']=='intergenic')])
plt.xticks(rotation=45, ha='right')
```

```
Canvas(toolbar=Toolbar(toolitems=[('Home', 'Reset original view', 'home', 'home'), ('Back', 'Ba
```

```
Canvas(toolbar=Toolbar(toolitems=[('Home', 'Reset original view', 'home', 'home'), ('Back', 'Ba
```

[632]:
```
(array([0, 1]), <a list of 2 Text xticklabel objects>)
```

[288]:
```
def plot_fixation(df):
    """ Plot to show the percentage of variants mapping to each type of␣
↪annotation """
    colord = {'intergenic':0, 'gene':1, 'essential':2, 'non-essential':3,␣
↪'cassette':4}
    pasind = {2:0, 3:1, 15:2, 18:3}
    rs = {}
    colors = []
    for k, v in Counter(list(df['POS'])).items():
        if v>2:
            rs[k] = [0.0,0.0,0.0,0.0]
            subdf = df[df['POS']==k]
            for passage in set(subdf['PASS']):
                rs[k][pasind[passage]] =␣
↪list(subdf[subdf['PASS']==passage]['FRAC'])
    return rs
dic  = plot_fixation(data1)
```

[327]:
```
rs = {}
columns = ['p2_2_S1', 'p2_2_S2', 'p2_2_S3', 'p3_D_S2', 'p3_D_S3', 'p15_F_S1',␣
↪'p15_F_S5',  'p3IPTG_A_S1', 'p3IPTG_A_S1b']
for n in columns:
    rs[n] = []
    for m in columns:
        rs[n].append(round(mannwhitneyu(data1[(data1['SAMPLE']==n) &␣
↪(data1['FRAC']>=1)]['FRAC'], data1[(data1['SAMPLE']==m) &␣
↪(data1['FRAC']>=1)]['FRAC'])[1], 8))
pvalues = pd.DataFrame.from_dict(rs, orient='index')
pvalues.columns=columns
pvalues
```

[327]:
```
              p2_2_S1   p2_2_S2   p2_2_S3   p3_D_S2   p3_D_S3  p15_F_S1  \
p2_2_S1           0.5  0.000000  0.000000  0.000000  0.000000  0.000000
```

```
p2_2_S2          0.0  0.500000  0.000000  0.000000  0.000000  0.304095
p2_2_S3          0.0  0.000000  0.499998  0.000000  0.368914  0.000000
p3_D_S2          0.0  0.000000  0.000000  0.499997  0.000000  0.000000
p3_D_S3          0.0  0.000000  0.368914  0.000000  0.499996  0.000000
p15_F_S1         0.0  0.304095  0.000000  0.000000  0.000000  0.500000
p15_F_S5         0.0  0.000000  0.000000  0.481911  0.000000  0.000000
p3IPTG_A_S1      0.0  0.000000  0.297108  0.000000  0.370263  0.000000
p3IPTG_A_S1b     0.0  0.000000  0.000005  0.000000  0.000963  0.000000

              p15_F_S5  p3IPTG_A_S1  p3IPTG_A_S1b
p2_2_S1       0.000000     0.000000      0.000000
p2_2_S2       0.000000     0.000000      0.000000
p2_2_S3       0.000000     0.297108      0.000005
p3_D_S2       0.481911     0.000000      0.000000
p3_D_S3       0.000000     0.370263      0.000963
p15_F_S1      0.000000     0.000000      0.000000
p15_F_S5      0.499997     0.000000      0.000000
p3IPTG_A_S1   0.000000     0.499991      0.008616
p3IPTG_A_S1b  0.000000     0.008616      0.499999
```

[352]:
```
def extracta(df, sample):
    rs = []
    subdf = df[df['SAMPLE']==sample].copy()
    fractions = {}
    for k, v in zip(subdf['POS'], subdf['FRAC']):
        if k in fractions:
            fractions[k].append(v)
        else:
            fractions[k] = [v]
    fractions = {k:np.mean(v) for k, v in fractions.items()}
    for i in range(1, len(genome_C5)+1):
        if i in fractions:
            if fractions[i]>=1:
                rs.append(i)
            else:
                rs.append(1)
    return np.array(rs)

def volcano():
    rs = {}
    for sample in set(data1[data1['PASS'].isin([3,18])]['SAMPLE']):
         rs[sample] = extracta(data1, sample)
    fcs = []
    pvals = []
    positions = []
    for i in range(1, len(genome_C5)+1):
        try:
```

```
            vals = np.array([rs['p3IPTG_A_S1b'][i], rs['p3IPTG_A_S1'][i],␣
↪rs['p3_D_S3'][i], rs['p3_D_S2'][i]])
            if len(vals[vals>1])==0:
                pass
            else:
                fc = np.log2(np.mean([rs['p3IPTG_A_S1b'][i],␣
↪rs['p3IPTG_A_S1'][i]]))-np.log2(np.mean([rs['p3_D_S3'][i],␣
↪rs['p3_D_S2'][i]]))
                pval = -1*np.log10(mannwhitneyu([rs['p3IPTG_A_S1b'][i],␣
↪rs['p3IPTG_A_S1'][i]], [rs['p3_D_S3'][i], rs['p3_D_S2'][i]])[1])
                fcs.append(fc)
                pvals.append(pval)
                positions.append(i)
        except:
            pass
    return fcs, pvals, positions
```

[353]:

```
f, pv, p  = volcano()
```

[357]:

```
plt.figure()
plt.scatter(f, pv)
plt.axhline(y=-1*np.log10(0.05), linestyle='--', color='r')
```

```
/home/smiravet/.local/lib/python3.6/site-packages/ipykernel_launcher.py:1:
RuntimeWarning: More than 20 figures have been opened. Figures created through
the pyplot interface (`matplotlib.pyplot.figure`) are retained until explicitly
closed and may consume too much memory. (To control this warning, see the
rcParam `figure.max_open_warning`).
  """Entry point for launching an IPython kernel.
```

```
Canvas(toolbar=Toolbar(toolitems=[('Home', 'Reset original view', 'home', 'home'), ('Back', 'Ba
```

[357]:

```
<matplotlib.lines.Line2D at 0x7fef2ff662b0>
```

[601]:

```
background = np.log2(data1[(data1['PASS']==3) & (data1['FRAC']>=1)]['FRAC'])
plt.figure()
sns.distplot(background)
plt.axvline(x=np.mean(background))
plt.axvline(x=np.percentile(background,99), color='r', linestyle='--')
selected = data1[data1['PASS']==18].copy()
#selected['pvalue'] = [mannwhitneyu(frac, background) for frac in␣
↪list(selected['FRAC'])]
```

```
/home/smiravet/.local/lib/python3.6/site-packages/ipykernel_launcher.py:2:
RuntimeWarning: More than 20 figures have been opened. Figures created through
the pyplot interface (`matplotlib.pyplot.figure`) are retained until explicitly
```

```
closed and may consume too much memory. (To control this warning, see the
rcParam `figure.max_open_warning`).
```

```
Canvas(toolbar=Toolbar(toolitems=[('Home', 'Reset original view', 'home', 'home'), ('Back', 'Ba
```

[250]:
```
new_dic = {}
for k, v in dic.items():
    a = np.array(v)
    try:
        if len(a[1])==2 and len(a[-1])==2:
            new_dic[k] = a
    except:
        pass
```

[270]:
```
x3, x3i, x15 = [[], [], []]
positions = []
for k, v in dic.items():
    a = np.array(v)
    try:
        if len(a[0])==2:
            if len(a[1])==2:
                x3.append(np.mean(a[1])/np.mean(a[0]))
            elif len(a[2])==2:
                x15.append(np.mean(a[2])/np.mean(a[0]))
            elif len(a[-1])==2:
                positions.append(k)
                x3i.append(np.mean(a[-1])/np.mean(a[0]))
            else:
                pass
    except:
        pass
```

[271]:
```
plt.figure()
plt.violinplot([x3, x15, x3i])
plt.xticks([1,2,3],['P3/P2', 'P15/P2', 'P3-IPTG/P2'])
plt.axhline(y=np.mean(x3)+2*np.std(x3), color='red', linestyle='--', label='95%␣
↪threshold P3/P2')
```

```
Canvas(toolbar=Toolbar(toolitems=[('Home', 'Reset original view', 'home', 'home'), ('Back', 'Ba
```

[271]:
```
<matplotlib.lines.Line2D at 0x7fef462b1e10>
```

[274]:
```
thr = np.mean(x3)+2*np.std(x3)
thr
```

[274]:
```
1.0411993871078709
```

[279]:
```
a = np.array(x3i)
b = np.array(positions)
```

[282]:
```
b[a>=thr]
```

[282]:
```
array([211633, 264869, 198159, 224676, 254989, 458314, 553466, 675401,
       767918, 807060])
```

1.7 3. Fixation of the mutations

In this section we evaluate which mutations increase their representation from passage 2 to 3 and comparing this ratio between non-treated and induced with IPTG samples.

[115]:
```
data1[(data1['ALTN']>10) & (data1['PASS']==3)]
```

[115]:
```
         SAMPLE  PASS     POS          QUAL  TOT  REFN  ALTN       FRAC  \
137985  p3_D_S2     3    6772  1.989160e-15  582   569    13   2.284710
138227  p3_D_S2     3    8563  0.000000e+00  523   512    11   2.148438
139095  p3_D_S2     3   15285  4.221290e-16  684   673    11   1.634472
139787  p3_D_S2     3   20319  2.260140e-14  599   588    11   1.870748
140828  p3_D_S2     3   28083  0.000000e+00  644   633    11   1.737757
…           …    …      …        …   …    …    …         …
267794  p3_D_S3     3  414743  2.170210e-15  172   161    11   6.832298
269396  p3_D_S3     3  442878  0.000000e+00  235   222    13   5.855856
274642  p3_D_S3     3  528755  8.763400e-14  140    84    56  66.666664
280845  p3_D_S3     3  629170  0.000000e+00  175   104    71  68.269234
286599  p3_D_S3     3  712979  0.000000e+00  228   216    12   5.555555

                                             REF  \
137985                             TCTTTAAGGAAAA
138227          AAACCAAAATAAACCAATGAAACAAGGCGTGT
139095                           CCCTAAAGAACAAGG
139787                            CCCGTTCCCGCGTG
140828                           AATCCACGGCCGTTT
…                                            …
267794                                         T
269396                                   TAGTGGT
274642  TCAAACGTTTTTTTTTTTTTTTTTGAAAGAAATTGATTGCT
280845          CTAGTTTTTTTTTTTTTTTTAGTTTGAACTCA
286599                             ACCCCCCCCGCTA

                                             ALT                     EFF  \
137985                             ACTTAAAGGAAAA        missense_variant
138227          AAACCAAAATAATCTATTGAAACAAGGCGTGT  initiator_codon_variant
139095                           TCCTGAAGAACAAGG        missense_variant
139787                            CCCGTTCCCGAGAG        missense_variant
```

```
140828                             AATCCACGGCTGTCT        missense_variant
…                                                …                       …
267794                                           A     synonymous_variant
269396                                     TGGAGGT       missense_variant
274642  CCAATCGTTTTTTTTTTTTTTTTTGAAAGAAATTGATTGCT   upstream_gene_variant
280845          TTAGCTTTTTTTTTTTTTTTTTAGTTTGAACTCA   upstream_gene_variant
286599                                ACCCCCCCGCTA   upstream_gene_variant

          IMPACT     AFF                                             MUT  \
137985  MODERATE  MPN004                             p.LeuPhe651HisLeu
138227       LOW  MPN006                                       p.Met1?
139095  MODERATE  MPN013                                   p.Lys100Glu
139787  MODERATE  MPN018                                   p.Val336Glu
140828  MODERATE  MPN022                             p.ArgPhe260CysLeu
…            …       …                                             …
267794       LOW  MPN347                                   p.Gly268Gly
269396  MODERATE  MPN370                                   p.Val544Glu
274642  MODIFIER  MPN435                   c.-3937_-3933delTTTGAinsATTGG
280845  MODIFIER  MPN508  c.-2617_-2586delTGAGTTCAAACTAAAAAAAAAAAAAAAAACT…
286599  MODIFIER  MPN579                                     c.-4224delG

          ANN_TYPE
137985        gene
138227        gene
139095        gene
139787        gene
140828        gene
…              …
267794        gene
269396        gene
274642  intergenic
280845  intergenic
286599  intergenic

[68 rows x 15 columns]
```

[116]:

```
Canvas(toolbar=Toolbar(toolitems=[('Home', 'Reset original view', 'home', 'home'), ('Back', 'Ba
```

[116]: <matplotlib.axes._subplots.AxesSubplot at 0x7fa3e0a23a90>

[40]:

```python
def plot_fixation(df):

    subdf = df.groupby(['PASS', 'POS', 'ALT']).sum()
    return subdf
```

```
    positions = set([k for k, v in Counter(subdf['POS']).items() if v>=3])
    return df[df['POS'].isin(positions)]
```

[57]:
```
np.array([i for i in plot_fixation(snpcalls1).index])
```

[57]:
```
array([['2', '6', 'T'],
       ['2', '13', 'C'],
       ['2', '31', 'A'],
       …,
       ['18', '822913', 'TGCGTGAAA'],
       ['18', '822925', 'C'],
       ['18', '822931', 'CAAGTTCT']], dtype='<U73')
```

[196]:
```
plt.figure(figsize=(10,10))
sns.scatterplot(x='POS', y='FRAC', hue='PASS',␣
↪data=effect1[(effect1['IMPACT']=='HIGH') & (effect1['FRAC']>=1)])
```

```
Canvas(toolbar=Toolbar(toolitems=[('Home', 'Reset original view', 'home', 'home'), ('Back', 'Ba
```

[196]:
```
<matplotlib.axes._subplots.AxesSubplot at 0x7f1bd89350f0>
```

[80]:
```
plt.figure()
effect21['logFRAC'] = np.log2(effect21['FRAC'])
sns.violinplot(x='IMPACT', y='FRAC', hue='PASS', data=effect1)
```

```
/home/smiravet/.local/lib/python3.6/site-packages/ipykernel_launcher.py:1:
RuntimeWarning: More than 20 figures have been opened. Figures created through
the pyplot interface (`matplotlib.pyplot.figure`) are retained until explicitly
closed and may consume too much memory. (To control this warning, see the
rcParam `figure.max_open_warning`).
  """Entry point for launching an IPython kernel.
```

```
Canvas(toolbar=Toolbar(toolitems=[('Home', 'Reset original view', 'home', 'home'), ('Back', 'Ba
```

[80]:
```
<matplotlib.axes._subplots.AxesSubplot at 0x7f92f451a828>
```

[42]:
```
snpcalls1[snpcalls1['SAMPLE']=='p2_2_S1'].sort_values('FRAC')
```

[42]:
```
          POS                               REF  \
18482  566325                                 C
18481  566311                                 A
18485  566383                               GCC
18483  566361                                 A
18476  566244                                 A
…         …                               …
6416   195428  CAAAAAAAAAAAAAAAGTAAAATAGAAAAGC
```

```
17103  528761         GTTTTTTTTTTTTTTTTTTGAAAGA
20563  629173       GTTTTTTTTTTTTTTTTTTAGTTTGAAC
4753   141270           CAGAGAGAGAGAGAGAGAGAGC
19735  602077                                T

                                                          ALT          QUAL  TOT  \
18482                                                       T  1.469330e-15  205
18481                                                       G  7.046470e-16  198
18485                                                     CCT  0.000000e+00  181
18483                                                       G  0.000000e+00  172
18476                                                       G  1.167980e-15  171
…                                                         …             …    …
6416   CAAAATAAGAAAAAAAGTAAAATAGAAAAGC,CAAAAAAAAAAAAA…  0.000000e+00   24
17103  GTTTTTTTTTTTTTTTTTTGAAAGA,GTTTTTTTTTTTTTTTTTTTGAAAGA  0.000000e+00   28
20563  GTTTTTTTTTATTTATAAGTTTGAAC,GTTTTTTTTTTTTTTTTAGT…  1.383230e-14   30
4753       CAGAGAGAGAGAGAGAGAGAGC,CAGAGAGAGAGAGAGAGAGAGAGC  0.000000e+00   52
19735                                                       A  1.607630e+01    1

       REFN  ALTN       ALTS        FRAC   SAMPLE  P  \
18482   204     1          1    0.487805  p2_2_S1  2
18481   197     1          1    0.505051  p2_2_S1  2
18485   180     1          1    0.552486  p2_2_S1  2
18483   171     1          1    0.581395  p2_2_S1  2
18476   170     1          1    0.584795  p2_2_S1  2
…       …     …          …           …      … ..
6416     15     9    1,1,1,6   37.500000  p2_2_S1  2
17103    16    12        6,6   42.857143  p2_2_S1  2
20563    17    13  1,2,6,2,2   43.333333  p2_2_S1  2
4753     27    25       7,18   48.076923  p2_2_S1  2
19735     0     1          1  100.000000  p2_2_S1  2

                                                      EFF
18482  T|synonymous_variant|LOW|Gene_565549_566677|Ge…
18481  G|missense_variant|MODERATE|Gene_565549_566677…
18485  CCT|missense_variant|MODERATE|Gene_565549_5666…
18483  G|synonymous_variant|LOW|Gene_565549_566677|Ge…
18476  G|synonymous_variant|LOW|Gene_565549_566677|Ge…
…                                                   …
6416   CAAAATAAGAAAAAAAGTAAAATAGAAAAGC|upstream_gene_…
17103  GTTTTTTTTTTTTTTTTTTGAAAGA|upstream_gene_variant|…
20563  GTTTTTTTTTATTTATAAGTTTGAAC|upstream_gene_varia…
4753   CAGAGAGAGAGAGAGAGAGAGC|upstream_gene_variant|MOD…
19735  A|missense_variant|MODERATE|P02_orf1300|MPN489…

[27073 rows x 12 columns]
```

[46]:

[47]:
```
simplify_effect(snpcalls1, genome=20)
```

```
␣
↪---------------------------------------------------------------------------

      UnboundLocalError                           Traceback (most recent call␣
↪last)

      <ipython-input-47-d335a4bfad19> in <module>
    ----> 1 simplify_effect(snpcalls1, genome=20)


      <ipython-input-46-bf2fb86f217b> in simplify_effect(df, positions,␣
↪genome, modify_original, impact, mintot, minrefn, minaltn)
          7         annotations = correspondance(genome=genome,␣
↪positions=positions)
          8     else:
    ----> 9         annotations
         10     return annotations
         11     annotations = {v.qualifiers['label'][0]:[int(v.location.start),␣
↪int(v.location.end), '-'] for k, v in annotations.items() if 'label' in v.
↪qualifiers}


      UnboundLocalError: local variable 'annotations' referenced before␣
↪assignment
```

[45]:
```
# Figure


def plot_analysis(df):
    repro = [k for k, v in Counter(list(df.POS)).items() if v>=2]
    plt.figure()
    plt.subplot(3,3,1)
    sns.lineplot(x='P', y='FRAC', data=df[(df['POS'].isin(posE)) & (df['POS'].
 ↪isin(repro))], label='E')
    sns.lineplot(x='P', y='FRAC', data=df[(df['POS'].isin(posN)) & (df['POS'].
 ↪isin(repro))], label='NE')
    sns.lineplot(x='P', y='FRAC', data=df[(df['POS'].isin(cas1)) & (df['POS'].
 ↪isin(repro))], label='C')


plot_analysis(snpcalls1)
```

```
Canvas(toolbar=Toolbar(toolitems=[('Home', 'Reset original view', 'home', 'home'), ('Back', 'Ba
```

1.8 4. Substituion rate per base comparing cassette versus other distributions

To evaluate the selection in the cassette we compare the rate of substitutions per base occuring within the cassette, a set of essential and non-essential genes and with the general distribution observed at genome level. In this case we do not care about the fraction each variant is found but how many times a variant is found in the cassette versus the other distributions.

[11]:
```
# Show the ratio of mutation in cassette versus general
def mutation_rate(snpdf, segment, mintot=5, minrefn=3, minaltn=2):
    _df = snpdf[(snpdf['TOT']>=mintot) & (snpdf['REFN']>=minrefn) &␣
↪(snpdf['ALTN']>=minaltn)].copy()
    rs = {}
    for sample in set(_df.SAMPLE):
        total_len = 816394
        _df2 = _df[_df['SAMPLE']==sample].copy()
        NE = _df2[_df2['POS'].isin(posN)]
        ES = _df2[_df2['POS'].isin(posE)]
        TG = _df2[~_df2['POS'].isin(segment)]
        CS = _df2[_df2['POS'].isin(segment)]
        #total_len+=len(segment)
        rs[sample] = [sample, sample.split('_')[0],
                      int(sample.split('_')[0].replace('p', '').
↪replace('3IPTG', '18')),
                      TG.shape[0], ES.shape[0], NE.shape[0], CS.shape[0],
                      sum(TG['FRAC']), sum(ES['FRAC']), sum(NE['FRAC']),␣
↪sum(CS['FRAC'])]
    jj = pd.DataFrame.from_dict(rs, orient='index')
    jj.columns = ['SAMPLE', 'COND', 'P', 'T', 'E', 'N', 'C', 'TF', 'EF', 'NF',␣
↪'CF']
    jj['T%'] = 100*jj['T']/(total_len)
    jj['E%'] = 100*jj['E']/len(posE)
    jj['N%'] = 100*jj['N']/len(posN)
    jj['C%'] = 100*jj['C']/len(segment)
    return jj

def tp(df, order):
    rs = {}
    c=0
    for row, col in df.iterrows():
        rs[c] = [col['COND'],'Essential', col['E%'], col['EF']]
        rs[c+1] = [col['COND'],'Non-essential', col['N%'], col['NF']]
        rs[c+2] = [col['COND'],'Chromosome', col['T%'], col['TF']]
        rs[c+3] = [col['COND'],'Cassette', col['C%'], col['CF']]
        c+=4
    a = pd.DataFrame.from_dict(rs, orient='index')
```

```
    a.columns = ['Condition', 'Loci', 'Variation per base [%]', 'Accumulated␣
↪Fraction [%]']
    return orderdf(a, ordered_classes=order, col='Condition')
```

[32]:
```
mutrate11 = mutation_rate(snpcalls1, segment=cas1)
mutrate21 = mutation_rate(snpcalls2, segment=cas1)
mutrate22 = mutation_rate(snpcalls2, segment=cas2)
mutrate212 = mutation_rate(snpcalls2, segment=cas12)
```

[33]:
```
plt.close('all')
plt.figure(figsize=(15, 3))
c = 1
for text, df in zip(['C5 Par', 'C20 Par', 'C20 3b'], [mutrate11, mutrate21,␣
↪mutrate22]):
    plt.subplot(1,3,c)
    if text=='C5 Par':
        sns.barplot(x='Condition', y='Variation per base [%]', hue='Loci',␣
↪palette='mako', data=tp(df, ['p2','p3','p15', 'p3IPTG']))
    else:
        sns.barplot(x='Condition', y='Variation per base [%]', hue='Loci',␣
↪palette='mako', data=tp(df, ['p2','p3','p15']))
    plt.ylim(0, 15)
    c+=1
plt.tight_layout()
```

```
Canvas(toolbar=Toolbar(toolitems=[('Home', 'Reset original view', 'home', 'home'), ('Back', 'Ba
```

[27]:
```
from dnds_functions import evolution_of_sample, dnds
ttt = evolution_of_sample(effect1[effect1['IMPACT']=='HIGH'])
```

[40]:
```
effectC= simplify_effect(snpcalls1, cas1, minaltn=3)
effectE= simplify_effect(snpcalls1, posE, minaltn=3)
effectN= simplify_effect(snpcalls1, posN, minaltn=3)

tttC = evolution_of_sample(effectC)
tttE = evolution_of_sample(effectE)
tttN = evolution_of_sample(effectN)
```

[53]:
```
tttE
```

[53]:
```
{'p15_F_S1': [0.015,
  0.011,
  1.3636363636363638,
  0.015,
  0.011,
  1.3636363636363638],
```

```
'p3IPTG_A_S1': [0.418,
 0.265,
 1.5773584905660376,
 0.612,
 0.327,
 1.8715596330275228],
'p3_D_S3': [0.012, 0.0, '+', 0.012, 0.0, '+'],
'p2_2_S1': [0, 0, '/', 0, 0, '/'],
'p3IPTG_A_S1b': [0, 0, '/', 0, 0, '/'],
'p15_F_S5': [0, 0, '/', 0, 0, '/'],
'p2_2_S3': [0.009000000000000001,
 0.022,
 0.40909090909090917,
 0.009000000000000001,
 0.022,
 0.40909090909090917],
'p2_2_S2': [0.006, 0.0, '+', 0.006, 0.0, '+'],
'p3_D_S2': [0.03,
 0.06599999999999999,
 0.4545454545454546,
 0.03,
 0.06599999999999999,
 0.4545454545454546]}
```

[55]:
```
plt.figure()
x, y = [[], []]
for k, v in tttC.items():
    if type(v[-1])==float and type(tttE[k][-1])==float:
        x.append(tttE[k][-1])
        y.append(v[-1])
plt.scatter(x, y)
```

```
Canvas(toolbar=Toolbar(toolitems=[('Home', 'Reset original view', 'home', 'home'), ('Back', 'Ba
```

[55]:
```
<matplotlib.collections.PathCollection at 0x7f92ec6b1be0>
```

[307]:
```
plt.figure()
plt.subplot(2,2,1)
sns.countplot(x='SAMPLE', data=snpcalls1)
plt.xticks(rotation=45, ha='right')
plt.title('Variant count per sample')

plt.subplot(2,2,2)
sns.boxplot(x='SAMPLE', y='FRAC', data=snpcalls1)
plt.xticks(rotation=45, ha='right')
plt.title('Fraction distribution per sample')
```

```
plt.subplot(2,2,3)
sns.countplot(x='SAMPLE', data=snpcalls2)
plt.xticks(rotation=45, ha='right')
plt.title('Variant count per sample')

plt.subplot(2,2,4)
sns.boxplot(x='SAMPLE', y='FRAC', data=snpcalls2)
plt.xticks(rotation=45, ha='right')
plt.title('Fraction distribution per sample')

plt.tight_layout()
```

```
Canvas(toolbar=Toolbar(toolitems=[('Home', 'Reset original view', 'home', 'home'), ('Back', 'Ba
```

General exploration of the most representative variants:

[308]:
```
plt.figure(figsize=(15, 10))
plt.subplot(3,1,1)
sns.scatterplot(x='POS', y='FRAC', hue='SAMPLE',␣
 ↪data=snpcalls1[(snpcalls1['FRAC']>=5) & (snpcalls1['TOT']>=10) &␣
 ↪(snpcalls1['P']<=15) & (snpcalls1['FRAC']<100)])
plt.axvspan(565510, 572114, color='gray', alpha=0.2)
plt.xlabel('')
plt.ylabel('Fraction of variant reads [%]')
plt.xlim(1, 890000)

plt.subplot(3,1,2)
sns.scatterplot(x='POS', y='FRAC', hue='SAMPLE',␣
 ↪data=snpcalls1[(snpcalls1['FRAC']>=5) & (snpcalls1['TOT']>=10) &␣
 ↪(snpcalls1['P']>15) & (snpcalls1['FRAC']<100)])
plt.axvspan(565510, 572114, color='gray', alpha=0.2)
plt.xlabel('')
plt.ylabel('Fraction of variant reads [%]')
plt.xlim(1, 890000)

plt.subplot(3,1,3)
sns.scatterplot(x='POS', y='FRAC', hue='SAMPLE',␣
 ↪data=snpcalls2[(snpcalls2['FRAC']>=5) & (snpcalls2['TOT']>=10) &␣
 ↪(snpcalls2['FRAC']<100)])
plt.axvspan(cass_par[0], cass_par[1], color='gray', alpha=0.2)
plt.axvspan(cass_3b[0], cass_3b[1], color='gray', alpha=0.2)
plt.xlabel('genome position [bp]')
plt.ylabel('Fraction of variant reads [%]')
plt.xlim(1, 890000)

plt.savefig('./figures/supfig1.svg')
```

```
plt.savefig('./figures/supfig1.png')
```

```
Canvas(toolbar=Toolbar(toolitems=[('Home', 'Reset original view', 'home', 'home'), ('Back', 'Ba
```

[ ]:
```

```

[ ]:
```

```

2 Selection of variants

In this case we evaluate the fraction of each variant and how it is fixed along passages. As not all the samples are directly related, we consider the induced sample (IPTG) as 18. In this sense, if a mutation found in passages 2, 3 or 15 is found in 18 with a higher rate it implies that this variants are able to confere the capability to surpass the killswitch

[610]:
```
def plot_evolution(df):
    positions = set(df[df['FRAC']>1].POS)
    plt.figure()
    plt.subplot(1,2,1)
    sns.lineplot(x='PASS', y='FRAC', hue='POS', data=df[df['POS'].
 ↪isin(positions)])
    plt.subplot(1,2,2)
    sns.boxplot(x='PASS', y='FRAC', data=df[df['POS'].isin(positions)])
```

[611]:
```
plot_evolution(data1)
```

```
/home/smiravet/.local/lib/python3.6/site-packages/ipykernel_launcher.py:3:
RuntimeWarning: More than 20 figures have been opened. Figures created through
the pyplot interface (`matplotlib.pyplot.figure`) are retained until explicitly
closed and may consume too much memory. (To control this warning, see the
rcParam `figure.max_open_warning`).
  This is separate from the ipykernel package so we can avoid doing imports
until
```

```
Canvas(toolbar=Toolbar(toolitems=[('Home', 'Reset original view', 'home', 'home'), ('Back', 'Ba
```

```
             ␣
 ↪---------------------------------------------------------------------------

        KeyboardInterrupt                         Traceback (most recent call␣
 ↪last)

        <ipython-input-611-effa08ace5dc> in <module>
    ----> 1 plot_evolution(data1)
```

```
    <ipython-input-610-c53d1b5b5f59> in plot_evolution(df)
      3     plt.figure()
      4     plt.subplot(1,2,1)
----> 5     sns.lineplot(x='PASS', y='FRAC', hue='POS', data=df[df['POS'].
↪isin(positions)])
      6     plt.subplot(1,2,2)
      7     sns.boxplot(x='PASS', y='FRAC', data=df[df['POS'].
↪isin(positions)])


    /usr/local/lib/python3.6/dist-packages/seaborn/relational.py in␣
↪lineplot(x, y, hue, size, style, data, palette, hue_order, hue_norm, sizes,␣
↪size_order, size_norm, dashes, markers, style_order, units, estimator, ci,␣
↪n_boot, sort, err_style, err_kws, legend, ax, **kwargs)
   1082         ax = plt.gca()
   1083
-> 1084     p.plot(ax, kwargs)
   1085
   1086     return ax


    /usr/local/lib/python3.6/dist-packages/seaborn/relational.py in␣
↪plot(self, ax, kws)
    764         # Loop over the semantic subsets and draw a line for each
    765
--> 766         for semantics, data in self.subset_data():
    767
    768             hue, size, style = semantics


    /usr/local/lib/python3.6/dist-packages/seaborn/relational.py in␣
↪subset_data(self)
    330
    331             if self.sort:
--> 332                 subset_data = sort_df(subset_data, ["units", "x",␣
↪"y"])
    333
    334             if self.units is None:


    /usr/local/lib/python3.6/dist-packages/seaborn/utils.py in sort_df(df,␣
↪*args, **kwargs)
     43     """Wrapper to handle different pandas sorting API pre/post 0.17.
↪"""
     44     try:
```

```
---> 45         return df.sort_values(*args, **kwargs)
     46     except AttributeError:
     47         return df.sort(*args, **kwargs)


    /usr/local/lib/python3.6/dist-packages/pandas/core/frame.py in␣
↪sort_values(self, by, axis, ascending, inplace, kind, na_position)
   5001
   5002         new_data = self._data.take(
-> 5003             indexer, axis=self._get_block_manager_axis(axis),␣
↪verify=False
   5004         )
   5005


    /usr/local/lib/python3.6/dist-packages/pandas/core/internals/managers.py␣
↪in take(self, indexer, axis, verify, convert)
   1395         new_labels = self.axes[axis].take(indexer)
   1396         return self.reindex_indexer(
-> 1397             new_axis=new_labels, indexer=indexer, axis=axis,␣
↪allow_dups=True
   1398         )
   1399


    /usr/local/lib/python3.6/dist-packages/pandas/core/internals/managers.py␣
↪in reindex_indexer(self, new_axis, indexer, axis, fill_value, allow_dups, copy)
   1265                     ),
   1266                 )
-> 1267                 for blk in self.blocks
   1268             ]
   1269


    /usr/local/lib/python3.6/dist-packages/pandas/core/internals/managers.py␣
↪in <listcomp>(.0)
   1265                     ),
   1266                 )
-> 1267                 for blk in self.blocks
   1268             ]
   1269


    /usr/local/lib/python3.6/dist-packages/pandas/core/internals/blocks.py␣
↪in take_nd(self, indexer, axis, new_mgr_locs, fill_tuple)
   1312
   1313         new_values = algos.take_nd(
```

```
-> 1314             values, indexer, axis=axis, allow_fill=allow_fill,␣
↪fill_value=fill_value
   1315         )
   1316


/usr/local/lib/python3.6/dist-packages/pandas/core/algorithms.py in␣
↪take_nd(arr, indexer, axis, out, fill_value, mask_info, allow_fill)
   1649     if is_extension_array_dtype(arr):
   1650         return arr.take(indexer, fill_value=fill_value,␣
↪allow_fill=allow_fill)
-> 1651     elif is_datetime64tz_dtype(arr):
   1652         return arr.take(indexer, fill_value=fill_value,␣
↪allow_fill=allow_fill)
   1653     elif is_interval_dtype(arr):


KeyboardInterrupt:
```

[182]:

```
annotations
```

[182]:

```
{'LacI4': [565549, 566678, '-'],
 'pS': [566678, 566721, '-'],
 'Par cassette': [566747, 567674, '-'],
 'cas9B': [567711, 571818, '-'],
 'pG64': [571818, 571934, '-'],
 'gRNA2': [571942, 572044, '-'],
 'protospacer12 (10 targets)': [572024, 572044, '-'],
 'p438': [572044, 572066, '-'],
 'IR-OR': [572088, 572114, '-'],
 'MTn insertion point': [572113, 572124, '-']}
```

[330]:

```
from Bio.Seq import Seq
genes_affected = {}
for k, v in annotations.items():
    if k in ['cas9B', 'LacI4']:
        if k=='cas9B':
            seq = Seq(genome_C5[v[0]:v[1]])
        else:
            seq = Seq(genome_C5[v[0]:v[1]])
        print(len(seq)/3)
        rvc = seq.reverse_complement()
        mrn = rvc.transcribe()
        prt = mrn.translate(table=4)
        genes_affected[k] = [seq, rvc, mrn, prt, len(prt)]
```

```
376.3333333333333
1369.0
```

```
/home/smiravet/.local/lib/python3.6/site-packages/Bio/Seq.py:2309:
BiopythonWarning: Partial codon, len(sequence) not a multiple of three.
Explicitly trim the sequence or add trailing N before translation. This may
become an error in future.
  BiopythonWarning)
```

[333]:
```
genes_affected['LacI4'][3][-10:]
```

[333]:
```
Seq('RLESGQ*R*V', HasStopCodon(ExtendedIUPACProtein(), '*'))
```

LacI4 has extra bases in the annotation, that makes it to be non-codonic. I will consider the first stop found (move the end 10 bases)...

[33]:
```
from Bio.Seq import Seq
genes_affected = {}
for k, v in annotations.items():
    if k in ['cas9B', 'LacI4']:
        if k=='cas9B':
            seq = Seq(genome_seq[v[0]:v[1]])
        else:
            seq = Seq(genome_seq[v[0]+10:v[1]])
        print(len(seq)/3)
        rvc = seq.reverse_complement()
        mrn = rvc.transcribe()
        prt = mrn.translate(table=4)
        genes_affected[k] = [seq, rvc, mrn, prt, len(prt)]
```

```
           ␣
↪---------------------------------------------------------------------------

        NameError                                 Traceback (most recent call␣
↪last)

        <ipython-input-33-84fcf2bf1dbc> in <module>
          1 from Bio.Seq import Seq
          2 genes_affected = {}
    ----> 3 for k, v in annotations.items():
          4     if k in ['cas9B', 'LacI4']:
          5         if k=='cas9B':


        NameError: name 'annotations' is not defined
```

[34]:
```
genes_affected['LacI4'][3][-10:]
```

```
␣
↪---------------------------------------------------------------------------

    KeyError                                  Traceback (most recent call␣
↪last)

    <ipython-input-34-2cf0e14076cd> in <module>
----> 1 genes_affected['LacI4'][3][-10:]


    KeyError: 'LacI4'
```

[ ]:
```
aa
```

3 Evaluate effect of the selected mutations

[35]:
```
snpcalls1
```

[35]:
```
          POS        REF        ALT          QUAL  TOT  REFN  ALTN      FRAC  \
0          70          C          T  4.927350e-15   60    58     2  3.333333
1         149          T          C  0.000000e+00   75    74     1  1.333333
2         150          A          T  2.263910e-15   69    68     1  1.449275
3         154          T          C  0.000000e+00   62    61     1  1.612903
4         176       TAAT       CAAC  3.420820e-15   58    57     1  1.724138
…         …          …          …             …    …     …     …         …
41166  822775      AACGT      GACGC  3.631530e-15  171   170     1  0.584795
41167  822787          A          G  1.152910e-15  175   173     2  1.142857
41168  822830          T          G  3.857310e-15  165   164     1  0.606061
41169  822867      TTTTT      CTTTC  0.000000e+00  172   171     1  0.581395
41170  822913  CGCGCGTAT  TGCGTGAAA  0.000000e+00  161   160     1  0.621118

            SAMPLE   P                                                EFF
0          p2_2_S1   2  T|upstream_gene_variant|MODIFIER|dnaN|MPN001|t…
1          p2_2_S1   2  C|upstream_gene_variant|MODIFIER|dnaN|MPN001|t…
2          p2_2_S1   2  T|upstream_gene_variant|MODIFIER|dnaN|MPN001|t…
3          p2_2_S1   2  C|upstream_gene_variant|MODIFIER|dnaN|MPN001|t…
4          p2_2_S1   2  CAAC|upstream_gene_variant|MODIFIER|dnaN|MPN00…
…              …  ..                                                …
41166  p3IPTG_A_S1b  18  GACGC|missense_variant|MODERATE|soj|MPN688|tra…
41167  p3IPTG_A_S1b  18  G|synonymous_variant|LOW|soj|MPN688|transcript…
41168  p3IPTG_A_S1b  18  G|missense_variant|MODERATE|soj|MPN688|transcr…
```

```
41169  p3IPTG_A_S1b  18  CTTTC|missense_variant|MODERATE|soj|MPN688|tra…
41170  p3IPTG_A_S1b  18  TGCGTGAAA|upstream_gene_variant|MODIFIER|K05_o…

[508440 rows x 11 columns]
```

[36]:

```
       ␣
↪---------------------------------------------------------------------------

       NameError                                 Traceback (most recent call␣
↪last)

       <ipython-input-36-96ce78c8031b> in <module>
         1 c = 1
         2 rs = {}
   ----> 3 for i, j in selected.iterrows():
         4     for eff in str(j.EFF).split(','):
         5         jjj = eff.split('|')


       NameError: name 'selected' is not defined
```

[184]:

```
selected
```

```
       ␣
↪---------------------------------------------------------------------------

       NameError                                 Traceback (most recent call␣
↪last)

       <ipython-input-184-19ff2ad0f5a8> in <module>
   ----> 1 selected


       NameError: name 'selected' is not defined
```

[38]:

```
selected_ann
```

```
       ␣
↪---------------------------------------------------------------------------
```

```
NameError                                 Traceback (most recent call␣
↪last)

<ipython-input-38-28fde1192669> in <module>
----> 1 selected_ann


NameError: name 'selected_ann' is not defined
```

[39]:
```
selected_ann[selected_ann['IMPACT']=='HIGH']
```

```
␣
↪---------------------------------------------------------------------------

NameError                                 Traceback (most recent call␣
↪last)

<ipython-input-39-e70eb41dc56f> in <module>
----> 1 selected_ann[selected_ann['IMPACT']=='HIGH']


NameError: name 'selected_ann' is not defined
```

4 Mutation rate study

[92]:
```
plt.close('all')
```

[ ]:
```
```

[44]:
```
pd.melt(mutrate1.sort_values('P')[[i for i in mutrate1.columns if '%' in i]])
```

```
␣
↪---------------------------------------------------------------------------

NameError                                 Traceback (most recent call␣
↪last)

<ipython-input-44-d63c0ec7e1ed> in <module>
----> 1 pd.melt(mutrate1.sort_values('P')[[i for i in mutrate1.columns if␣
↪'%' in i]])
```

```
NameError: name 'mutrate1' is not defined
```

[45]:
```
plt.figure()
plt.boxplot([mutrate1['N%'], mutrate2['N%']])
```

```
Canvas(toolbar=Toolbar(toolitems=[('Home', 'Reset original view', 'home', 'home'), ('Back', 'Ba
```

```
␣
↪---------------------------------------------------------------------------

        NameError                                 Traceback (most recent call␣
↪last)

        <ipython-input-45-05ad25cb1d90> in <module>
          1 plt.figure()
    ----> 2 plt.boxplot([mutrate1['N%'], mutrate2['N%']])


        NameError: name 'mutrate1' is not defined
```

[46]:
```
plt.close('all')
plt.figure()
x, y = ['OUT%', 'IN%']
plt.plot([0,16],[0,16], c='grey', linestyle='--')
sns.scatterplot(x=x, y=y, data=mutrate1)
for i in range(mutrate1.shape[0]):
    plt.text(x=mutrate1[x][i]+0.2,y=mutrate1[y][i]+0.3,s=mutrate1.index[i],
             fontdict=dict(size=8))
sns.scatterplot(x=x, y=y, data=mutrate2)
c=0
for i in range(mutrate2.shape[0]):

    if c==0:
        plt.text(x=mutrate2[x][i]+0.2,y=mutrate2[y][i]+0.4,s=mutrate2.index[i],
                 fontdict=dict(size=10))
        c==1
    else:
        plt.text(x=mutrate2[x][i]+0.2,y=mutrate2[y][i]+0.2,s=mutrate2.index[i],
                 fontdict=dict(size=8))
        c==0
plt.xlabel('Genome Variant Rate [%]')
plt.ylabel('Cassette Variant Rate [%]')
plt.xlim(0,16)
plt.ylim(0,16)
```

```
Canvas(toolbar=Toolbar(toolitems=[('Home', 'Reset original view', 'home', 'home'), ('Back', 'Ba
```

```
        ␣
↪---------------------------------------------------------------------------

       NameError                                 Traceback (most recent call␣
↪last)

       <ipython-input-46-c03ce5ec3171> in <module>
         3 x, y = ['OUT%', 'IN%']
         4 plt.plot([0,16],[0,16], c='grey', linestyle='--')
    ----> 5 sns.scatterplot(x=x, y=y, data=mutrate1)
         6 for i in range(mutrate1.shape[0]):
         7     plt.text(x=mutrate1[x][i]+0.2,y=mutrate1[y][i]+0.3,s=mutrate1.
↪index[i],

       NameError: name 'mutrate1' is not defined
```

[47]:
```
from scipy.stats import wilcoxon, ttest_rel
from collections import Counter

wrs = {}
trs = {}
ns = dict(Counter(mutrate.P))
for p in set(mutrate.P):
    wrs[p] = wilcoxon(x=list(mutrate[mutrate['P']==p]['OUT%']),
                      y=list(mutrate[mutrate['P']==p]['IN%']))[1]
    trs[p] = ttest_rel(list(mutrate[mutrate['P']==p]['OUT%']),
                       list(mutrate[mutrate['P']==p]['IN%']))[1]
```

```
        ␣
↪---------------------------------------------------------------------------

       NameError                                 Traceback (most recent call␣
↪last)

       <ipython-input-47-8c5c8001c3c9> in <module>
         4 wrs = {}
         5 trs = {}
    ----> 6 ns = dict(Counter(mutrate.P))
         7 for p in set(mutrate.P):
         8     wrs[p] = wilcoxon(x=list(mutrate[mutrate['P']==p]['OUT%']),
```

```
NameError: name 'mutrate' is not defined
```

[48]:
```
ns, wrs, trs
```

```
↪---------------------------------------------------------------------------

NameError                                 Traceback (most recent call
↪last)

<ipython-input-48-5ec18e92d2c6> in <module>
----> 1 ns, wrs, trs

NameError: name 'ns' is not defined
```

null hypothesis (same mut rate) cannot be rejected at a confidence level of 5%, only at early passages 2 and 3 seems to be limited

[49]:
```
snpcalls50
```

```
↪---------------------------------------------------------------------------

NameError                                 Traceback (most recent call
↪last)

<ipython-input-49-bf7c51358a6e> in <module>
----> 1 snpcalls50

NameError: name 'snpcalls50' is not defined
```

5 Plot variations

[50]:
```
cass_par
```

[50]: [565510, 572124]

[51]:
```
selected
```

```
    ␣
↪---------------------------------------------------------------------------

      NameError                                 Traceback (most recent call␣
↪last)

      <ipython-input-51-19ff2ad0f5a8> in <module>
  ----> 1 selected


      NameError: name 'selected' is not defined
```

[52]:
```
from reportlab.lib import colors
from reportlab.lib.units import cm
from Bio.Graphics import GenomeDiagram


def plot_variations(genbank_file, coords, variations):
    record = SeqIO.read(genbank_file, "genbank")

    gd_diagram = GenomeDiagram.Diagram(record.id)
    gd_track_for_features = gd_diagram.new_track(1, name="Annotated Features")
    gd_feature_set = gd_track_for_features.new_set()

    for feature in record.features:
        if feature.location.start>=coords[0] and feature.location.
↪start<=coords[1] and feature.type!='Polymorphism':
            print(feature.type, feature.qualifiers['label'], feature.location)
            feature.location.strand=-1
            #if feature.type != "gene":
                # Exclude this feature
            #    continue
            #if len(gd_feature_set) % 2 == 0:
            #    color = colors.blue
            if feature.type=='CDS':
                color = colors.lightblue
                gd_feature_set.add_feature(
                    feature, sigil="ARROW", arrowshaft_height=1.0, color=color,␣
↪height=0.2, label=True, label_size=14, label_angle=0
                )
            # else:
            #     color = colors.lightblue
            #     gd_feature_set.add_feature(
            #         feature, sigil="BOX", color=color, height=0.1,␣
↪label=True, label_size=14, label_angle=10)
```

```
    colordic = {'stop': colors.firebrick, 'frameshift':colors.red, 'missense':
↪colors.orange}
    done = []

    for pos, eff in zip(variations.POS, variations.EFF):
        if pos not in done:
            effi = eff.split('|')[1].split('_')[0]
            color = colordic[effi]
            feature = SeqFeature(FeatureLocation(pos, pos+1))
            gd_feature_set.add_feature(
                    feature,
                    color=color,
                    name=' '+effi,
                    label=True,
                    label_size=15,
                    size=20
                    #label_color=color,
                )
            done.append(pos)
            for i in range(pos-30, pos+30):
                done.append(i)
    gd_diagram.draw(format="linear", pagesize="A5", fragments=1, start=565510,␣
↪end=572124)
    gd_diagram.write("plasmid_linear_nice.svg", "SVG")
```

[53]:

```
plot_variations('./data/C5_refSeq.gb', coords=[565549,571818],␣
↪variations=selected)
```

```
       ␣
↪---------------------------------------------------------------------------

       NameError                                 Traceback (most recent call␣
↪last)

       <ipython-input-53-3c5b915f50d3> in <module>
  ----> 1 plot_variations('./data/C5_refSeq.gb', coords=[565549,571818],␣
↪variations=selected)


       NameError: name 'selected' is not defined
```

[653]:

```

```

[637]:
```
raw_seq, new_seq = get_new_sequence(seq_record5, 'LacI4', pos=[565576, 'AGGC',␣
↪'GGGT'])
yyy = dnds(raw_seq, new_seq)
```

```
268.6666666666668
```

[654]:
```

```

[648]:
```
effect1[effect1['POS']==571815]
```

[648]:
```
      SAMPLE  PASS     POS  QUAL  TOT  REFN  ALTN      FRAC         REF  \
2140  p3_D_S2    3  571815   0.0  674   668     6  0.898204  CCAGATGTAA

             ALT         EFF IMPACT    AFF          MUT
2140  TCTGTTGTTT  start_lost   HIGH  cas9B  p.LeuAsp1?
```

[656]:
```
for k, v in ttt.items():
    print(k, v[-1])
```

```
p3IPTG_A_S1 2.404754044239023
p3_D_S2 1.7427616926503313
p15_F_S1 2.3935483870967738
p15_F_S5 3.4130506790327924
p3IPTG_A_S1b 2.6608581600149894
p3_D_S3 2.117820324005891
p2_2_S3 2.3640178337267255
p2_2_S1 2.127371273712737
p2_2_S2 1.7000000000000002
```

[620]:
```

```

[56]:
```
df = pd.read_csv(, header=60, sep='\t')
df2 = df[(df['INFO'].str.contains('CIGAR=1X;'))].sample(n=150, random_state=1).
↪copy()
```

```
  File "<ipython-input-56-9a8f373c509c>", line 1
    df = pd.read_csv(, header=60, sep='\t')
                     ^
SyntaxError: invalid syntax
```

[57]:
```
pn, ps, pns, dn, ds, dns = 0, 0, 0, 0, 0, 0
for item in sorted(output[chromosome]):
    raw_seq, new_seq = get_new_sequence(args.reference, chromosome,␣
↪output[chromosome][item][0].qualifiers[
```

```
        'locus_tag'][0], output[chromosome][item][1])
    newpn, newps, newdn, newds = dnds(raw_seq, new_seq)
    pn += newpn
    ps += newps
    dn += newdn
    ds += newds
if pn == 0 and ps == 0:
    pns, dns = '/', '/'
elif ps == 0:
    pns, dns = '+', '+'
elif pn == 0:
    pns, dns = '-', '-'
else:
    pns, dns = pn / ps, dn / ds
print args.query, chromosome, pn, ps, pns, dn, ds, dns
```

```
  File "<ipython-input-57-012244273d6f>", line 18
    print args.query, chromosome, pn, ps, pns, dn, ds, dns
             ^
SyntaxError: Missing parentheses in call to 'print'. Did you mean print(args.
↪query, chromosome, pn, ps, pns, dn, ds, dns)?
```

[58]:
```
for records in SeqIO.parse(, "genbank"):
    print(/)
    raw_seq, new_seq = get_new_sequence('./data/C5_refSeq.gb', 'C5',␣
 ↪output[chromosome][item][0].qualifiers[
        'locus_tag'][0], output[chromosome][item][1])
```

```
  File "<ipython-input-58-9fd2afaa1715>", line 1
    for records in SeqIO.parse(, "genbank"):
                               ^
SyntaxError: invalid syntax
```

[ ]:
```
```

[ ]:
```
```
