# Supplementary material for: A genetic toolkit and gene switches to limit Mycoplasma growth for biosafety applications
Source: Nat Commun. 2022 Apr 7;13:1910. doi: 10.1038/s41467-022-29574-0 (PMC8991246; doi:10.1038/s41467-022-29574-0)

A genetic toolkit and gene switches to limit *Mycoplasma* growth for a synthetic vaccine chassis

Draft Nat comms

uncropped WB and DNA gels

Fig. 1D – uncropped WB

Primary Ab: mouse monoclonal 8C5.5 anti-mCherry (BioLegend 677702, 1:1000 dilution) Ab
Secondary Ab: Polyclonal anti-mouse IgG (Jackson Immune Research 515-035-003, 1:5000 dilution) Ab conjugated to horseradish peroxidase

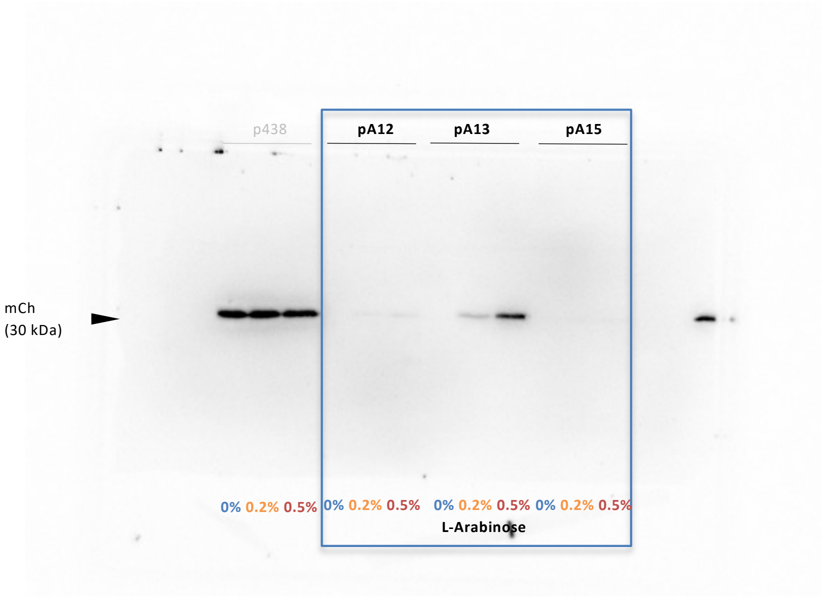

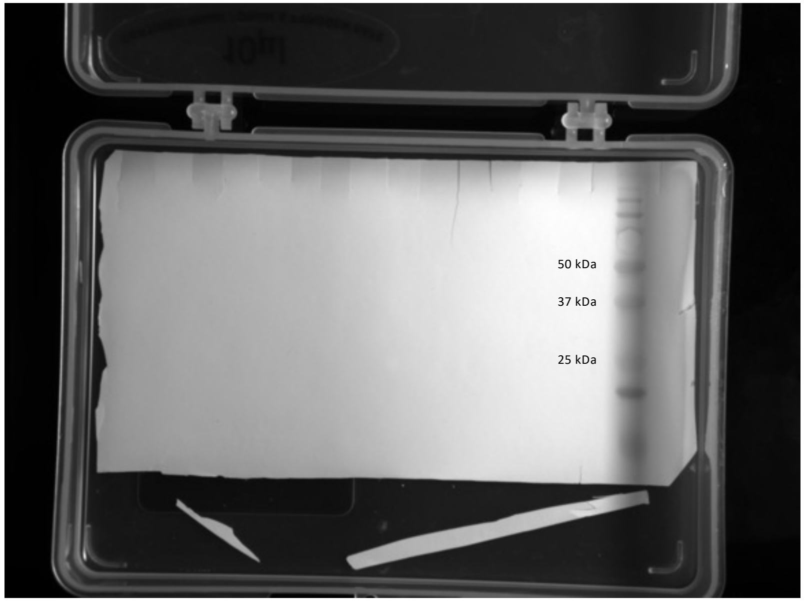

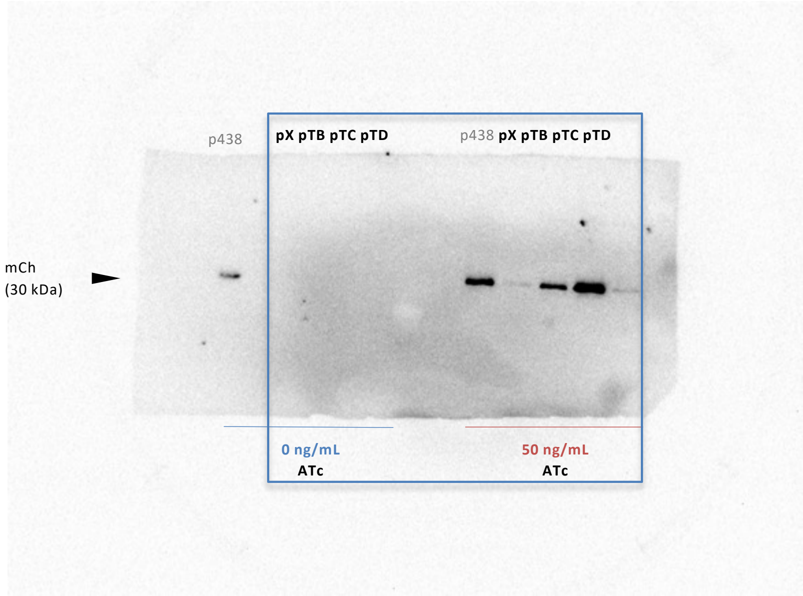

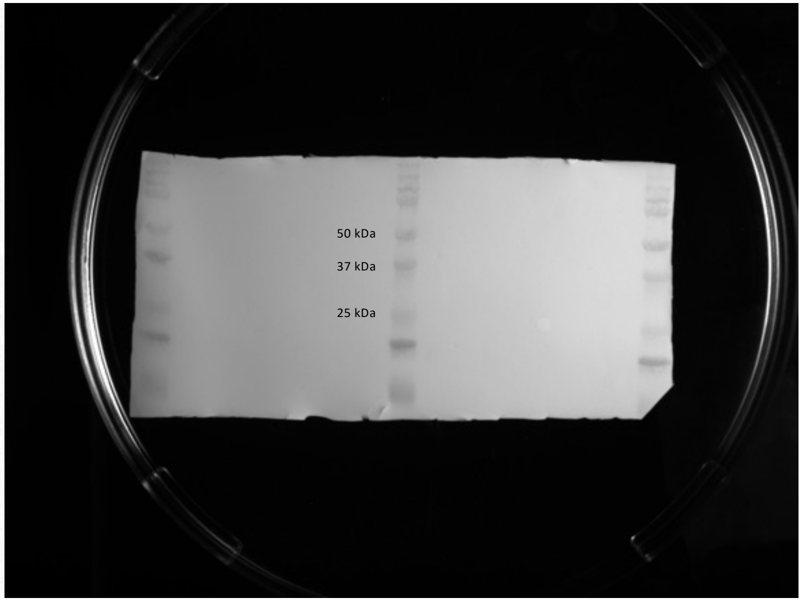

Fig. 1D – uncropped WB

Primary Ab: mouse monoclonal 8C5.5 anti-mCherry (BioLegend 677702, 1:1000 dilution) Ab
Secondary Ab: Polyclonal anti-mouse IgG (Jackson Immune Research 515-035-003, 1:5000 dilution) Ab conjugated to horseradish peroxidase

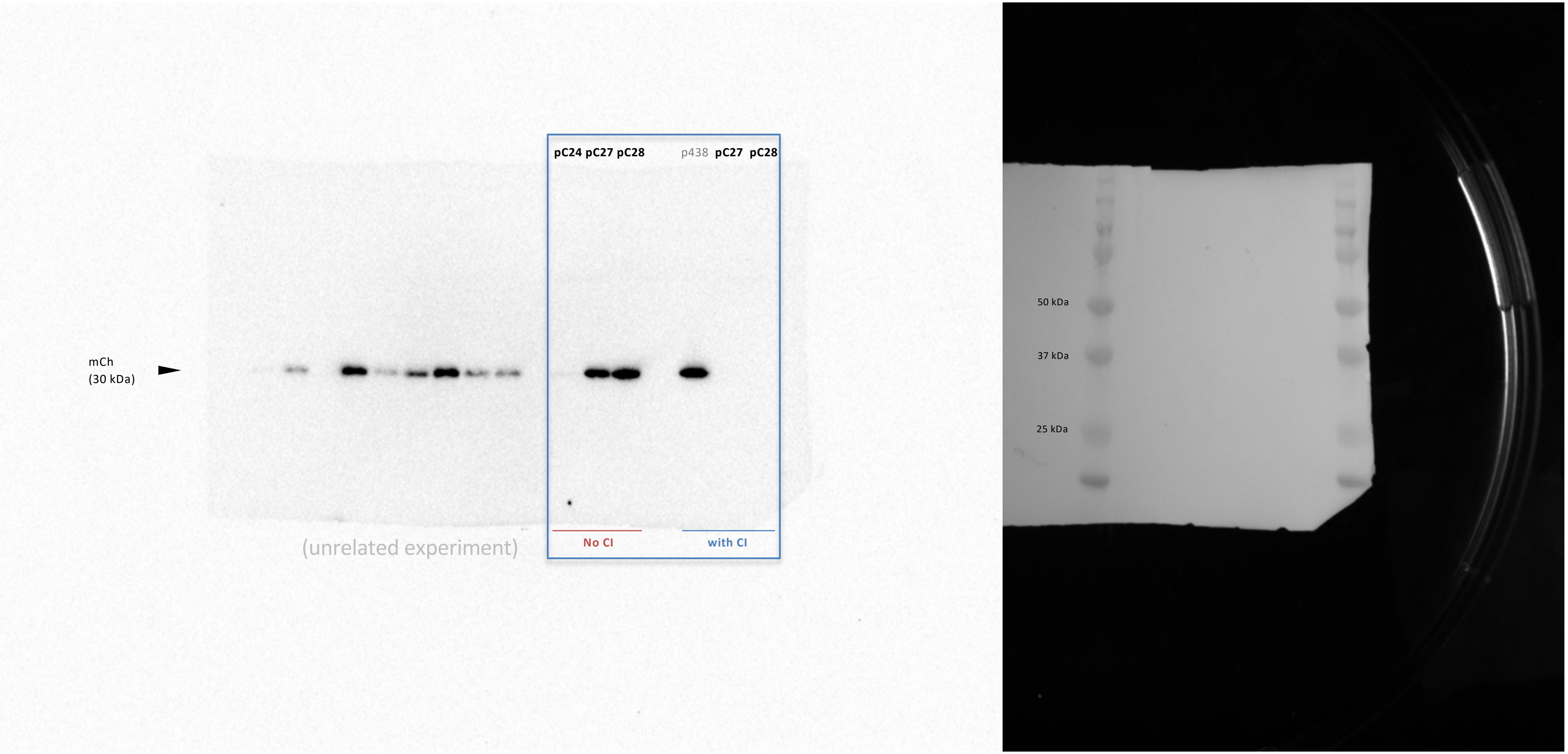

Fig. 1D – uncropped WB

Primary Ab: mouse monoclonal 8C5.5 anti-mCherry (BioLegend 677702, 1:1000 dilution) Ab

Secondary Ab: Polyclonal anti-mouse IgG (Jackson Immune Research 515-035-003, 1:5000 dilution) Ab conjugated to horseradish peroxidase

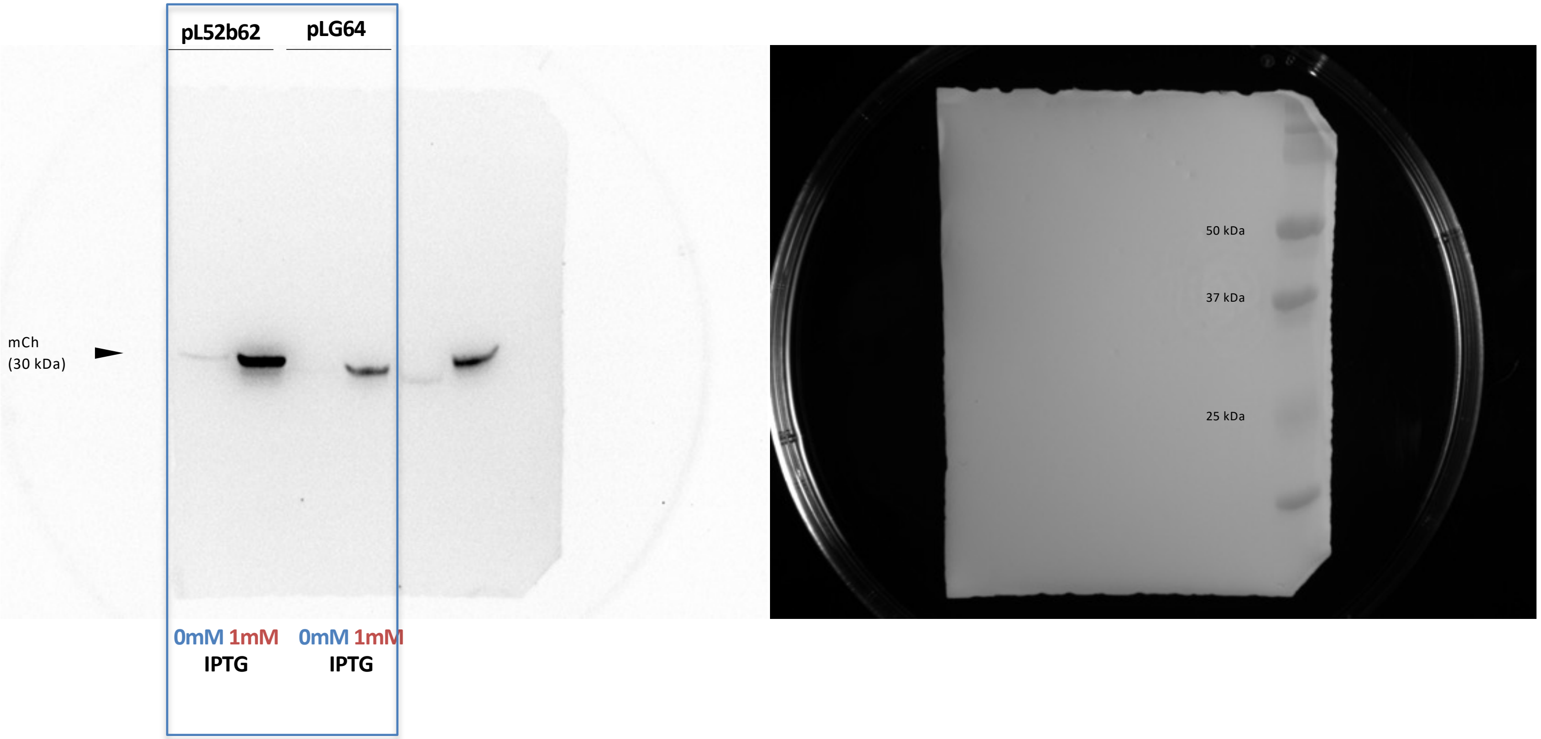

Fig. 2C – uncropped WB

Primary Ab: mouse monoclonal 7A9 anti-Cas9 (BioLegend 844301, 1:1000 dilution) Ab
Secondary Ab: Polyclonal anti-mouse IgG (Jackson Immune Research 515-035-003, 1:5000 dilution) Ab conjugated to horseradish peroxidase

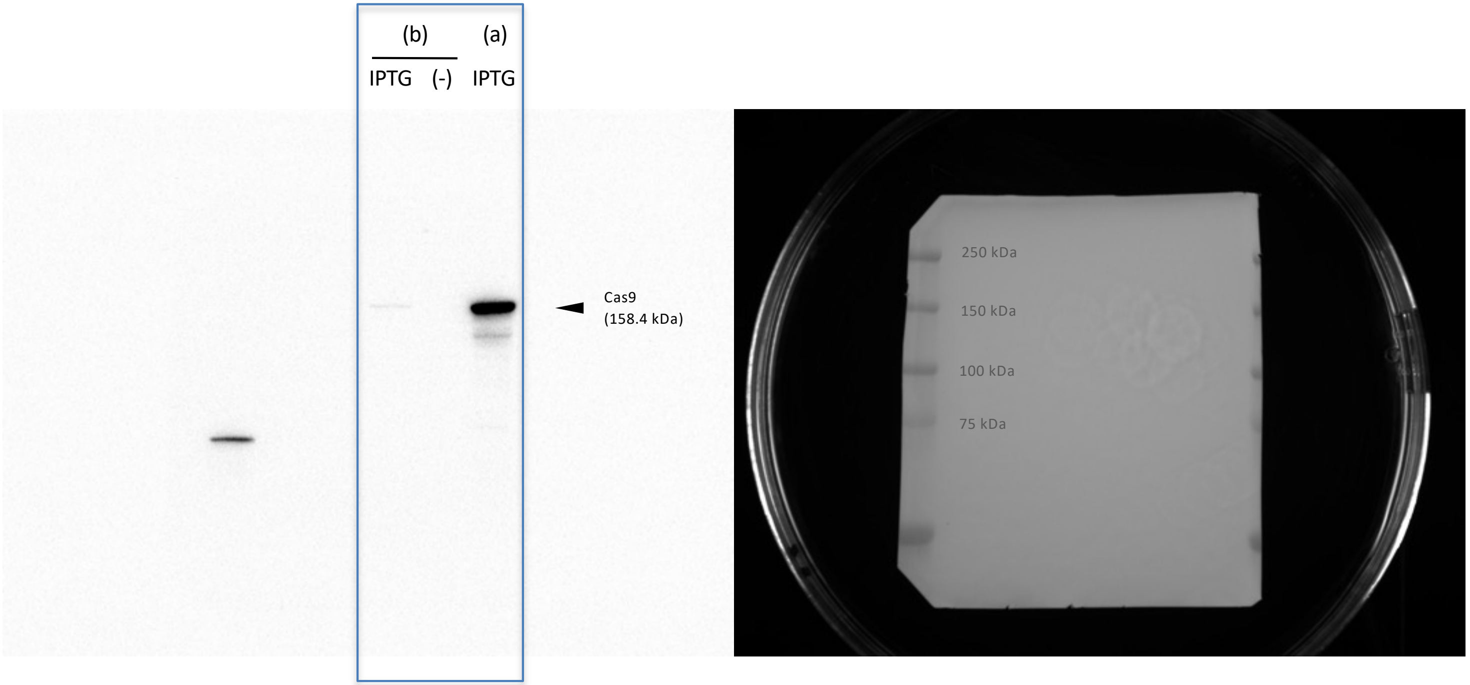

Fig. 3C – uncropped DNA gels

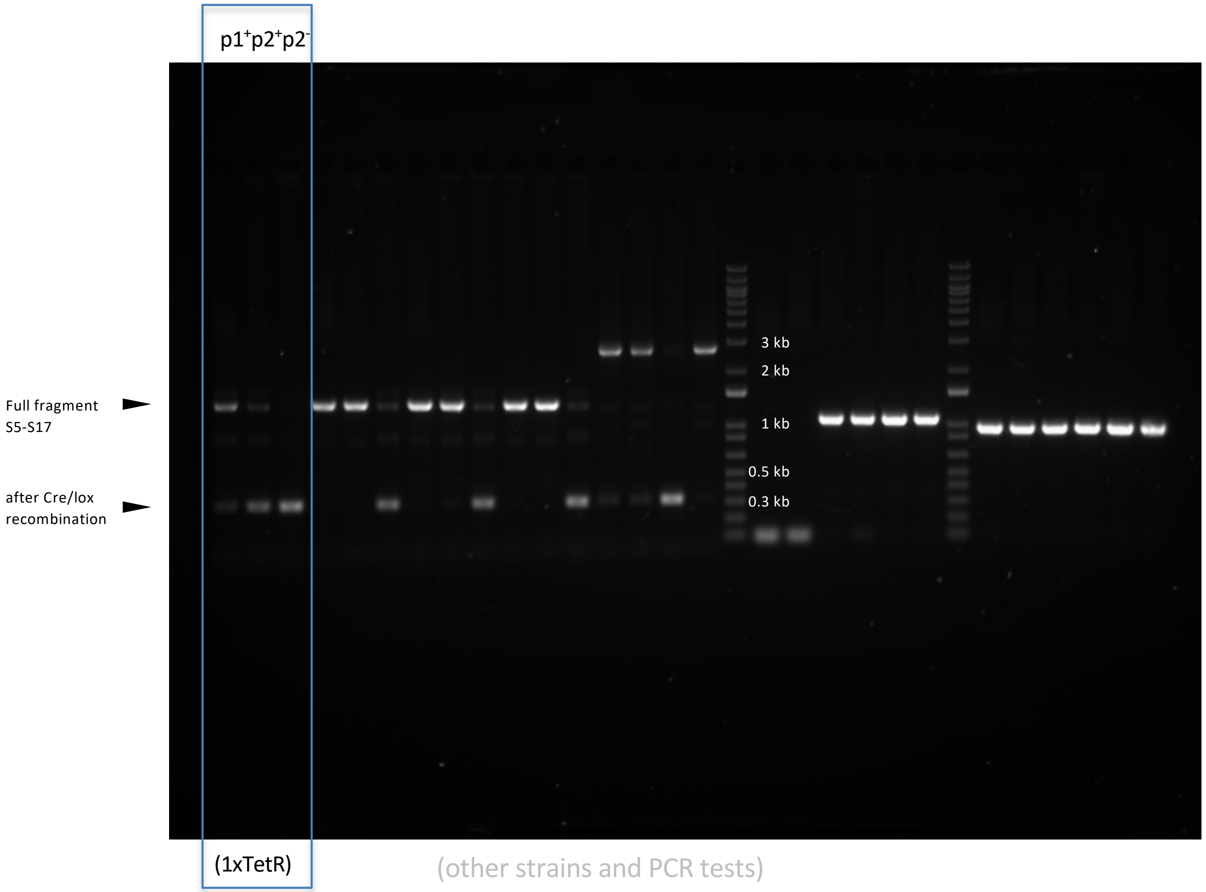

Fig. 3C – uncropped DNA gels

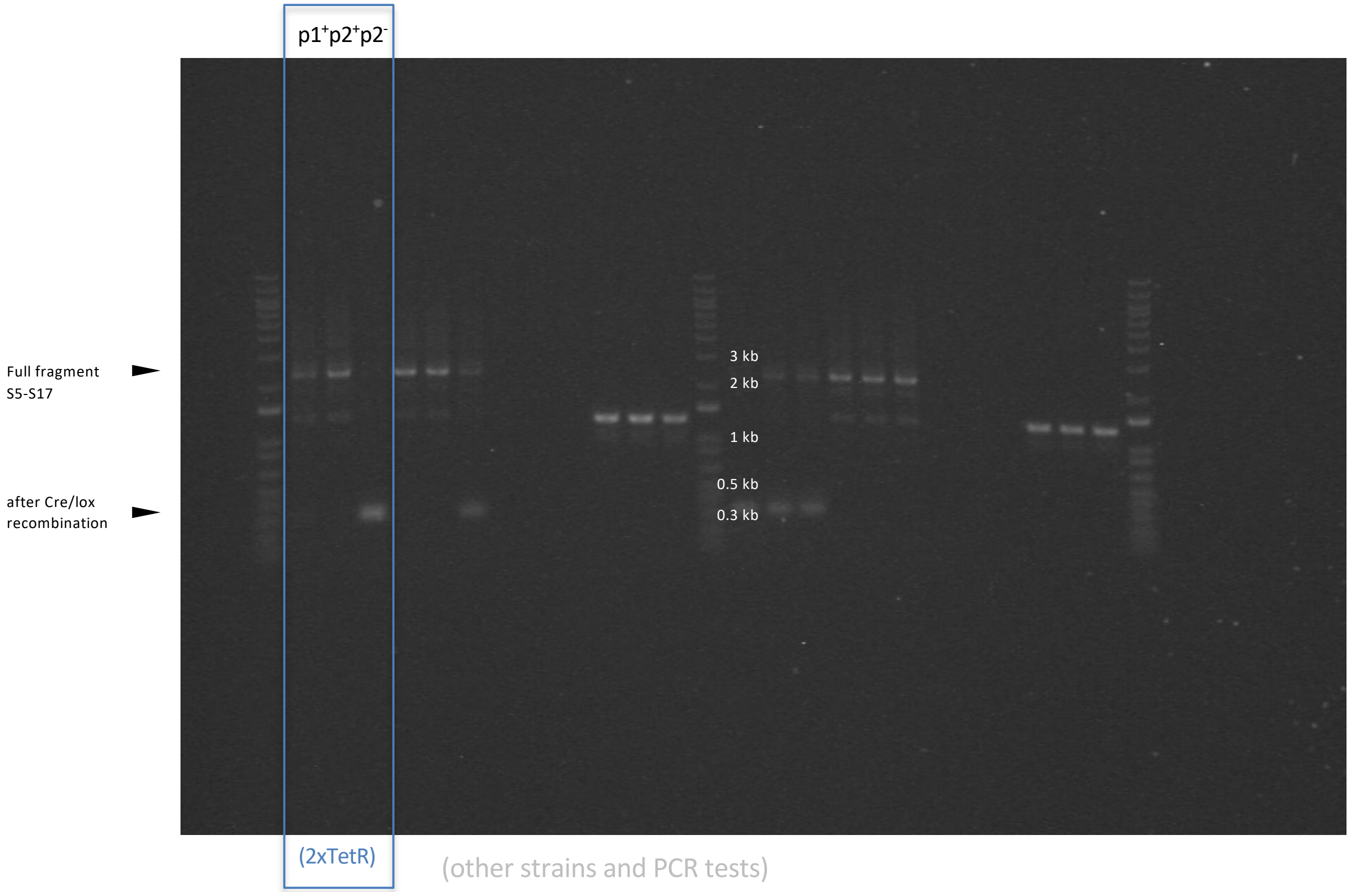

Fig. 3D – uncropped WB

Primary Ab: rabbit polyclonal anti-TetR (Sigma T0951, 1:1000 dilution) Ab

Secondary Ab: polyclonal anti-rabbit IgG (Jackson Immune Research 111-035-003, 1:5000 dilution) Ab conjugated to horseradish peroxidase

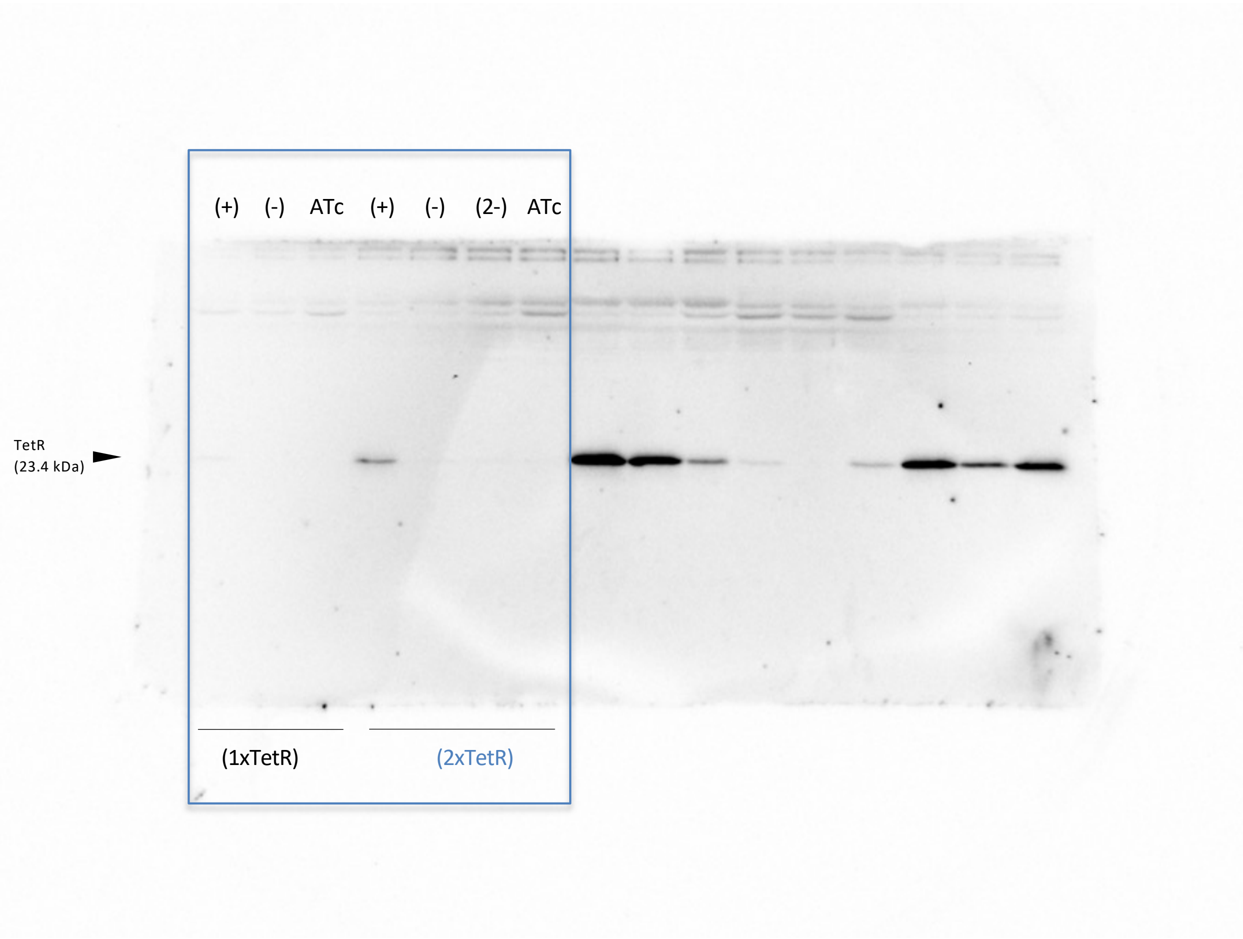

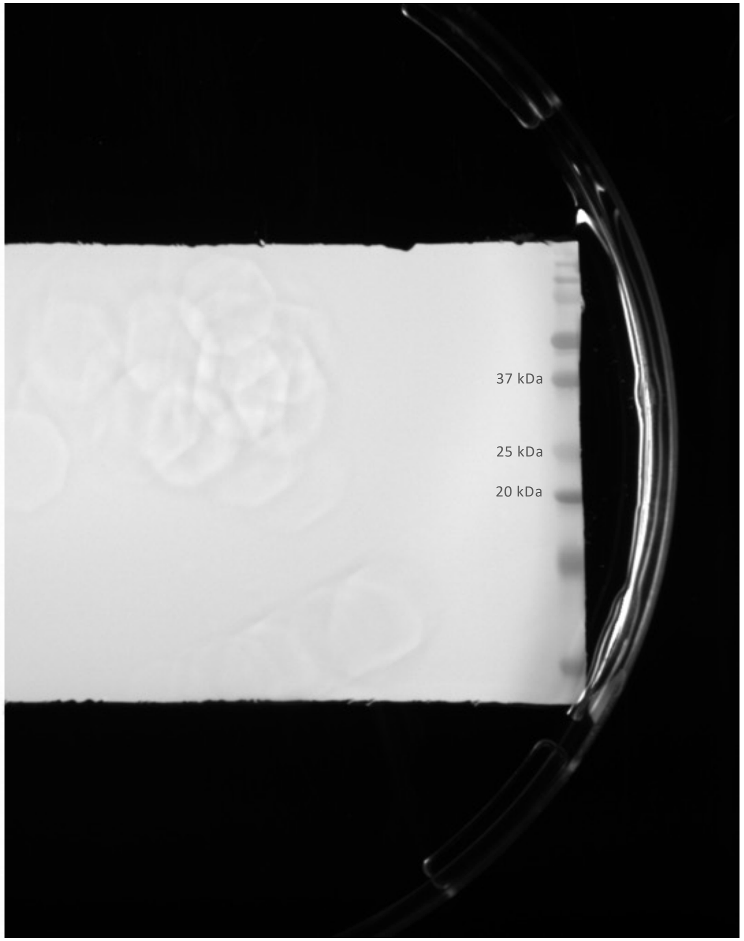

Fig. 3D – uncropped WB

Primary Ab: mouse monoclonal 9E10 anti-cMyc (Sigma M4439, 1:2000 dilution) Ab to detect CI-cMyc
Secondary Ab: Polyclonal anti-mouse IgG (Jackson Immune Research 515-035-003, 1:5000 dilution) Ab conjugated to horseradish peroxidase

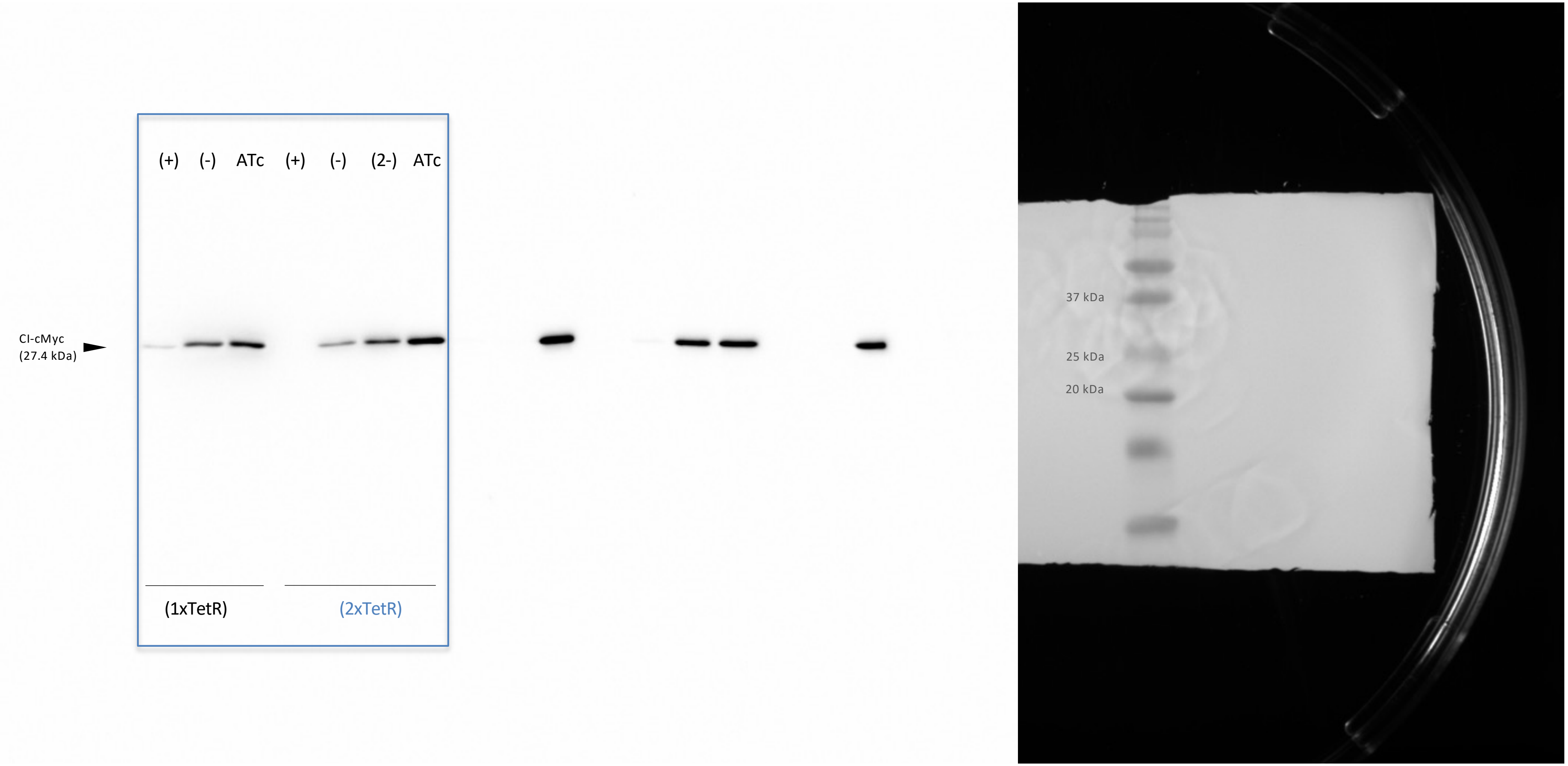

Fig. 3D – uncropped WB

Primary Ab: mouse monoclonal 7A9 anti-Cas9 (BioLegend 844301, 1:1000 dilution) Ab
Secondary Ab: Polyclonal anti-mouse IgG (Jackson Immune Research 515-035-003, 1:5000 dilution) Ab conjugated to horseradish peroxidase

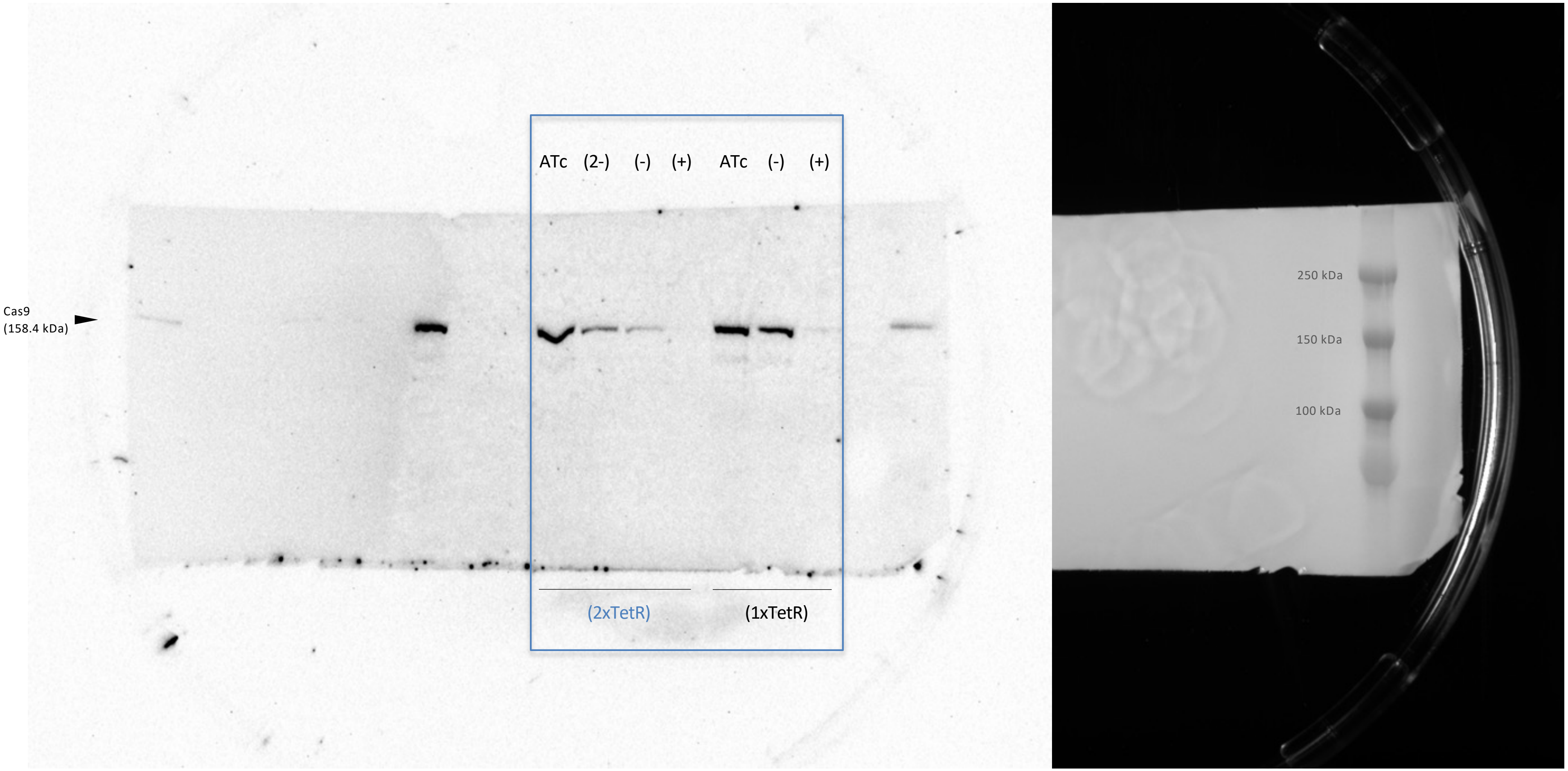

Supplement: Supplementary file 10 — Source Data [file 41467_2022_29574_MOESM10_ESM.zip › Source Data/Uncroppedimages_Mycotoolsandcircuits.pdf]
